# The collagenase-induced osteoarthritis (CIOA) model: Where mechanical damage meets inflammation

**DOI:** 10.1016/j.ocarto.2024.100539

**Published:** 2024-10-24

**Authors:** Patrick Weber, Kajetana Bevc, David Fercher, Sami Kauppinen, Shipin Zhang, Maryam Asadikorayem, Lucia Baixauli Marin, Tanqi Zhang, Tuomas Frondelius, Gian Salzmann, Valentino Bruhin, Jakob Hax, Gonçalo Barreto, Mikko A.J. Finnilä, Marcy Zenobi-Wong

**Affiliations:** a Tissue Engineering + Biofabrication Laboratory, Department of Health Sciences and Technology, ETH Zürich, Otto-Stern-Weg 7, 8093 Zurich, Switzerland; b Research Unit of Health Sciences and Technology, University of Oulu, Aapistie 5A, 90220, Oulu, Finland; c Schulthess Klinik, Department for Knee Surgery, Lengghalde 2, 8008 Zurich, Switzerland; d Faculty of Medicine, University of Zurich, Pestalozzistrasse 3, 8032 Zurich, Switzerland; e Clinicum, Faculty of Medicine, University of Helsinki and Helsinki University Hospital, Haartmaninkatu 8, 00290, Helsinki, Finland; f Biocenter Oulu, University of Oulu, Aapistie 5A, 90220, Oulu, Finland

**Keywords:** Osteoarthritis, Animal model, Collagenase, Synovitis, Computed tomography

## Abstract

**Objective:**

To characterize inflammatory and mechanical changes in the collagenase-induced OA (CIOA) model in rats.

**Design:**

Skeletally mature, 6-month-old Wistar rats received unilateral intraarticular injections of saline, 500 U or 1000 U of collagenase on days 0 and 2 of the study. Joint tissues were harvested on either day 4 or 70 to evaluate the acute and long-term changes. Blood biomarkers, gait asymmetry and mechanical hyperalgesia were assessed repeatedly up until day 70.

**Results:**

The intraarticular injection of collagenase triggered an increase in cartilage degeneration and bone resorption over time, particularly for 1000 U. Similarly, mild synovitis was observed on day 70 with an increased number of synovial lining cells, increased fibrosis, and infiltration of peripheral macrophages. Mechanistically, these findings were linked to a dose-related mechanical weakening of the anterior cruciate ligament (ACL), which caused persistent joint destabilization throughout the study. Furthermore, the collagenase injection triggered acute inflammation and swelling of the synovium on day 4 and an acute systemic inflammatory response with increased cytokine and peripheral blood immune cell levels. While mild synovitis persisted until day 70, the systemic inflammatory response returned to control levels after 8 days. Similarly, the observed acute changes in gait and mechanical hyperalgesia also returned to baseline after 21 days.

**Conclusion:**

By evaluating inflammatory and mechanical factors at different doses and timepoints, our characterization enables a more targeted study design and increases the clinical relevance of future studies involving the CIOA model.

## 1 Introduction

Osteoarthritis (OA) is one of the leading causes of pain and disability worldwide, affecting approximately 30 ​% of all people above the age of 65 [1]. Though the last decades have witnessed the development of many promising disease-modifying drugs at the preclinical stage, they all failed to show any clinically relevant improvements in a human OA population [[2], [3], [4]]. This questions the clinical relevance of our current preclinical animal models, which prioritize simplicity and reproducibility and thus often fail to capture the complex clinical reality [5]. OA is increasingly understood as a highly heterogenous, multi-tissue disease [[6], [7], [8]] for which a universal therapeutic solution is unlikely to ever exist. To support more targeted treatment approaches, there is therefore an urgent need for a better characterization of OA animal models.

There exists a plethora of different preclinical OA animal models, all with their own advantages and disadvantages [9,10]. Large animal models of OA including horses, sheep and goats are considered the gold standard due to comparable cartilage thickness and joint loading as in humans [9,10]. On the other hand, they are very costly and typically develop on relatively long timescales which makes rodent models the preferred option for a first evaluation [9,10]. Though mice are known to develop OA spontaneously with age, rodent models of OA more commonly rely on an artificial induction mechanism [9,10]. Within the class of induced models, surgical models like the anterior cruciate ligament (ACL) transection (ACLT) model aim to destabilize the joint by transecting or removing one of the stabilizing tissues of the joint, which induces cartilage degeneration within a few weeks [11,12]. Though surgical OA models today comprise the bulk of all OA preclinical research [13], they require highly trained personnel and have a high operator dependence [14]. One alternative are non-invasive models, in which cartilage and/or ligaments are injured by external mechanical overloading of the joint [15]. Using force sensors, the severity of the injury can be controlled, leading to reproducible OA-like changes without any surgical intervention [15,16]. Another option are chemically induced models which only require a minimally invasive intraarticular injection and provide control over the severity by changing the dose of the injected chemical. Besides monoiodoacetate, which triggers cell death in exposed joint tissues and rapidly induces a very severe OA-like phenotype [17] with a strong pain component [18] and limited similarity to human OA patients [19], the use of collagenase is becoming more popular in the field.

Initially described by *van der Kraan* et al. in 1990, the collagenase-induced OA (CIOA) model aims to enzymatically weaken the intra-capsular ligaments to induce OA-like changes through similar mechanisms as in the surgical models [20]. At the same time, the injection of collagenase also triggers an inflammatory response in the synovium that remains elevated in the long term and further contributes to the degenerative changes [21,22]. This combination of mechanical and inflammatory factors is a great advantage as these two factors are understood to be closely intertwined in the human disease [23,24]. Recently, several studies characterizing dose-dependence [22,25], sex-differences [26,27], synovitis [21,28,29], immune cells [29] and gait changes [25] of the CIOA model have been reported. However, these studies use different collagenase concentrations (1–900 U) and animal species (mouse, rat, rabbit), which makes comparison of their findings challenging.

**Fig. 1:**
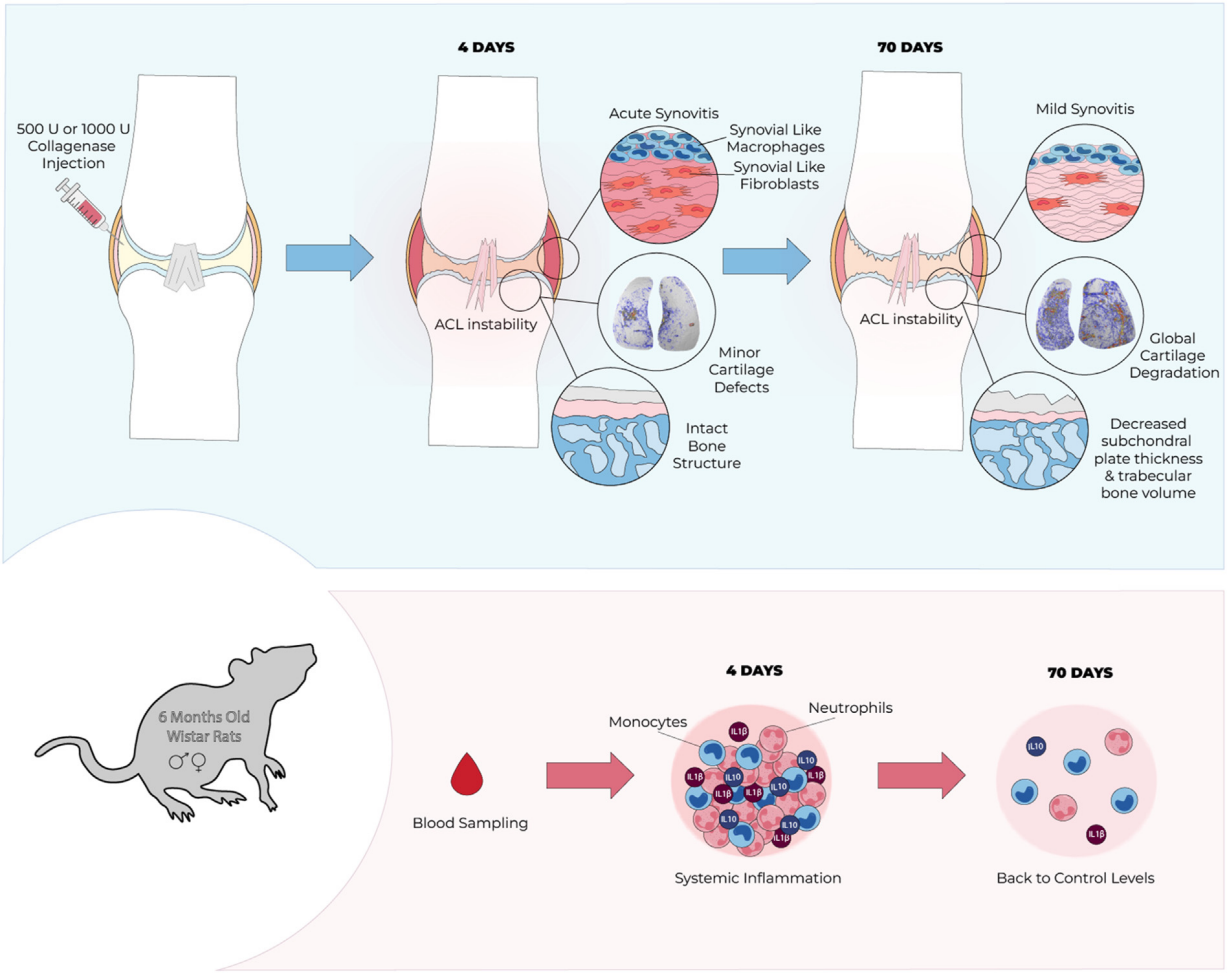
**Study Overview:** In our study, we subjected a mixed-sex population of skeletally mature Wistar rats to intraarticular collagenase injections at two different doses (500 U, 1000 U). Local and systemic changes were assessed on days 4 and 70. We observed a dose-related weakening of the ACL, acute inflammation of the synovium, and an increase in cytokines and immune cells in circulation on day 4. While the systemic inflammatory response went back to control levels on day 70, the synovium underwent fibrotic remodeling, subchondral and trabecular bone was resorbed and the cartilage showed increased degeneration that could all be associated with the initial dose.

In this study, we aimed to characterize the CIOA model in skeletally mature, outbred Wistar rats (mixed sex, 6 months old) focusing on both the mechanical and inflammatory components of the model (Fig. 1). This animal population was chosen to increase reproducibility (through skeletal maturity, outbreeding) and better represent the human OA population (mixed sex, increased age). To decouple the acute local effects from the long-term OA-like changes, joint tissues (cartilage, synovium, ACL) were harvested and analyzed after either 4 or 70 days. Up until day 70, we continuously analyzed blood biomarkers and monitored changes in gait and mechanical hyperalgesia. To evaluate the effect of dose on our observations, we investigated two different doses of collagenase (500 and 1000 U). Our results showed dose-related levels cartilage degeneration, synovitis and bone resorption on day 70. While minor differences in cartilage degeneration were already observed at day 4 for the two doses, the severe synovial inflammation on day 4 was dose-independent, suggesting crosstalk between the mechanical and inflammatory factors as the model progresses. Moreover, we found the ACL to be more substantially weakened in the 1000 U group throughout the study, causing substantial joint instability. Finally, systemic levels of peripheral blood immune cells were elevated during the acute phase and are potentially linked to the changes in cell composition in the synovium we observed on day 70 (Fig. 1).

## 2 Materials and methods

### 2.1 Chemicals

Unless otherwise stated, all chemicals were purchased from Sigma-Aldrich.

### 2.2 Animal experimentation

The animal study was approved by the Veterinary Office of the Canton Zürich (License No. ZH158/2021). 24 outbred Wistar rats (12 males (486 ​g ​± ​72 ​g), 12 females (262 ​g ​± ​20 ​g), 6 months old from Janvier Labs, FRA) were allowed to acclimatize to the new housing environment for 2 weeks after arrival. Animals were housed in groups of 3 in individually ventilated cages in a standard laboratory animal environment (21 ​± ​3 ​°C and 12/12 ​h light–dark cycle), received acidified tap water (pH 2.5–3.0) and standard laboratory rodent diet (3437, KLIBA NAFAG, CH) at specific opportunistic pathogen free hygiene. Each animal represents an experimental unit.

#### 2.2.1 Collagenase-induced OA model

Animals were randomly allocated to the study groups (see supplementary methods). To investigate acute and long-term effects in our model, animals were euthanized at two timepoints (day 4, day 70, N ​= ​12 each) with three different experimental groups throughout the study (saline, 500 U collagenase, 1000 U collagenase, N ​= ​4 each). The doses were selected based on previous experience with this model [30] and available literature (Table S1). On the day of surgery (day 0), the rats were anesthetized with isoflurane (5 ​%, then 1.5 ​% to maintain anesthesia), received subcutaneous buprenorphine (0.05 ​mg/kg), and their hindlimbs were shaved and disinfected (Kodan Tincture forte, Schülke, CH). They then received an intraarticular injection of 25 ​μL of either saline, 500, or 1000 U collagenase into a randomized knee joint at 90° flexion through the patellar tendon using 30 ​G x 8 ​mm insulin syringes (BD, USA). On day 2 the procedure was repeated. Note that the collagenase used in the experiment was from a low-activity batch (Stemcell Technologies, GER) and the enzymatic activity verified with a colorimetric assay (MAK293, Sigma-Aldrich, Table S2). Note also that on day 2, one female animal from the 1000 U collagenase/day 4 group needed to be removed from the study due to an anterolateral tibial dislocation of the injected joint.

#### 2.2.2 Gait and mechanical hyperalgesia analysis

Dynamic gait analysis was performed on a Catwalk XT system (Fig. S1, Noldus, NED) [25] following manufacturer instructions on baseline day −7 before the first injection, and days 7, 21, 35, 49 and 63 of the animal study. Runs were recorded over 10 ​min for each animal and the three runs with the lowest run speed variation selected for analysis (avg. variation of selected runs: 43.0 ​%). The calculated metrics were reported as percent difference between injected and contralateral joints, relative to the baseline measurement on day −7 (Fig. S1).

After gait analysis of all animals, mechanical hyperalgesia was assessed with a Pressure Application Measurement (PAM, Fig. S2) device (Ugo Basile, ITA) [31]. The joints were squeezed mediolaterally a rate of 3 ​N/s until retraction of the limb, vocalization or after a maximum of 5 ​s. The peak force was extracted and normalized to the baseline measurement on day −7.

### 2.3 Blood analysis

Blood was collected from the sublingual veins of anesthesized animals on days 4 (acute timepoint animals), 8, 14, 28, 42, 56 and 70 (long timepoint animals) without prior fasting, processed with density gradient separation using Lymphoprep™ (Stemcell Technologies, GER) and stored at −20 ​°C (plasma) and in liquid nitrogen (Peripheral blood immune cells), respectively.

Plasma was analyzed for cytokines IL-10, IL-1β, TNFα and IL-6 with Luminex ProcartaPlex 4-plex (Invitrogen, USA) following the manufacturer's protocol and normalized by total protein quantification (Quick Start™ Bradford Protein Assay Kit, Bio-Rad, USA). Peripheral blood immune cells were analyzed on a BD Fortessa flow cytometer (BD, USA) using the antibody panel in Table S3 and gated with FlowJo 10 (Fig. S3/4A).

#### 2.3.1 Harvest of joint capsule

The peripatellar joint capsule containing the patella and parts of the synovium and the patellar tendon was cut away from the tibial tuberosity (Fig. S5), fixed in 4 ​% paraformaldehyde and prepared for histology. Subsequently, all connecting tissues apart from the ACL were removed to isolate the femur-ACL-tibia constructs.

### 2.4 Tissue analysis

#### 2.4.1 Mechanical characterization of femur-ACL-tibia constructs

Tensile mechanical properties of femur-ACL-tibia constructs were recorded on a TA.XTplus device (Stable Micro Systems, UK) with a 50 ​N load cell at a 0.03 ​mm/s extension rate. This low extension rate was chosen to decrease contributions from the viscous component and more accurately capture the integrity of the elastic collagen network [32,33]. Stiffness was calculated as the slope of the load-vs.-displacement curve between 0.45 and 0.5 ​mm extension, and the failure load was the peak force measured during the full extension measurement.

#### 2.4.2 Micro-computed tomography

After characterization of the femur-ACL-tibia constructs, the joints were fixed in 4 ​% formaldehyde for 7 days, dehydrated, stained with 1 ​% phosphotungstic acid (in 70 ​% EtOH) for 24 ​h and scanned with a μCT 45 device (Scanco Medical, CH). The scans were acquired at 55 ​kV, 72 ​μA and 4W using an 0.5 ​mm aluminum filter (4.5 ​μm voxel size). The cartilage roughness score (CRS) was calculated as described elsewhere [30]. Briefly, a custom MatLab algorithm [34] was employed to identify the cartilage surface and calculate the local surface orientation in a 28 ​μm^2^ neighborhood. Using an iterative 5th-degree polynomial fit, a smooth reference surface was constructed for each condyle. The CRS represents the angular difference between the cartilage and reference surfaces averaged over the whole condyle (Figs. S6–9). A high CRS therefore indicates increased surface roughness and cartilage damage.

A binary mask for the bone tissue was extracted from the CT datasets and the local subchondral bone plate thickness measured with BoneJ in Fiji ImageJ v1.51n and averaged over the whole condyle. For the trabecular bone analysis, the medullary volume was isolated by identifying the subchondral bone and growth plate to create an ROI for the entire tissue volume and eroding it with a 315 ​μm circular kernel. Trabecular bone parameters were calculated with CTAn 1.20.3.0 (Bruker, USA).

#### 2.4.3 Histology

The fixed peripatellar joint capsules, femurs and tibias were decalcified in aqueous 10 ​% NH_4_-EDTA solution (joint capsule: 2 weeks, femur/tibia: 4 weeks), dehydrated, paraffinized on a Milestone Logos J device (Milestone, ITA) and embedded in paraffin blocks (Fig. S5). Serial sections were prepared using a microtome (joint capsule: 5 ​μm transverse, femur/tibia: 3 ​μm coronal, HM 325, Microm, GER). Two sections from the center of the tissues with >200 ​μm spacing were selected and rehydrated for histological staining. Safranin O, Hematoxylin & Eosin, Masson's trichrome and immunostainings were performed following the detailed procedures in the supplementary methods (Figs. S10–33).

##### 2.4.3.1 Quantitative evaluation of histological sections

The thickness of the synovium, synovial cellularity and collagen density were measured with custom macros in Fiji ImageJ v1.51n (see supplementary methods) using H&E (thickness & cellularity) and Masson's trichrome (collagen) stained sections.

Immunofluorescent stainings were analyzed using a custom script in QuPath v0.4.2 using the Hoechst-staining for cell identification.

For all analyses, the values of up to 4 regions of interest (medial/lateral x proximal/distal) per synovium were averaged (Fig. S34).

### 2.5 Histopathological grading

Based on previous experience with the CIOA model in terms of technical feasibility and quality of results, histopathological grading of synovium and cartilage was performed employing the “synovitis score” and the “cartilage degeneration score” as proposed by *Krenn* et al. [35] and the OARSI histopathology initiative [36], respectively. The synovium was graded by 3 and femur/tibia by 4 blinded graders. The reported values represent the average of the graders.

### 2.6 Statistical analysis

**Fig. 2:**
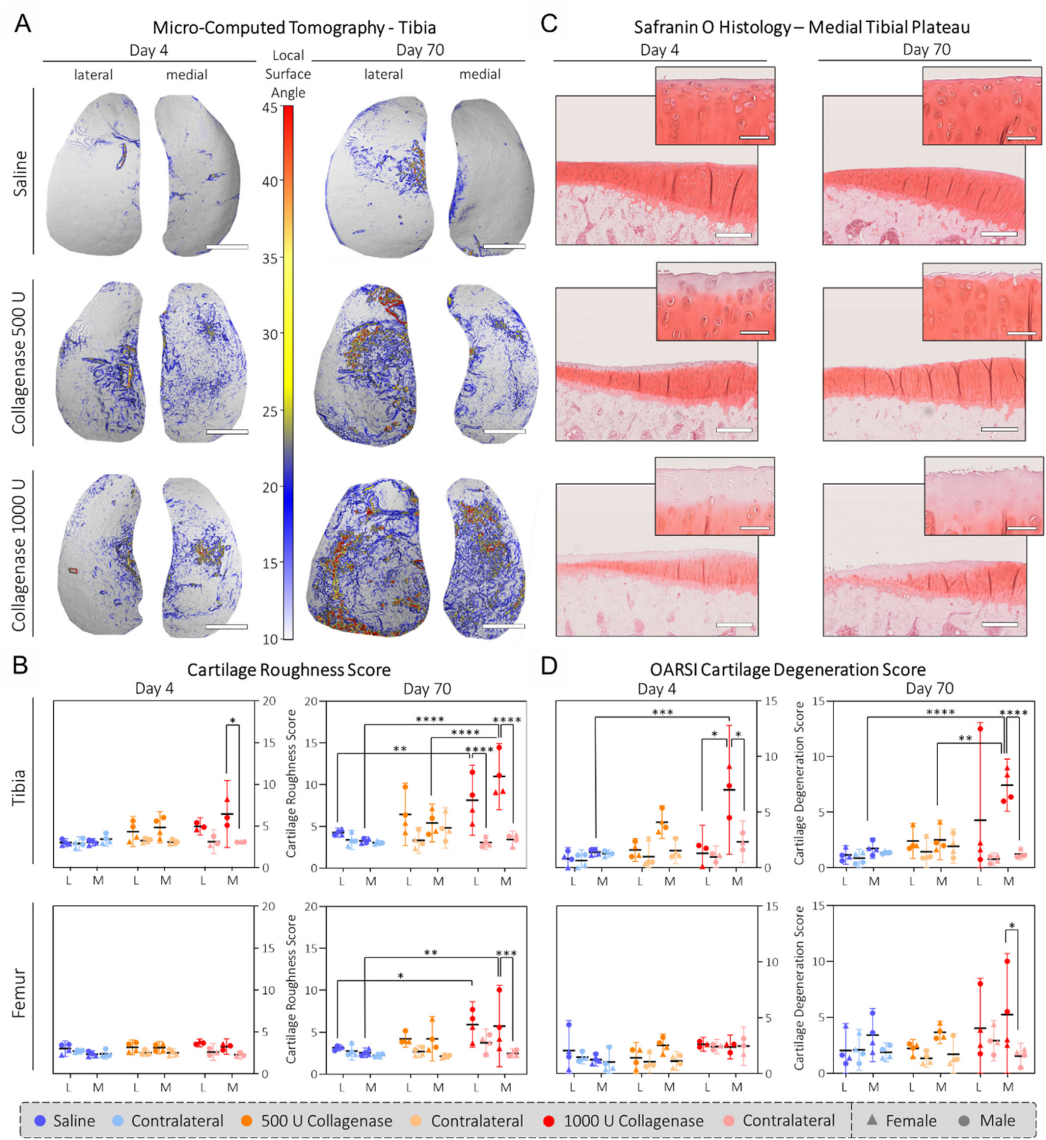
**Collagenase causes dose-related cartilage degeneration:** A) Representative μCT local surface roughness maps of tibial plateaus of our three experimental groups. Scale bar: 1 ​mm ​B) Quantitative comparison of the CRS of tibia and femur at the two different timepoints. C) Safranin O staining of medial tibial plateaus that were graded to allow for (D) the semi-quantitative analysis using the OARSI CDS. Scale bar: 250 ​μm, 50 ​μm (insert). L ​= ​lateral, M ​= ​medial. N ​= ​4 (N ​= ​3 for day 4/1000 U collagenase). Full dataset available in Figs. S6–21.

**Fig. 3:**
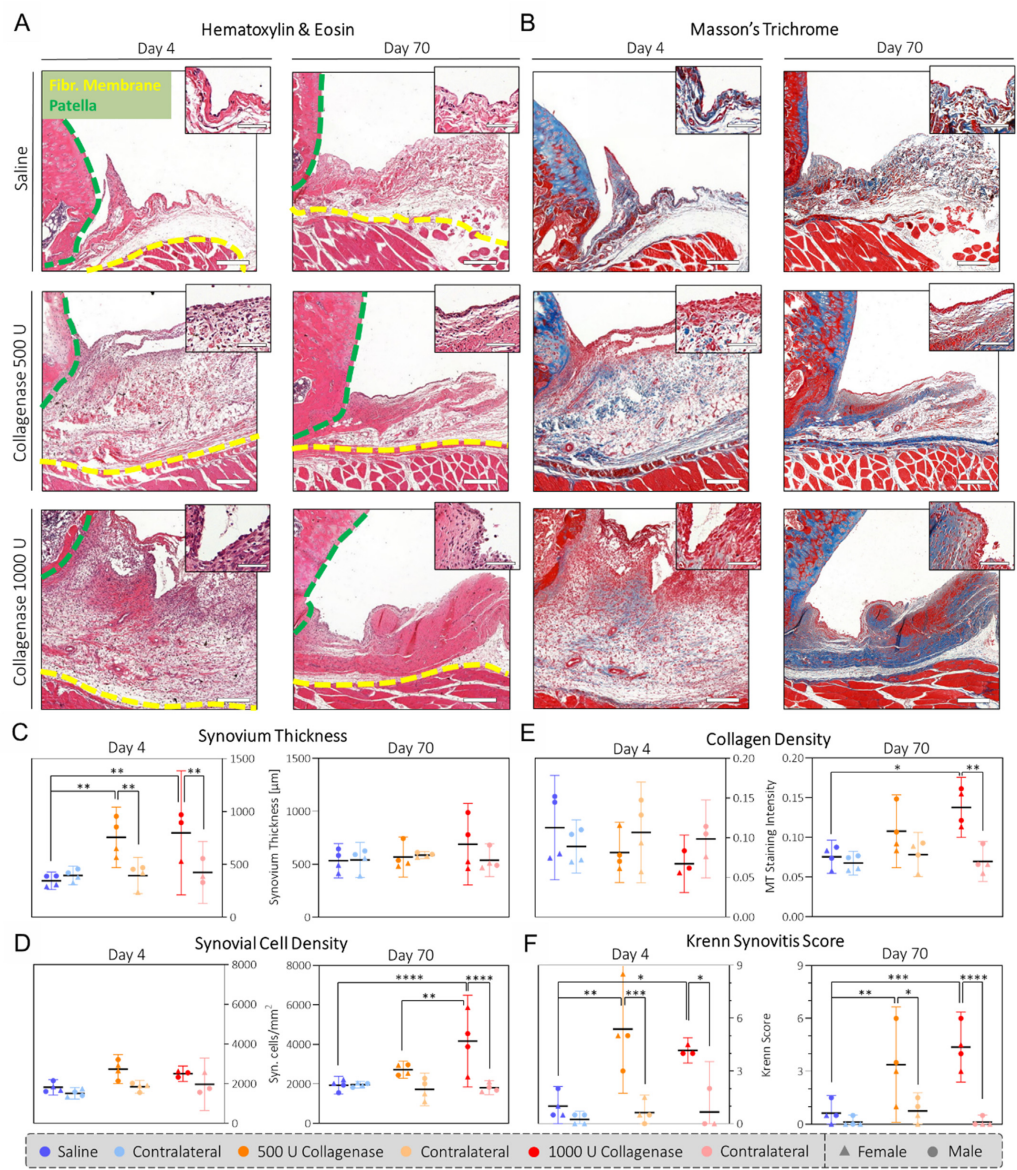
**Collagenase injection causes sustained synovitis and fibrotic remodeling of the synovium:** Representative H&E (A) and Masson's trichrome (B) staining of transverse sections of rat synovia. Scale bar: 250 ​μm, 50 ​μm (insert). Histological sections were analyzed by measuring the synovium thickness (C), synovial cell density (D), collagen density (E) and Krenn synovitis score (F). The synovium is indicated by the area between the fibrous membrane (yellow dotted line), the patella (green dotted line) and the synovial lining. N ​= ​4 (N ​= ​3 for day 4/1000 U collagenase). Full dataset available in Figs. S22–33. (For interpretation of the references to colour in this figure legend, the reader is referred to the Web version of this article.)

**Fig. 4:**
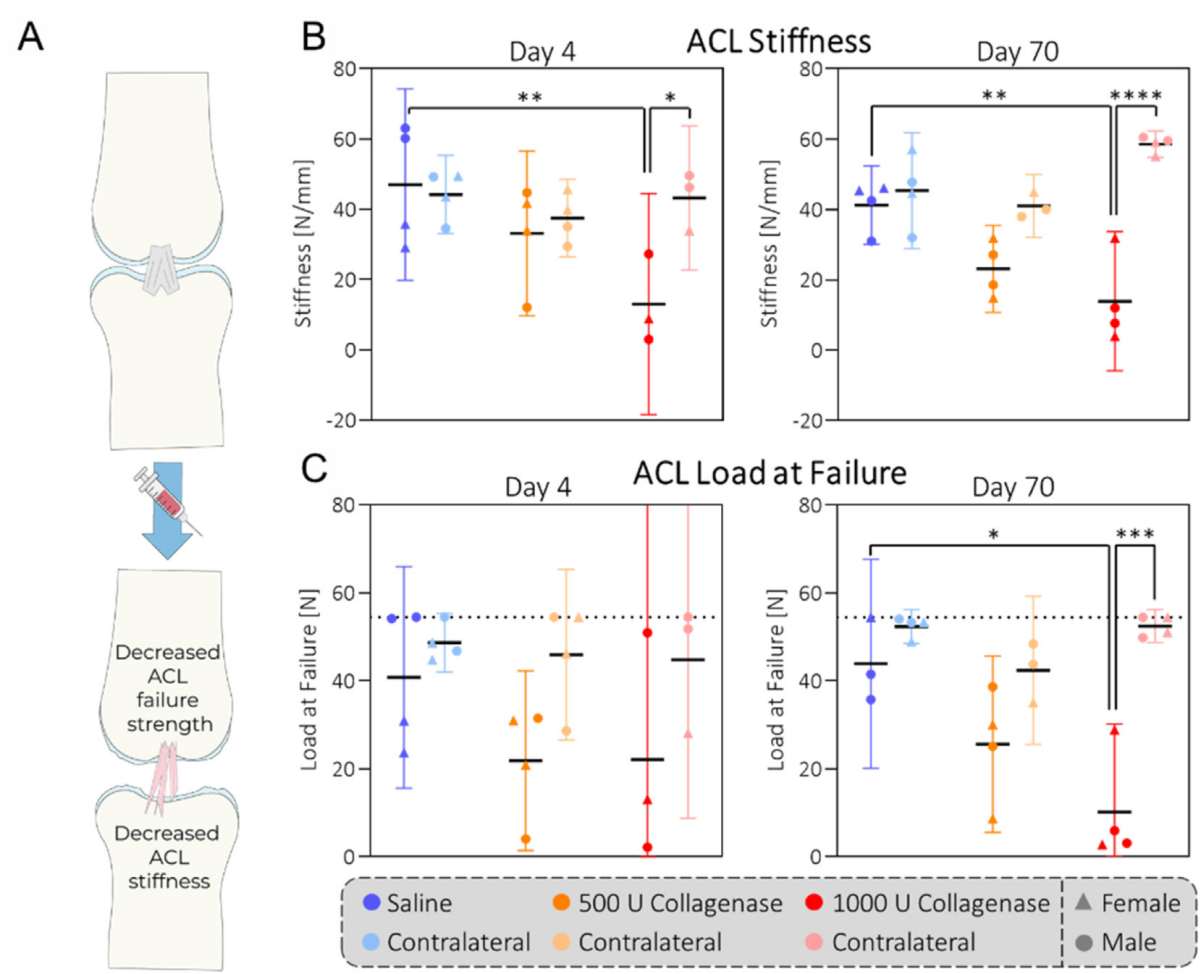
**Collagenase causes dose-related weakening of the ACL:** Tensile testing of Femur-ACL-Tibia constructs revealed decreased mechanical properties of the ACL after collagenase injection at both timepoints with respect to stiffness (B) and load at failure (C). The dotted line represents the maximal load of testing apparatus. N ​= ​4 (N ​= ​3 for day 4/1000 U collagenase).

**Fig. 5:**
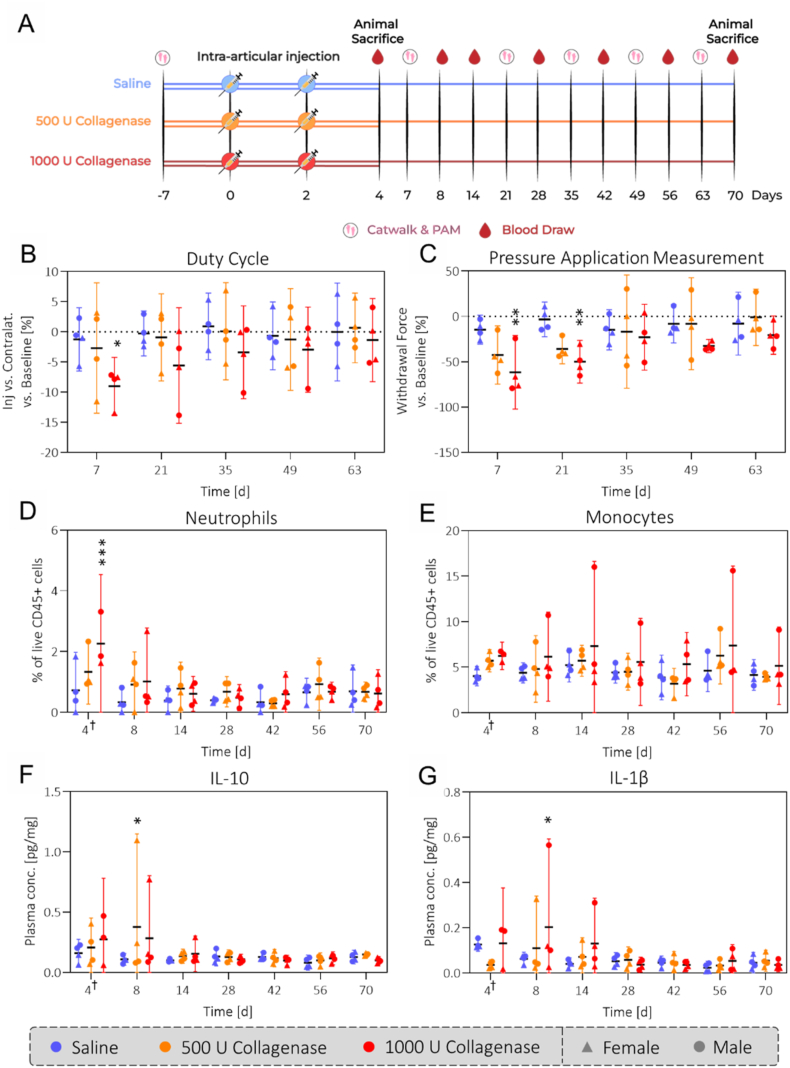
**Acute nociceptive and immune responses return to baseline levels over 10 weeks:** Both the changes in gait and mechanical hyperalgesia (B/C) as well as the systemic inflammatory response (D–G) show an acute peak for the collagenase-injected groups but return to baseline beyond day 21. Note that the duty cycle describes for how much of the step cycle the weight was resting on either of the legs (Fig. S1). The data in panel B represents the percent difference in duty cycle between injected and contralateral joints, relative to the baseline measurement on day −7. Consequently, negative values in panel B/C signify decreased weight bearing and pressure tolerance in injected joints compared to baseline. ^†^: The blood from day 4 was collected from the animals being euthanized that day. Statistical significance is indicated with respect to the saline group. N = 4 (N = 3 for day 4/1000 U collagenase).

**Fig. 6:**
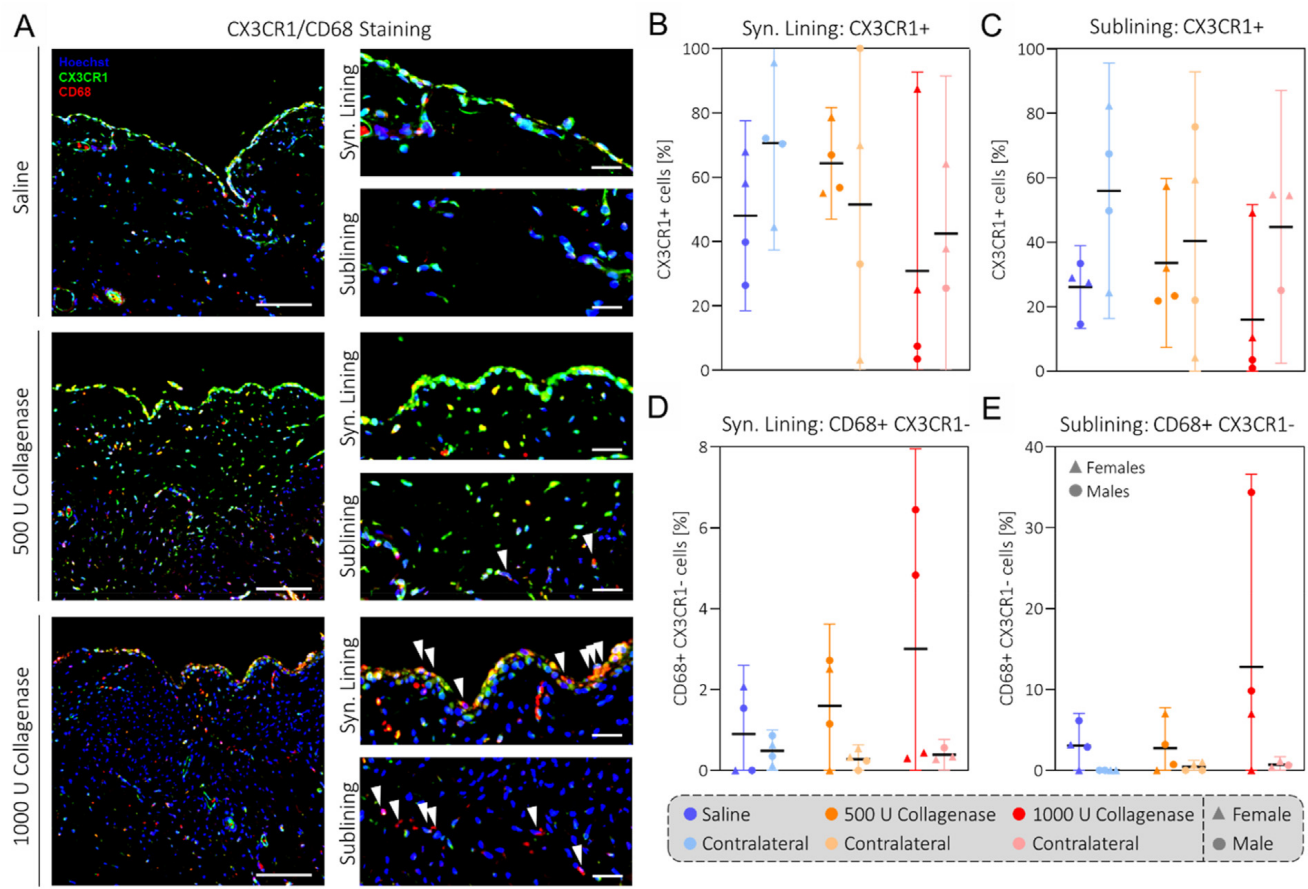
**Collagenase injection causes CX3CR1+ cell depletion and CD68** + **CX3CR1-cell infiltration in the synovium:** Images of CX3CR1 and CD68 staining of rat synovium (A) showing depletion of CX3CR1 cells in the lining and sublining of the rat 1000 U collagenase group (C/D). CD68 + CX3RCR1-cells (white arrows) are increased in the lining and sublining for the 1000 U collagenase group (D/E). Scale bars: 100 μm (left), 25 μm (right). N = 4.

**Fig. 7:**
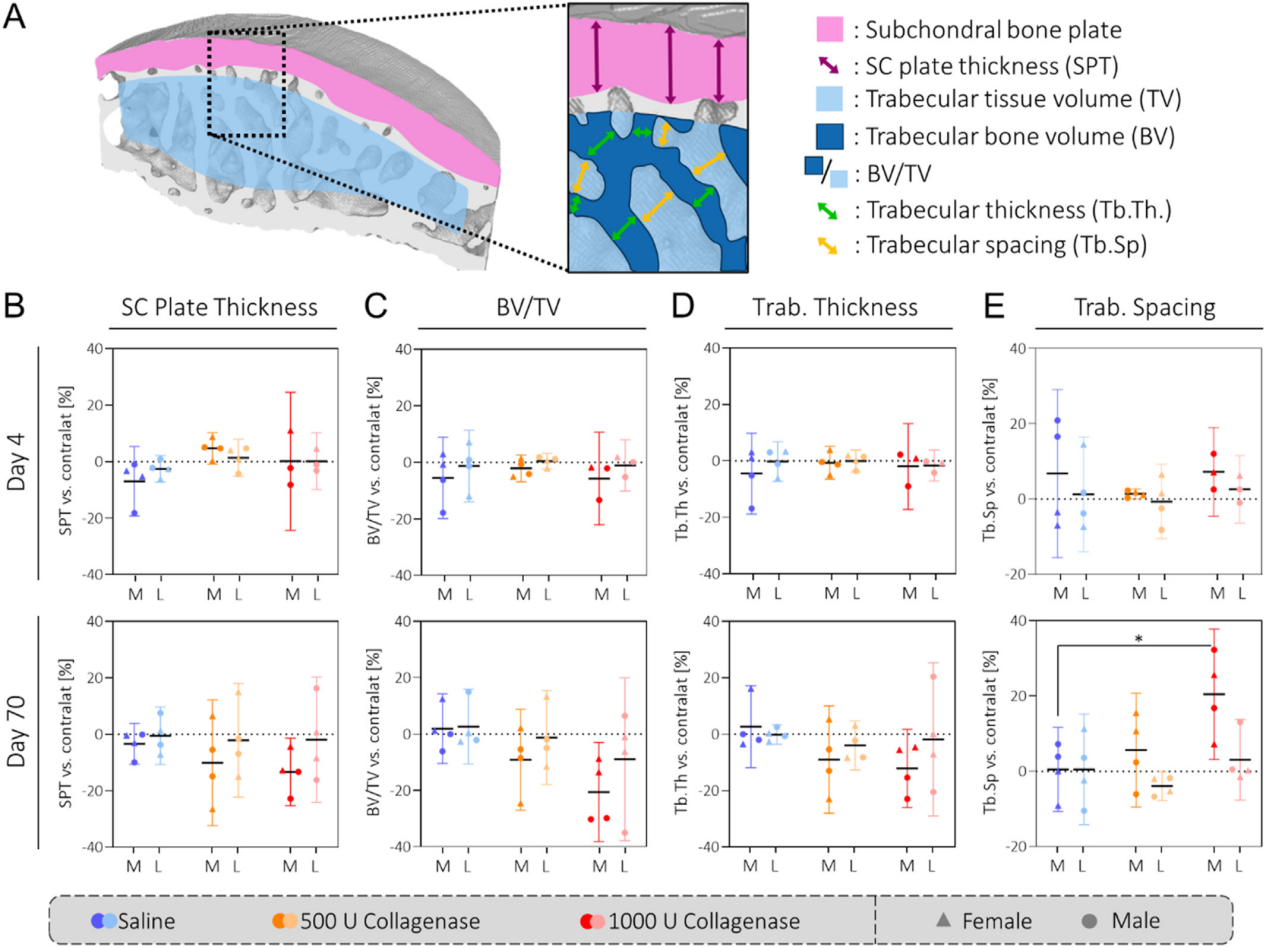
**Collagenase injection leads to bone resorption on day 70:** A) Illustration of the different bone parameters extracted from the CT scans. Note that the trabecular tissue volume was defined by a 315 ​μm offset from the subchondral bone plate surface, growth plate and condyle ROI borders respectively. The results for the medial tibia show a trend towards a dose-related decrease in subchondral bone plate thickness (B), BV/TV (C) and trabecular thickness (D) as well as an increase in trabecular spacing (E) on day 70 compared to the contralateral joint. There was no trend on day 4. M: medial, L: lateral. N ​= ​4 (N ​= ​3 for day 4/1000 U collagenase).

All statistical analysis was performed in GraphPad Prism v. 10.1.2 (GraphPad, USA). Normality was assessed with a Shapiro-Wilk test and confirmed for the majority of the collected data. One-way analysis of variance (ANOVA) was performed with a Tukey's multiple comparisons test for the data shown in Fig. 6, Fig. 7. A two-way ANOVA was conducted for Fig. 3, Fig. 4, Fig. 5 (factors: condition, timepoint) and a three-way ANOVA for Fig. 2 (factors: condition, timepoint, medial/lateral). Data was represented as mean with 95 ​% confidence intervals with significance indicated with asterisks: ∗p ​< ​0.05, ∗∗p ​< ​0.01, ∗∗∗p ​< ​0.001, ∗∗∗∗p ​< ​0.0001.

## 3 Results

### 3.1 Collagenase injection triggers dose-related cartilage degeneration

To assess cartilage degeneration, we visualized the cartilage with micro-computed tomography (μCT) using phosphotungstic acid as a contrast agent and calculated the CRS as previously described by *Kauppinen* et al. (Fig. 2A/B) [30]. This showed some small dose-related differences in CRS already on day 4 with 3.06 (95 ​% confidence interval: 2.62–3.51), 4.86 (2.99–6.73) and 6.45 (2.45–10.46) for the medial tibia for saline, 500 and 1000 U collagenase, respectively. By day 70, the medial tibia CRS increased to 3.28 (2.82–3.74), 5.40 (3.13–7.68) and 10.97 (7.02–14.92) for saline, 500 and 1000 U collagenase, respectively. The trends for the lateral tibia and the two femoral condyles were the same, though the lesions were most severe for the medial tibia. In the Safranin O staining we observed a decrease in staining intensity already on day 4, which might indicate some cartilage digestion by the injected collagenase in addition to inflammation-associated GAG release (Fig. 2C). Similarly, we observed a trend towards decreased cartilage thickness for the 1000 U collagenase group on day 4 that persisted up until day 70 (Fig. S35). Histological scoring with the OARSI cartilage degeneration score (OARSI CDS [36], Fig. 2D) revealed similar trends as for the CRS though the differences between the two timepoints were not as pronounced as for the CRS.

### 3.2 Acute synovial inflammation triggers long-term synovitis and synovial fibrosis

As synovitis is another hallmark of OA, we performed a series of semi-quantitative analyses on histologically stained synovia. Analysis of the synovia on day 4 revealed dose-independent swelling with increased synovial thicknesses of 756 ​μm (471–1042) and 798 ​μm (211–1384) and cell densities of 2727 ​cells/mm^2^ (1997–3457) and 2495 ​cells/mm^2^ (2105–2885) for 500 and 1000 U compared to 345 ​μm (262–428) and 1810 ​cells/mm^2^ (1420–2201) for saline (Fig. 3A/C/D). On day 70, the synovial thickness went back to levels comparable to the saline control. However, the 500 and 1000 U collagenase groups retained increased cellularity measuring 2721 ​cells/mm^2^ (2287–3154) and 4164 ​cells/mm^2^ (1842–6486) compared to saline with 1934 ​cells/mm^2^ (1488–2381). With Masson's trichrome staining we could relate the increased cellularity in the collagenase groups to fibrotic remodeling of the synovium with increased collagen density (Fig. 3B/E). Finally, the Krenn synovitis score indicated a more severe synovitis for the 1000 U compared to the 500 U collagenase group on day 70 (Fig. 3F, Fig. S36).

### 3.3 ACL weakening persists up to day 70

To investigate the mechanisms driving the degenerative changes we see on day 70, we assessed the mechanical properties of the ACL by subjecting femur-ACL-tibia constructs to tensile mechanical testing (Fig. 4A). On day 4, the ACL was substantially weakened with decreased stiffness values of 33.1 ​N/mm (9.7–56.6) and 13.1 ​N/mm (−18.4-44.5) for 500 and 1000 U collagenase compared to 47.1 ​N/mm (19.8–74.3) for saline (Fig. 4B). Similarly, collagenase injections caused a decrease in load at failure (Fig. 4C). The measurements on day 70 yielded very similar values, indicating persistent joint instability throughout the whole duration of our study.

### 3.4 Acute changes in gait and mechanical hyperalgesia of the joint return to baseline by day 70

Gait analysis found the duty cycle on the injected leg to be decreased for the 1000 U collagenase group on day 7 by 9.0 ​% (4.2–13.8) compared to baseline (Fig. 5B). Beyond day 7, the duty cycle returned to baseline, which also matched the trends from other gait metrics such as swing speed and stand (Fig. S37). We also assessed mechanical hyperalgesia of the joint and found decreased withdrawal thresholds for the two collagenase-injected groups on days 7 and 21, which then returned to control levels (Fig. 5C).

### 3.5 Collagenase injection causes an acute increase in peripheral neutrophils and monocytes

To investigate the role of systemic inflammation in our model, peripheral blood immune cells and inflammatory cytokines were analyzed, which indicated an acute increase in monocytes and neutrophils as well as interleukin-10 and interleukin-1 beta in collagenase-injected rats compared to saline (Fig. 5D–G, Fig. S38). Beyond day 8, we observed a normalization to saline levels.

### 3.6 Collagenase injection leads to long-term changes in cell composition of the synovium

Motivated by the acute increase in circulating immune cells, we evaluated the cellular composition of the synovia. Day 70 rat synovia were stained for CX3CR1 and CD68, the former being the marker for tissue-resident synovial-like macrophages [37] and the latter a non-lineage-specific marker for macrophages [38]. For the 1000 U collagenase group, three out of four animals showed greatly reduced CX3CR1+ cells in both synovial lining and sublining compared to saline and 500 U collagenase groups (Fig. 6A–C). Furthermore, we observed an increase in CD68 ​+ ​CX3CR1-cells in the synovial lining and sublining for the 1000 U collagenase group (Fig. 6A/D).

### 3.7 Collagenase injection leads to resorbed trabecular and subchondral bone on day 70

Following previous studies reporting bone resorption in CIOA animals [39,40], we performed subchondral and trabecular bone analysis (Fig. 7A). For the medial tibia, we found a trend towards decreased subchondral bone plate thickness and trabecular bone volume on day 70 (Fig. 7B–E). For the medial femoral condyle, we observed a similar, albeit weaker trend and on the lateral side there was no trend for either of the bones (Fig. 7B–E, Fig. S39). On day 4, there were no trends at all (Fig. 7B–E, Fig. S39). We also investigated the presence of osteophytes following the OARSI histopathology initiative [36] and found no indication of osteophytes in any of the samples (Figs. S10–21).

### 3.8 No sex differences in any of the data

Despite sex being one of the main risk factors in human OA, the differences between male and female animals in our study were minimal (Figs. S40–42). The only statistically significant difference was observed for the synovial thickness where there was a greater increase in males compared to females on day 4. However, this does not connect to any of the other synovitis metrics at either timepoints and should be taken with caution due to the low animal number in this study.

### 3.9 Adverse effects for 1000 U collagenase group

During the week of injections, there was an expected decrease in animal weight for all groups (Fig. S43) due to increased handling and repeated analgesia. This was particularly pronounced for animals of the 1000 U collagenase group, in which two animals reached almost 10 ​% weight loss on day 9 before starting to regain weight.

Another consideration for the 1000 U collagenase condition is that one animal was removed from the study on day 2 due to an anterolateral tibial dislocation of the injected knee joint. Furthermore, signs of extraarticular hemorrhage were found in this group upon dissection of the joints on day 4 that were not, however, found on day 70.

## 4 Discussion

Our aim was to characterize the CIOA model in rats and provide novel insights into the roles of mechanical degradation and inflammation in this model. As the animal number in this study was low, some of the datasets suffered from large intra-group variability which makes it difficult to draw any definitive conclusions. Nevertheless, we found several strong trends that might provide inspiration to other researchers working with the CIOA model. We found that the intra-articular injection of collagenase reproducibly weakens the ACL in a dose-related manner (Fig. 4). Despite the ACL not being completely disrupted, the total level of induced joint destabilization might compare to the anterior cruciate ligament transection model, as the collagenase is expected to also weaken all other intracapsular ligaments. Previously, the stability of the rat ACL has only been reported in a collagen-induced model for rheumatoid arthritis, where the severe local inflammation triggered a decrease in stiffness by around 40 ​% compared to the control [41], which is in range of our 500 U collagenase group. In our case, however, the degree of instability remained unchanged throughout the experiment (Fig. 4), indicating greatly reduced inflammation compared to the rheumatoid arthritis model, as expected [[42], [43], [44]].

Nevertheless, inflammation does play a key role in the model. Analysis of blood biomarkers in the collagenase-injected animals showed that levels of plasma cytokines, monocytes and neutrophils were elevated systemically on similar time scales as the changes in gait and mechanical hyperalgesia (Fig. 5). This systemic response was likely caused by damage-associated molecular patterns and chemotactic proteins produced by the cells in the joint as a response to collagenase exposure. Though systemic levels of immune cells returned to baseline after 8 days, synovitis persisted until day 70 (Fig. 3E/F). As monocytes are precursors of macrophages, the elevated levels of CD68 ​+ ​CXC3R1-cells in the synovium of the 1000 U collagenase animals can be explained by infiltration from the periphery (Fig. 6D/E). The observation that the acute gait asymmetry and increased mechanical hyperalgesia returned to baseline in the long-term indicates a relatively mild pain phenotype in this model which is in accordance with the findings from other related small and large animal OA models [27,45,46].

Regarding the changes in the subchondral and trabecular bone, our results confirm previous reports of bone resorption in the CIOA model [39,40]. Furthermore, the fact that the changes were most pronounced in the medial tibia where cartilage degeneration was also the most substantial suggests some cartilage-bone crosstalk. While human late-stage OA is typically associated with an increase in bone volume, bone resorption has been implicated as a feature of the early stages of the disease and several animal studies have reported a switch to increased bone volume at the later timepoints [11,47,48]. Moreover, a comparison of our rat synovia with human OA tissues revealed similarities with respect to the levels of CX3CR1+ cells as well as the number of synovial lining cells (Fig. S44A/C). However, the increase of CD68 ​+ ​CX3CR1-cells in the rat synovial sublining was not as substantial in the human samples, nor were the levels of fibrosis (Fig. S44B/C). On the cartilage level, the CRS calculated for the human tissues was greatly increased compared to both collagenase groups (Fig. S44D). Together with the finding that no changes in gait nor mechanical hyperalgesia were observed on day 70, this indicates both collagenase groups as relatively mild models of OA.

When comparing the results for the two different collagenase doses, we find that while both groups show clear signs of synovitis on day 70 (Fig. 3), the levels of cartilage degeneration for the 500 U collagenase group are quite low compared to the high-dose group (Fig. 2). Considering the acute inflammatory response after collagenase injection for both doses, this model generally mimics the case of post-traumatic OA. While the developed phenotype for the 500 U collagenase dose after 70 days represents the early stages of OA development with barely any cartilage degeneration, the degenerative changes in the 1000 U collagenase group are more advanced.

Mechanistically, the finding that synovitis levels of the collagenase groups on day 4 were dose-independent but then followed a different trajectory until day 70, which strongly indicates crosstalk between the tissues (Fig. 3). As the level of joint instability was related to the collagenase dose throughout the study, this certainly was a contributing factor (Fig. 4) and it is well established that altered joint biomechanics can induce synovitis [49,50]. Another contributing factor might have been the increase in circulating monocytes and neutrophils that can be associated with the dose-specific infiltration of CD68 ​+ ​CX3CR1-cells into the synovium (Fig. 5, Fig. 6).

## 5 Conclusion

Our study provides a holistic overview of the CIOA model with a particular focus on inflammatory and mechanical factors. We show that the dose-related changes in the synovium on day 70 are likely a consequence of mechanical and systemic factors. In addition to the mechanistic interplay between inflammation and mechanical degradation, the CIOA model mirrors other hallmarks of human OA such as cartilage degeneration, increased synovial lining cells, synovial fibrosis and bone remodeling. By changing the injected collagenase dose, we have good control over the severity of the model, with both 500 and 1000 U groups still, however, developing a mild OA-like phenotype. If a more severe phenotype is desired, we recommend extending the duration of the experiment, as substantial adverse effects can be expected for doses >1000U. Even for 1000 U, the elevated cartilage degeneration levels on day 4 question the relevance of this dose in the context of OA. The main limitation of this specific study is the low animal number. However, our study presents several quantitative methods, such as the CRS or the semi-automated analysis of synovial histology, to complement histological scoring and increase both the standardization and the statistical power of future studies. Furthermore, we want to urge the use of skeletally mature animals of both sexes and the use of activity-controlled collagenase to enable better comparability between studies. In conclusion, we hope that this study can inspire the use of the CIOA model in a more hypothesis-driven, clinically relevant way in the future.

## Author contributions

PW, KB, DF and MZW contributed to the conception and design of the study. PW, DF, MA and MZW were responsible for the animal work. PW, KB and DF were responsible for the collection and analysis of the experimental data. SK and TF developed the methodology for and performed the CRS and bone analysis calculations. PW, KB, SZ, MF, LB and TZ were involved in the histological grading of the tissue sections. PW and KB wrote the draft of the article. All authors contributed to the interpretation of the results, critical revision of the article and have given their final approval of the article.

## Data availability statement

P. Weber, K. Bevc et al., https://doi.org/10.3929/ethz-b-000668851, ETH Zurich Research Collection 2024.

## Role of the funding source

This work was supported by the Swiss National Science Foundation (Grant No. 315230_192656 to MZW) and the European Union's HORIZON EUROPE 2021–2027 Research and Innovation Actions (Grant No. 101095084).

## Declaration of competing interest

The authors declare that they have no known competing financial interests or personal relationships that could have appeared to influence the work reported in this paper.
