# Supplementary material for: The collagenase-induced osteoarthritis (CIOA) model: Where mechanical damage meets inflammation
Source: Osteoarthr Cartil Open. 2024 Oct 24;6(4):100539. doi: 10.1016/j.ocarto.2024.100539 (PMC11584605; doi:10.1016/j.ocarto.2024.100539)
Supplement: Multimedia component 1 [file mmc1.pdf]

Supplementary Information

Collagenase-Induced Osteoarthritis Model in Rats: Where Mechanical Damage Meets Synovitis

Patrick Weber[1,†], Kajetana Bevc[1, †], David Fercher[1], Sami Kauppinen[2], Shipin Zhang[1], Maryam Asadikorayem[1], Lucia Baixauli Marin[2], Tianqi Zhang[2], Tuomas Frondelius[2], Gian Salzmann[3], Valentino Bruhin[3], Jakob Hax [3,4], Gonçalo Barreto[5], Mikko A.J. Finnilä[2,6], Marcy Zenobi-Wong[1*]

[†]equal contribution

[1]Tissue Engineering + Biofabrication Laboratory, Department of Health Sciences and Technology, ETH Zürich, Otto-Stern-Weg 7, 8093 Zürich, Switzerland

[2]Research Unit of Health Sciences and Technology, University of Oulu, Aapistie 5A, 90220, Oulu, Finland

[3]Schulthess Klinik, Department for Knee Surgery, Lengghalde 2, 8008 Zürich

[4]Faculty of Medicine, University of Zurich, Pestalozzistrasse 3, 8032 Zurich, Switzerland

[5]Clinicum, Faculty of Medicine, University of Helsinki and Helsinki University Hospital, Haartmaninkatu 8, 00290, Helsinki, Finland

[6]Biocenter Oulu, University of Oulu, Aapistie 5A, 90220, Oulu, Finland

Corresponding Author E-mail: marcy.zenobi@hest.ethz.ch

Supplementary Methods

1. Animal Experimentation

1.1 Housing

Animals were housed in groups of 3 in individually ventilated cages. Due to their increased size, males were housed in larger cages (NexGen RAT 1800, Allentown Inc, USA, 1800 cm^2) than females (1500U SEAL SAFE, Tecniplast, AUS, 1500 cm^2). Paper tissues and gnawing wood were provided as enrichment to promote animal wellbeing and movement in the cage.

1.2 Group Size Calculation

The sample size was calculated using the pwr.anova.test() function in R. The effect size was estimated as large (f=0.4) following *Cohen et al*. For 6 experimental groups (3 conditions x 2 timepoints with a significance level of 0.15 and a power of 0.5, the resulting group size was calculated at N=4.90.

1.3 Randomization

The average weight of the animals from day -12, -10 and -7 was used to assign them to the different experimental groups, minimizing the weight difference as well as ensuring the presence of one animal per group in each cage. The cages were then randomly assigned to the timepoints and the side of injection was randomly determined for each animal using the sample() function in R with two restrictions: 1) The same amount of female and male animals at each timepoint. 2) Same number of left- and right-side injections for each sex and group combination.

1.4 Blinding

Researchers were blinded throughout the study and analysis of the data and the different groups was only disclosed after conclusion of the study. Animal numbers were used as unique identifiers during the tissue characterization.

2. Human samples

For reference to our rat tissues, human synovium and cartilage samples from end-stage OA patients (Table S4) undergoing total knee arthroplasty were collected in accordance with ethical agreement (2021-01836) after the patients signed the informed consent. The samples were fixed in 4% paraformaldehyde and prepared for histology.

3 Histology Staining Protocols

3.1 Hematoxylin & Eosin Staining

Rehydrated tissue sections were stained with Mayer's hematoxylin for 8 min, washed with water, followed by 95% ethanol, and counterstained in 0.5% Eosin Y solution (in acidified ethanol) for 45 seconds. After rinsing in 100% EtOH, the sections were dehydrated in Xylene (2x 1 min each), and coverslipped using Eukitt mounting medium.

3.2 Masson's Trichrome Staining

Rehydrated tissue sections were stained with the trichrome stain kit (Abcam, UK) following manufacturer instructions.

3.3 Safranin O

After µCT analysis, rat femur and tibia were decalcified in aqueous 10% NH_4-EDTA solution and prepared for histology analogously as above. 5 µm anterior and posterior coronal sections were prepared from the weight bearing area with a spacing of 200 µm. Rehydrated sections were stained with 0.5% Safranin O for 16 minutes, washed with water for 10 minutes, dehydrated to xylene, coverslipped and scanned on an Aperio AT2 (Leica Biosystems, GER).

3.4 Immunofluorescent Stainings

Rehydrated human and rat synovium tissue sections were cooked in sodium citrate buffer at 60 °C for 60 minutes for antigen retrieval. They were subsequently blocked with 5% BSA for 1 hour and stained with primary antibodies for CX3CR1 (14-6093-81, Invitrogen, USA, 1:200, 5 µg/mL for rat & 1:100, 10 µg/mL for human tissues), CD68 (#375602, BioLegend, USA, 1:100, 5 µg/mL for human tissues) and CD68 (#31630, Abcam, UK, 1:200, 5 µg/mL for rat tissues). All primary antibodies were pre-validated and pre-titrated and diluted in 1% BSA solution. Subsequently, tissue sections were stained with secondary antibodies anti-mouse AF568 (A-11004, Invitrogen, USA, 1:500, 4 µg/mL for all tissues), anti-goat AF488 (A-11055, Invitrogen, USA, 1:500, 4 µg/mL for all tissues) and anti-rabbit AF647 (A-21244, Invitrogen, USA, 1:500, 4 µg/mL for all tissues) and Hoechst 33342 (Thermo Fisher Scientific, USA, 1:1000). Secondary antibodies and Hoechst were also diluted in 1% BSA. Stained sections were coverslipped with Anti-Fade Fluorescence Mounting Medium (ab104135, Abcam, UK) and scanned on an Olympus SlideView VS200 device (Olympus, JPN).

4. Histology Quantification Macros

The automated histological quantification on the H&E stained synovia was run on Fiji ImageJ v1.51n using the macros below. Prior to the analysis, the ROI was measured and the images cropped such that the results could be normalized to the ROI area.

4.1 Cell Density Quantification

```
input = "C:/ ";
list = getFileList(input);

setBatchMode(true);
for (i = 0; i < list.length; i++){
        open(input + list[i]);
        selectWindow(list[i]);
   run("Colour Deconvolution", "vectors=[H&E 2]");
        selectWindow(list[i]+"-(Colour_2)");
        close;
        selectWindow(list[i]+"-(Colour_3)");
        close;
        selectWindow(list[i]);
        close;

   selectWindow(list[i]+"-(Colour_1)");
   run("16-bit");

        setAutoThreshold("Default dark");
        run("Threshold...");
        setThreshold(0, 104);
        run("Convert to Mask");
        run("Close");

        run("Analyze Particles...", "size=1-Infinity show=[Bare Outlines] display summarize");
```

```
        saveAs("PNG", input + list[i] + "_processed.png");
        close;
        selectWindow(list[i]+"-(Colour_1)");
        close;
}
setBatchMode(false);
```

4.2 Masson's Trichrome Staining Intensity

```
input = getDirectory("Choose Source Directory ");
        output = getDirectory("Choose Destination Directory ");

        list = getFileList(input);
setBatchMode(true);
for (i = 0; i < list.length; i++){
        open(input + list[i]);
        selectWindow(list[i]);
   run("Colour Deconvolution", "vectors=[User values] hide [r1]=0.0999715853 [g1]=0.73738605
[b1]=0.6680326 [r2]=0.7995107 [g2]=0.5913521 [b2]=0.10528667 [r3]=0.59227383
[g3]=0.3264422 [b3]=0.7366459");
   selectWindow(list[i]+"-(Colour_2)");
        run("Measure");
        close;
        selectWindow(list[i]+"-(Colour_1)");
        close;
        selectWindow(list[i]+"-(Colour_3)");
        close;
        selectWindow(list[i]);
        close;
}
        selectWindow("Results");
        saveAs("Results", output+"Results.csv");
        setBatchMode(false);
```

Table S1: Available literature on the collagenase dose used in other rat studies with the observed phenotype

Author	Dose per injection	Observed phenotype
Adães[1]	250, 500 U	Day 42, Recovered knee swelling, persistent nociceptive changes, cartilage degradation, osteophyte formation, synovitis
Adães[2]	500 U	Day 42: Recovered knee swelling, persistent nociceptive changes, cartilage degradation, osteophyte formation, synovitis, altered expression of neuronal injury markers in the dorsal root ganglion
Jeong[3]	1500 U	Day 19: Increased pro-inflammatory cytokines in serum, cartilage degradation
Nirmal[4]	50 U	Day 34: Synovitis, cartilage fibrillation, increased GAG levels in serum, swelling of paw and knee, paw edema
Nirmal[5]	50 U	Day 34: Synovitis, cartilage fibrillation, increased GAG levels in serum, swelling of paw and knee, paw edema, increased MMP expression in synovium
Shivnath[6]	50 U	Day 30: Weight loss, increased serum alkaline phosphatase, cartilage degradation
Won[7]	1500 U	Day 18: Increased mechanical allodynia

Enzyme	Activity
Collagenase	290 U/mg
Caseinase	535 U/mg
Clostripain	4.70 U/mg
Trypsin	0.58 U/mg
FALGPA	0.14 U/mg

Table S2 – Collagenase Enzymatic Activities: Low-activity batch of collagenase with the following enzymatic activities as measured by Stemcell Technologies. Enzymatic activity was documented using the FALGPA assay in-house prior to injection into the animals.

Marker	Fluorophore	Clone	Provider	LOT
**Granulocyte marker**	FITC	HIS48	Invitrogen #11-0570-82	2472711
**CD3**	BV605	1F4	BD Horizon #563949	2139001
**CD4**	V450	OX-35	BD Horizon #561579	1277819
**CD8a**	PerCP eFluor710	OX8	Invitrogen #46-0084-82	2527293
**CD43**	PE	W3/13	BioLegend #202812	B318984
**CD45R**	PECy7	HIS24	Invitrogen #25-460-82	2410870
**CD45**	AF700	OX-1	BioLegend #202218	B377155
**CD161**	APC	3.2.3	BioLegend #205606	B367998
**CD32**	-	D34-485	BD Pharmingen #550270	2094087

Table S3 – Flow Cytometry Antibodies: List of the antibodies used for the PBMC flow cytometry characterization. Note that the CD32 antibody was used to reduce unspecific staining. A Zombie NIR^{TM} fixable viability kit (#423105c, BioLegend, USA) was furthermore used to identify viable cells.

Disease	Sex		Average Age
	M	F	
End-stage OA	3	7	64,3 +/- 8,06

Table S4 – Human OA synovia donor information

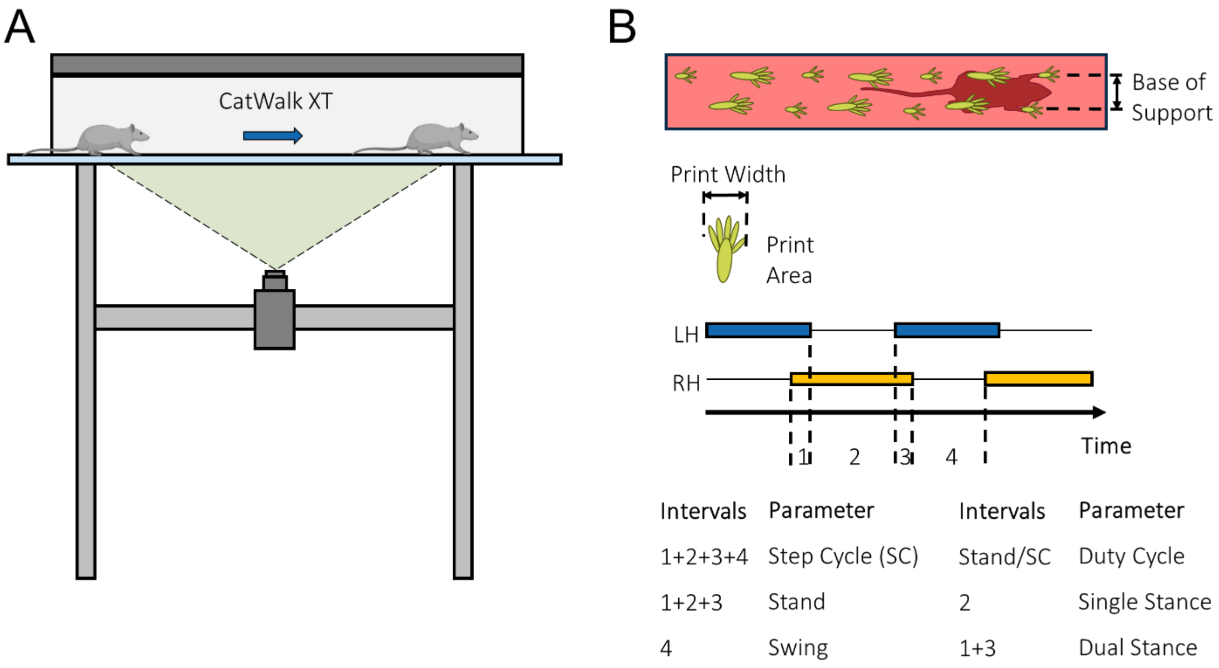

Figure S1 – CatWalk measurements: A) Schematic illustration of the experimental setup for the CatWalk measurements. Each animal in the study ran across the stage three times with the footprints being captured by a camera from below. B) Schematic illustration of the calculated gait metrics that were used for the analysis. Figure created with BioRender.

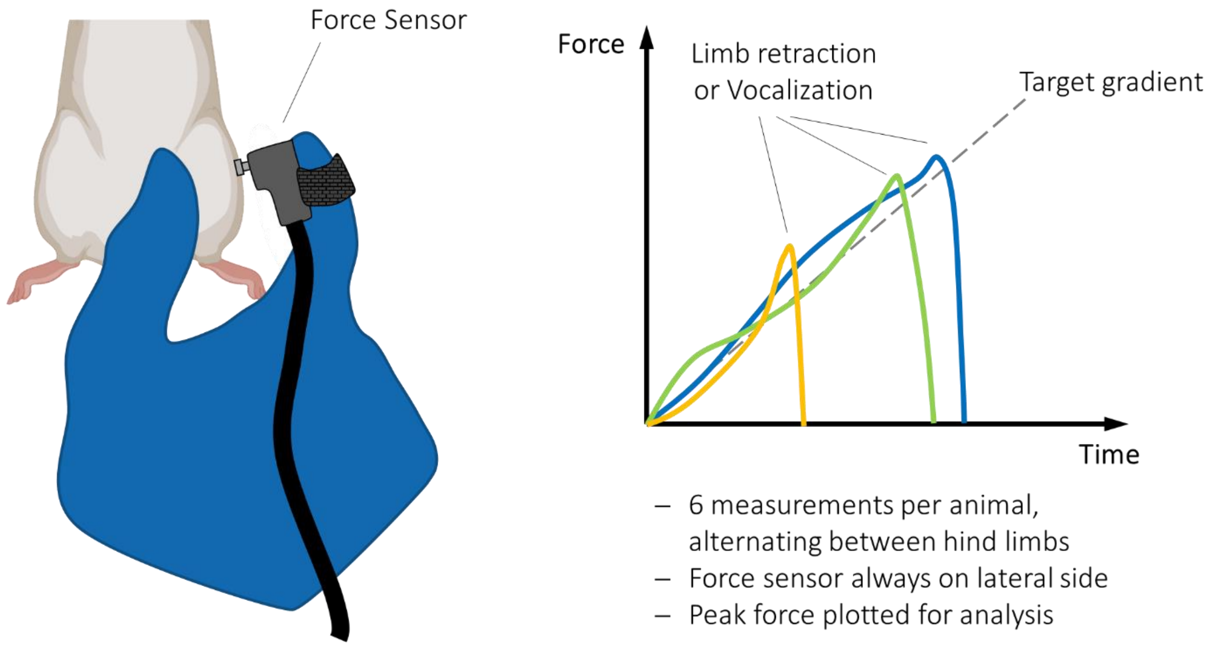

Figure S2 – Pressure Application Measurement: Schematic illustration of the PAM setup. Hind knee joints were clamped in medio-lateral orientation between thumb and index finger with the pressure sensor on the lateral side. The applied pressure was linearly increased until the limb was retracted or the animal vocalized. The average peak force of 3 measurements per limb was used for the analysis. Figure created with BioRender.

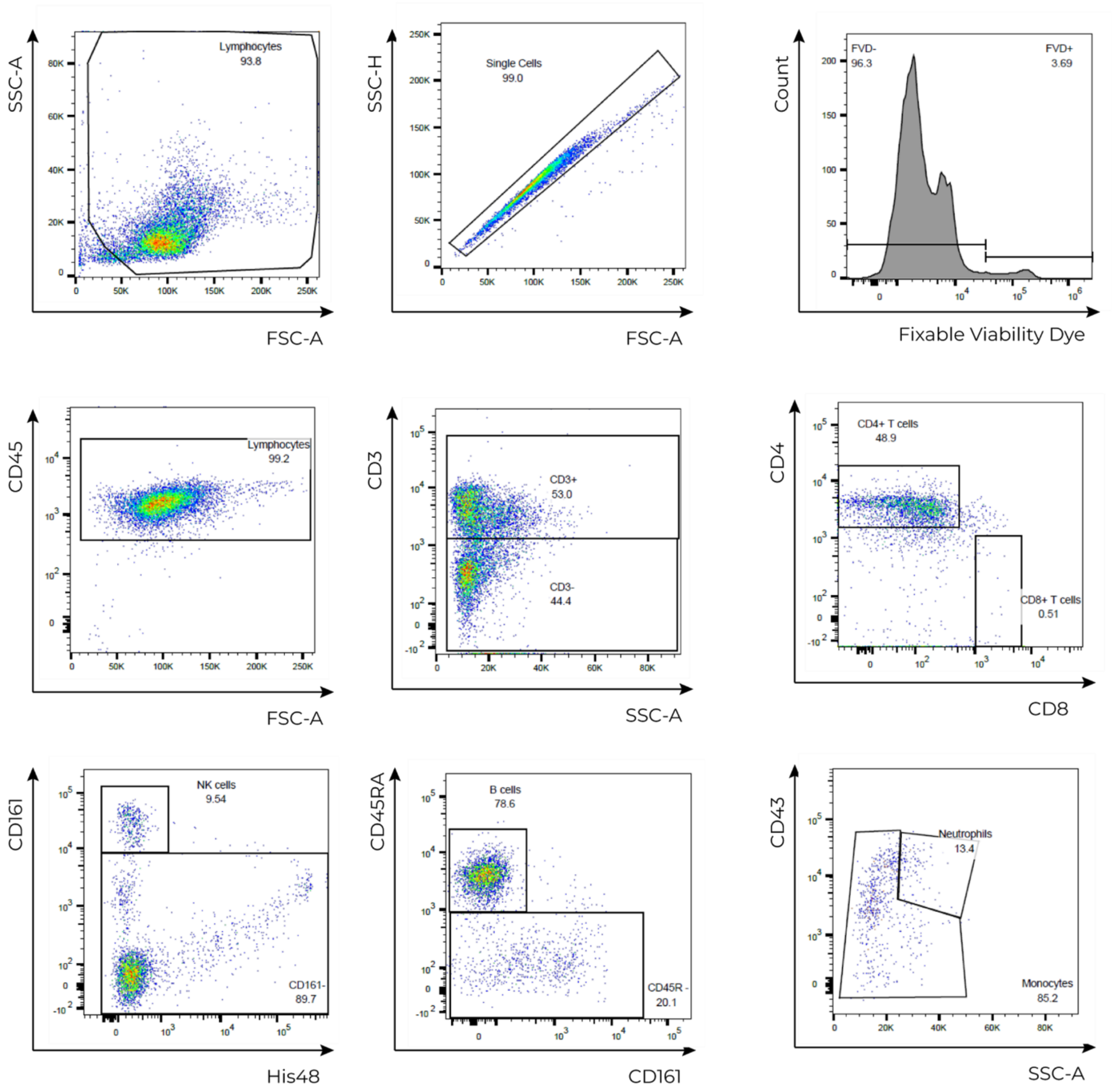

Figure S3 – Flow Cytometry Gaiting Strategy: FlowJo 10 gating of the peripheral blood immune cells according to the selected markers (markers specified on axes, corresponding cell names specified next to the gates), percentages correspond to this selected example of a 1000 U collagenase-injected female rat on day 8.

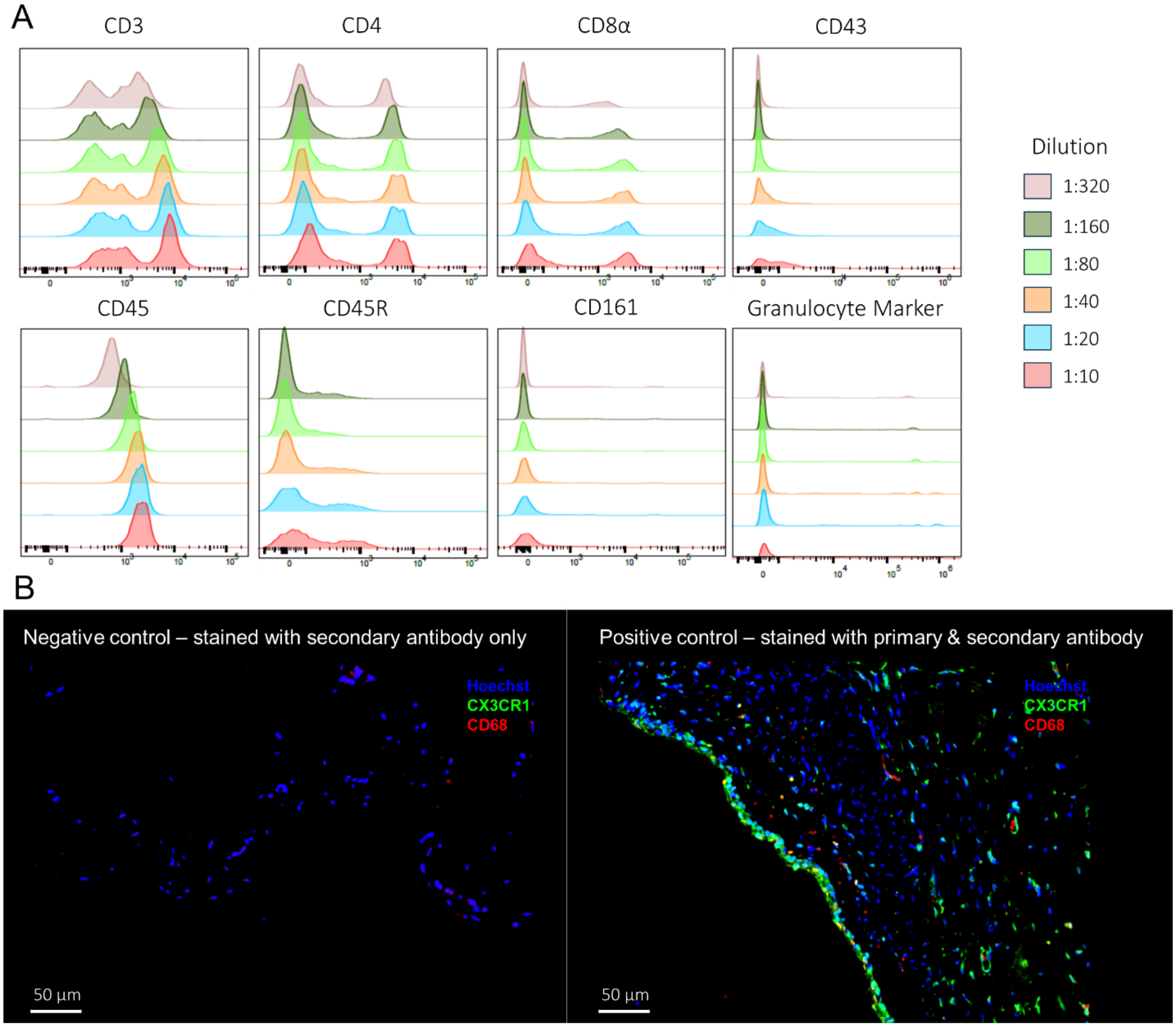

Figure S4 – Antibody titration and validation: A) Population histograms of rat blood immune cells stained with a single antibody of an increasing dilution, made with FlowJo 10. B) Positive and negative control immunofluorescence staining of rat synovium to validate antibody specificity.

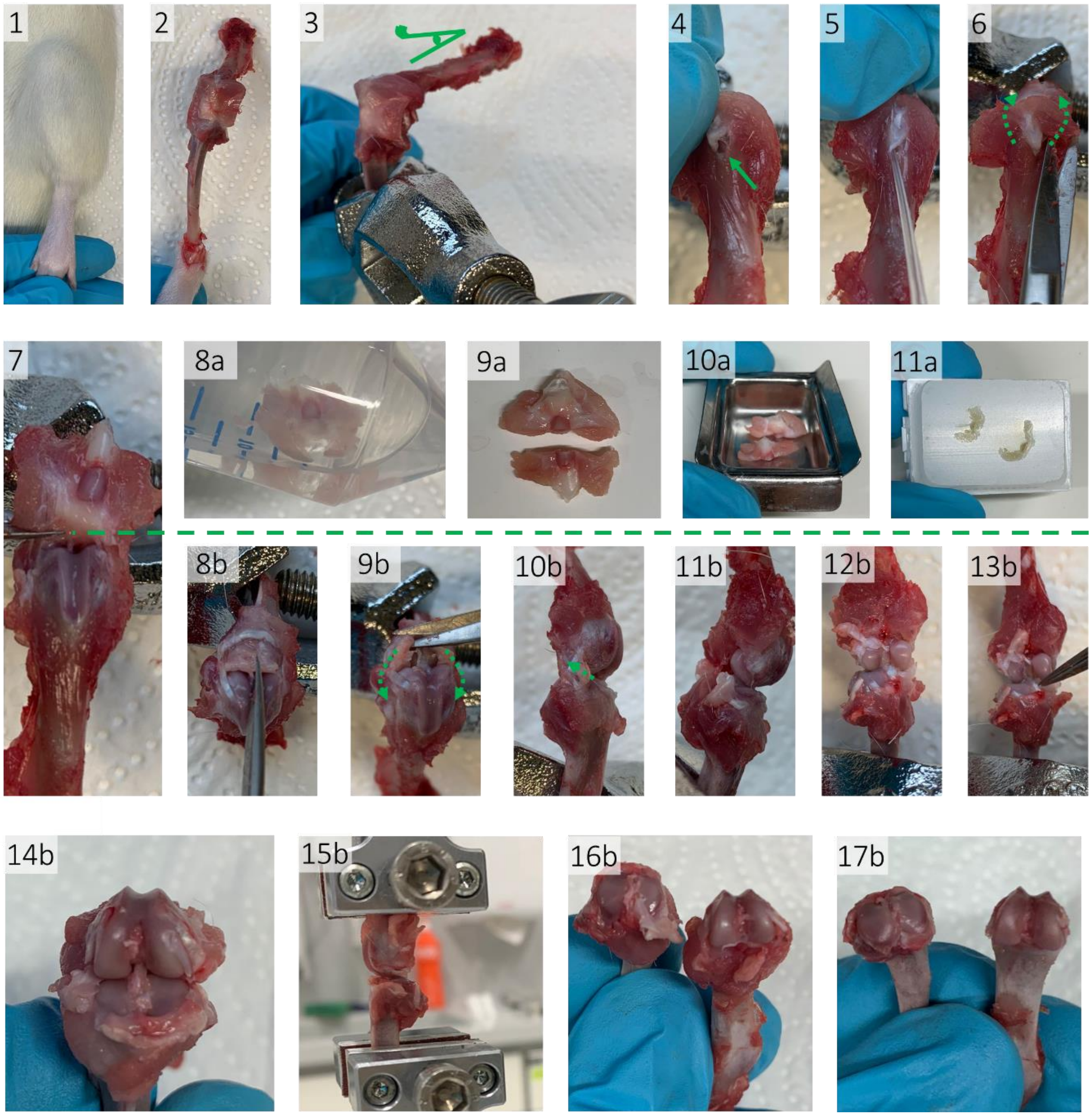

Figure S5 – Step-by-step illustration of joint tissue harvest:

1) Intact rat knee joint

2) Leg removed by dislocation of hip joint (to prevent cracks for tensile testing). Capsule intact, all other tissues removed.

3) Paw removed and tibia clamped for fixation. Sample turned to have back view on joint capsule for step 4.

4) Incision made proximally to the patella, above the trochlear groove.

5) Synovial fluid harvested with extended length pipette tips.

6) Joint capsule opened by cutting around the patella.

7) Image of open joint capsule. Peripatellar joint capsule cut away from tibia.

8a) Peripatellar joint capsule fixed and decalcified.

9a) Peripatellar joint capsule halved in transverse direction through patella.

10a) Tissues paraffinized and embedded with cut plane facing downwards.

11a) Histology blocks with peripatellar joint capsule cross-section.

8b) Under full flexion, anterior meniscal ligaments transsected.

9b) Menisci cut away from tibia.

10b) Under full extension, medial collateral ligament transsected. Posterior joint capsule cut.

11b) Image of exposed medial femoral condyle.

12b) Repeat step 10 on lateral side to expose both condyles.

13b) Posterior cruciate ligament cut.

14b) Image of femur-ACL-tibia construct showing intact anterior cruciate ligament.

15b) Construct mounted at full extension for tensile testing.

16b) Femur and tibia separated after ACL rupture.

17b) Remove soft tissue to have cartilage fully exposed for subsequent μCT analysis.

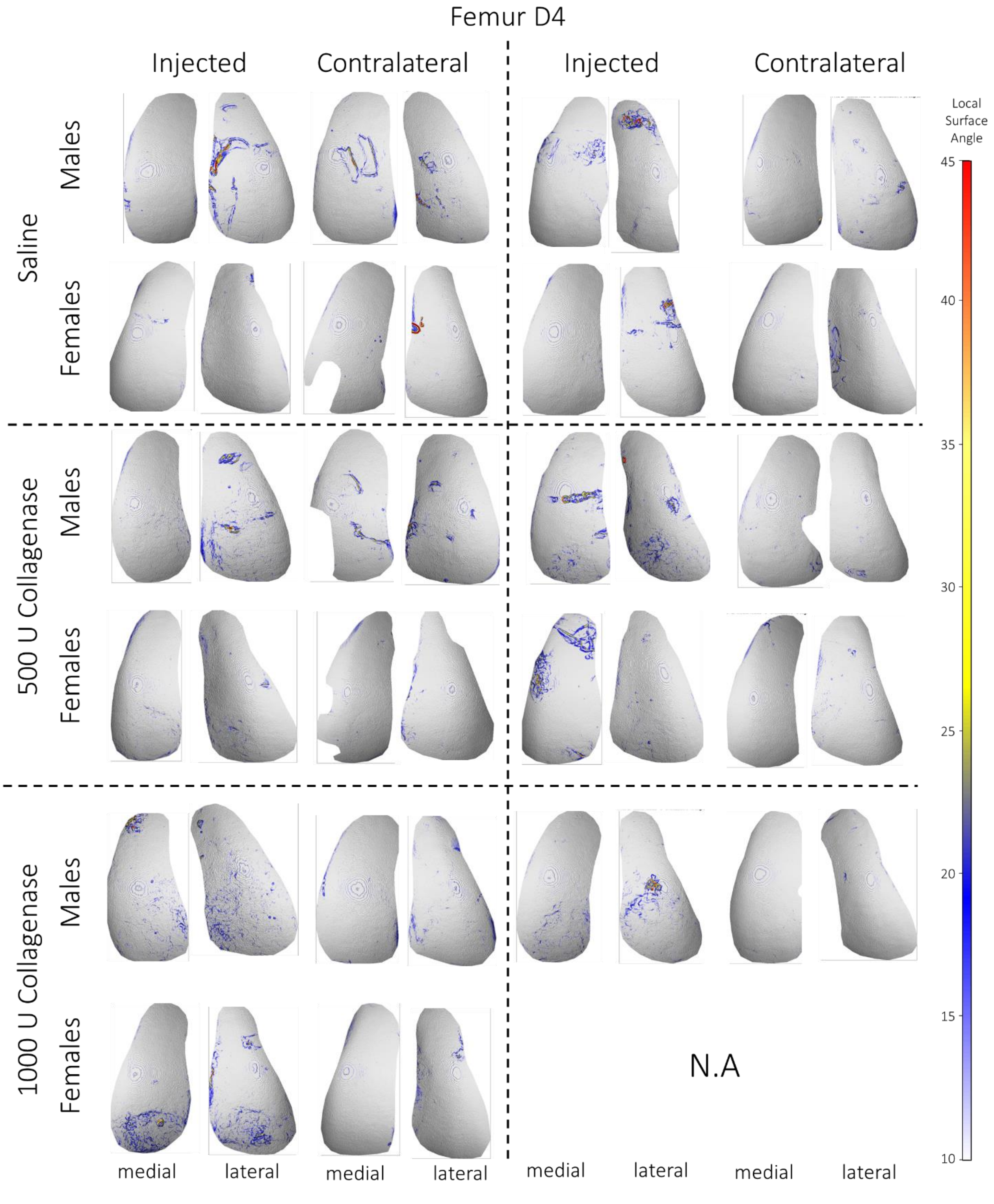

Figure S6: Complete CRS roughness maps of all the femurs in this study on day 4. Note that the second female of the 1000 U collagenase group needed to be removed from the study due to a anterolateral tibial dislocation of the injected knee joint.

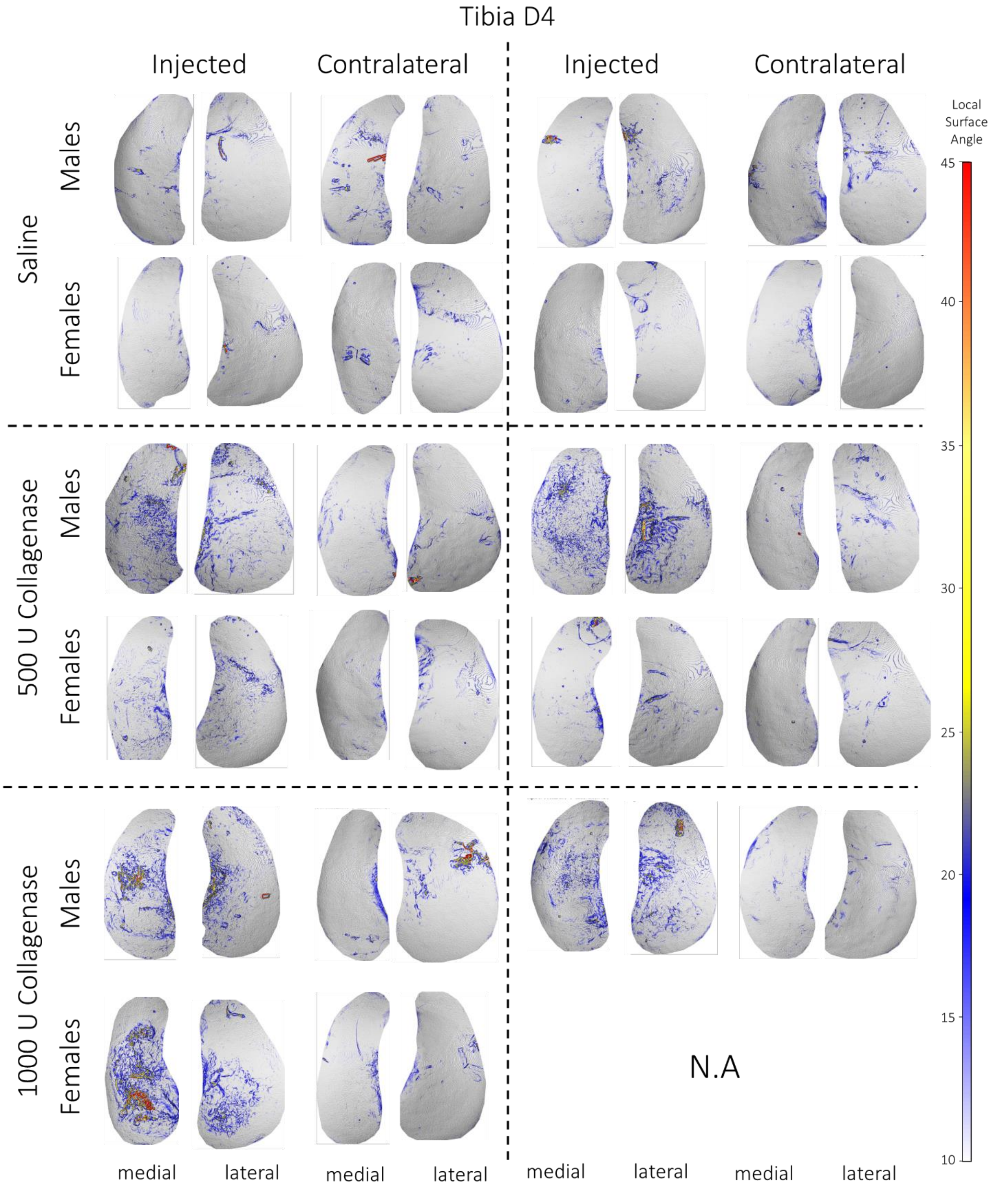

Figure S7: Complete CRS roughness maps of all the tibias in this study on day 4. Note that the second female of the 1000 U collagenase group needed to be removed from the study due to a anterolateral tibial dislocation of the injected knee joint.

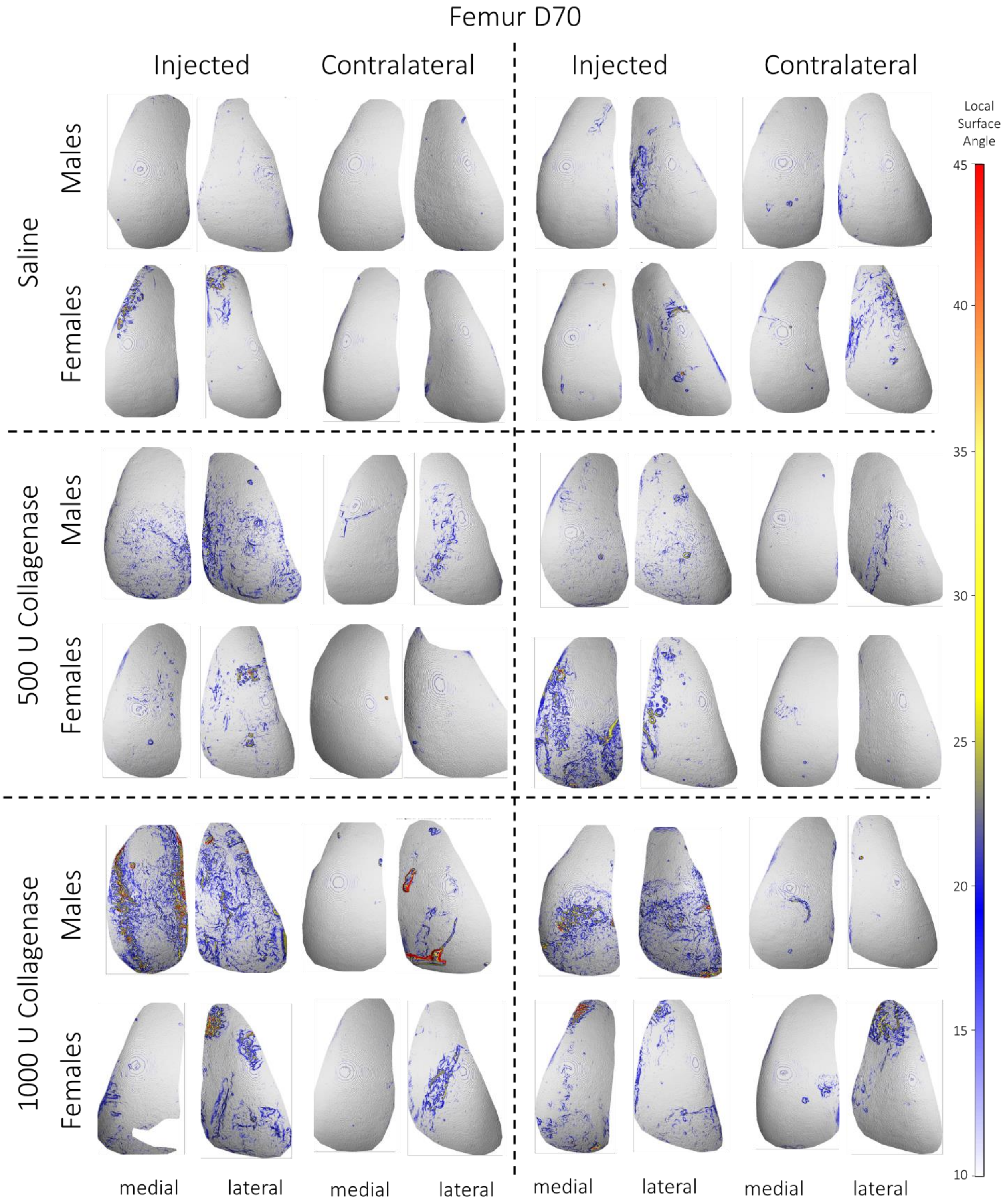

Figure S8: Complete CRS roughness maps of all the femurs in this study on day 70.

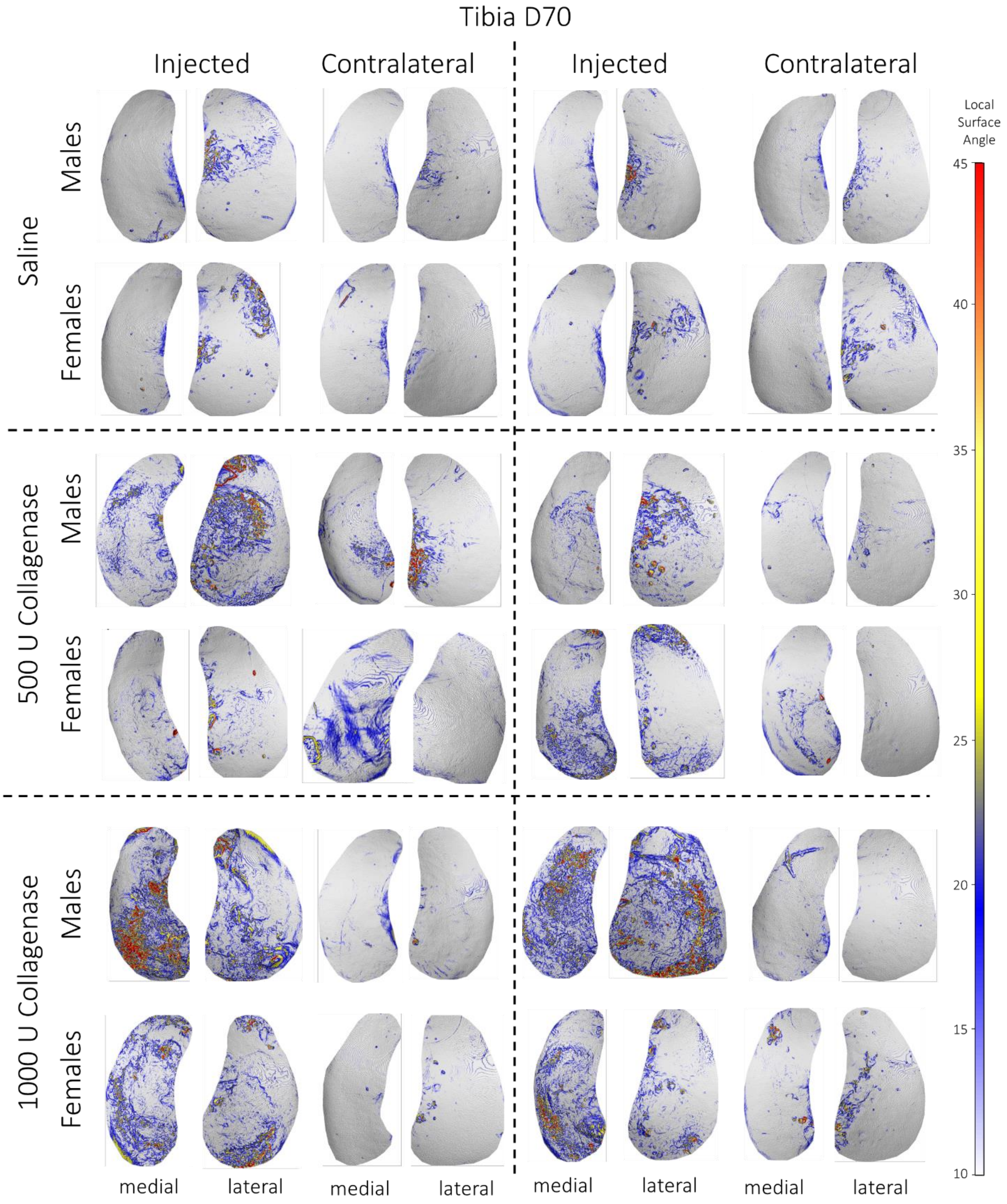

Figure S9: Complete CRS roughness maps of all the tibias in this study on day 70.

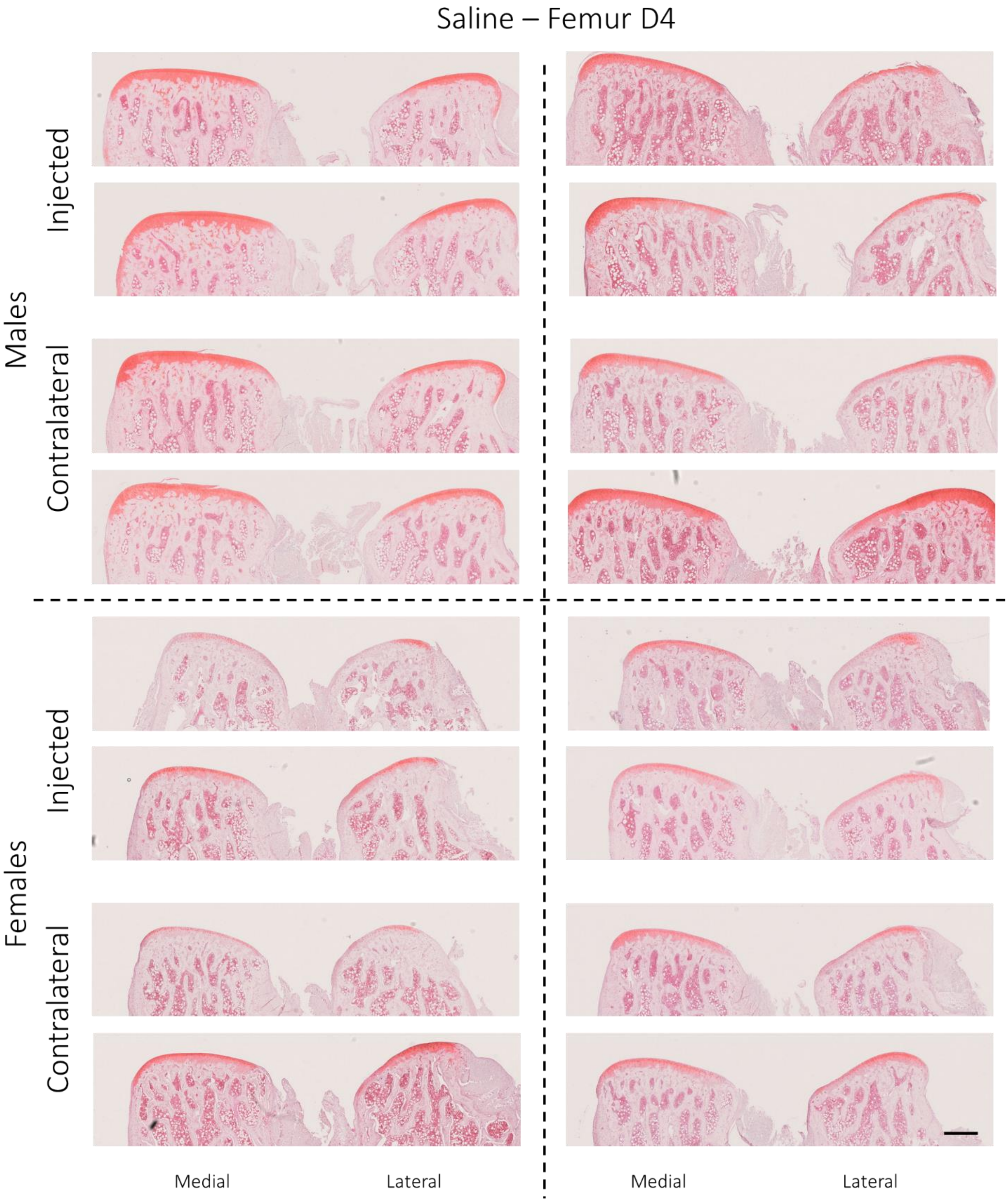

Figure S10: Complete safranin O histology of all the femurs in the saline group on day 4. There are two sections per joint. Scale bar: 500 μm.

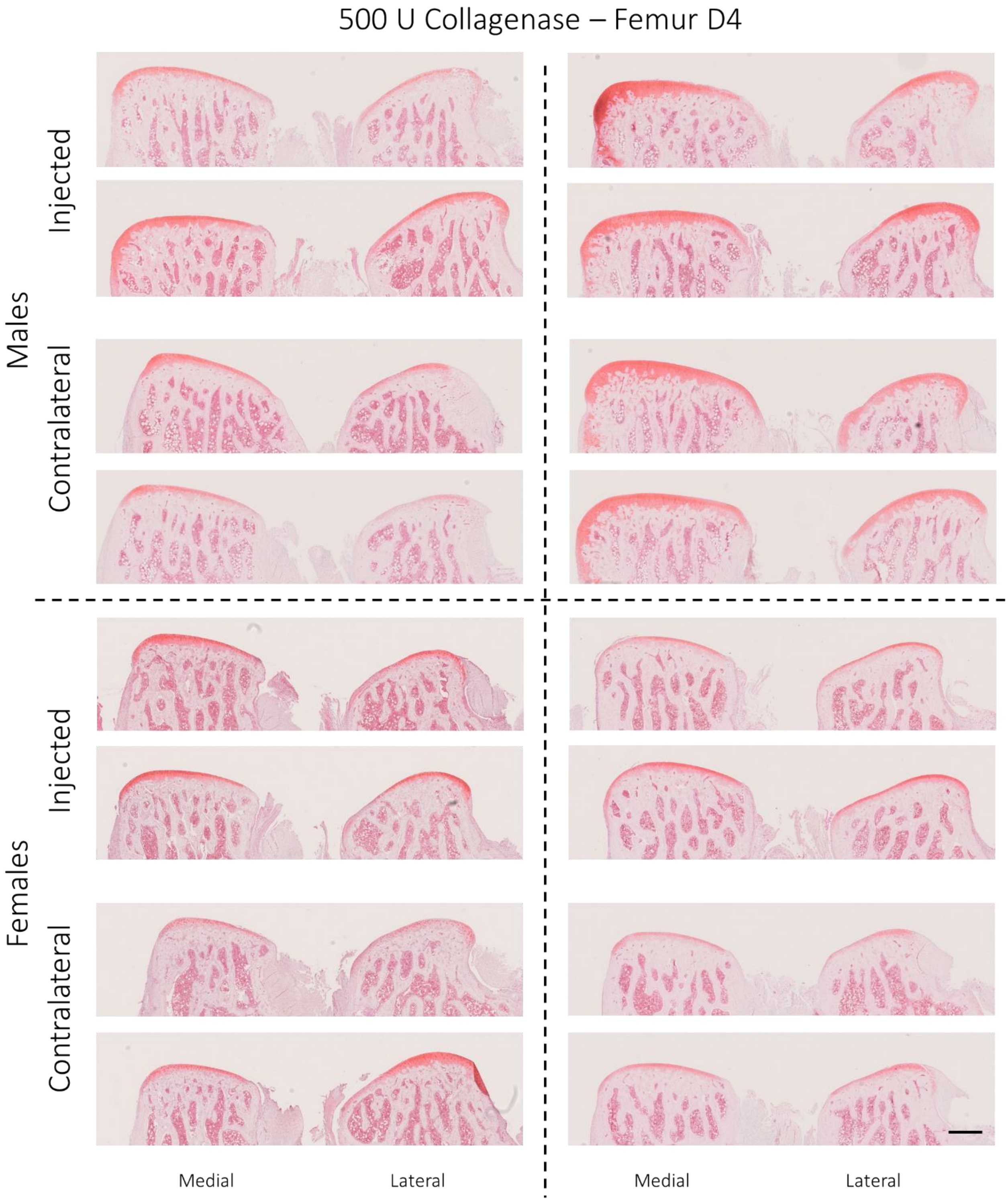

Figure S11: Complete safranin O histology of all the femurs in the 500 U collagenase group on day 4. There are two sections per joint. Scale bar: 500 μm.

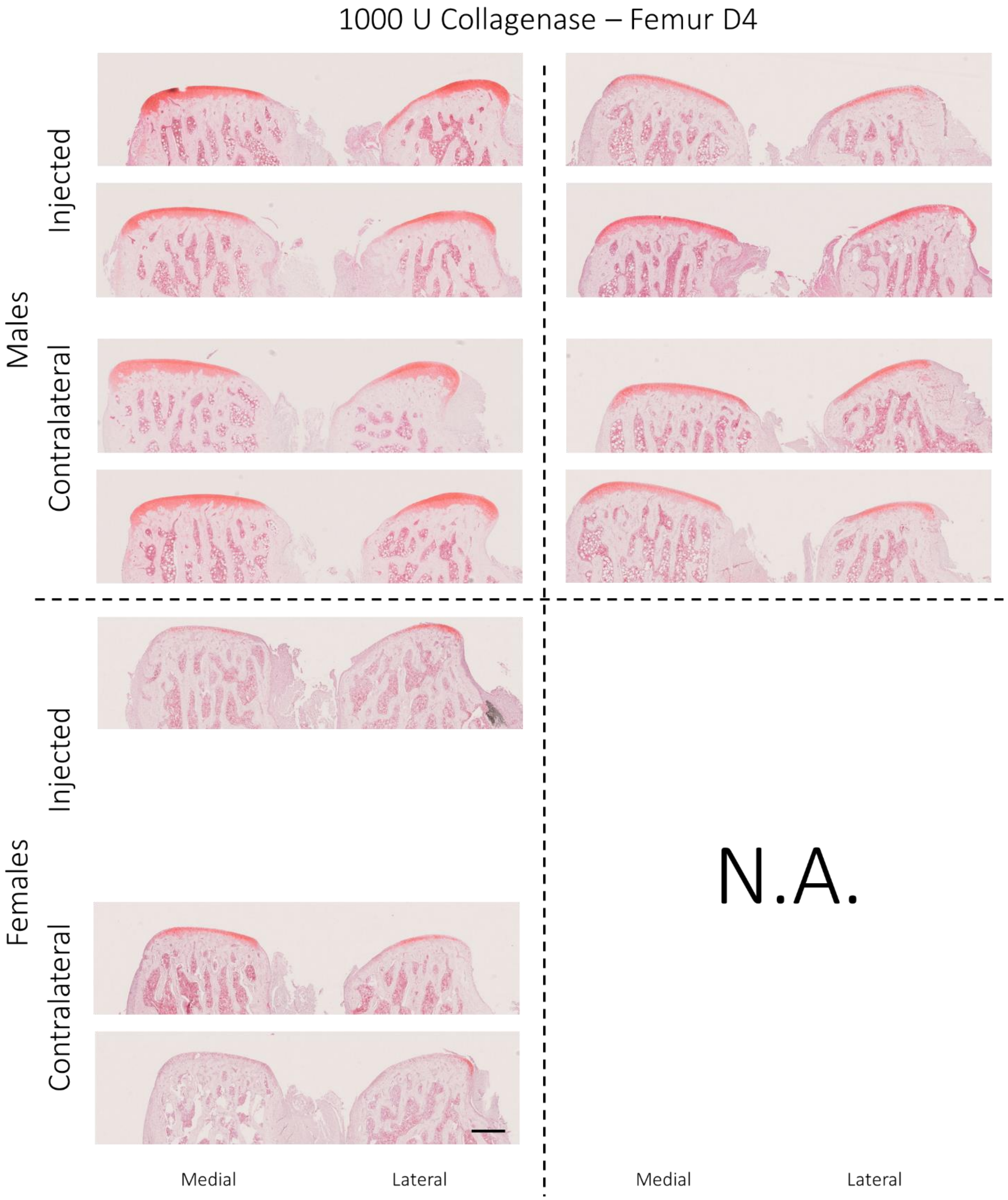

Figure S12: Complete safranin O histology of all the femurs in the 1000 U collagenase group on day 4. There are two sections per joint. Note that the second female needed to be removed from the study due to a anterolateral tibial dislocation of the injected knee joint. Scale bar: 500 μm.

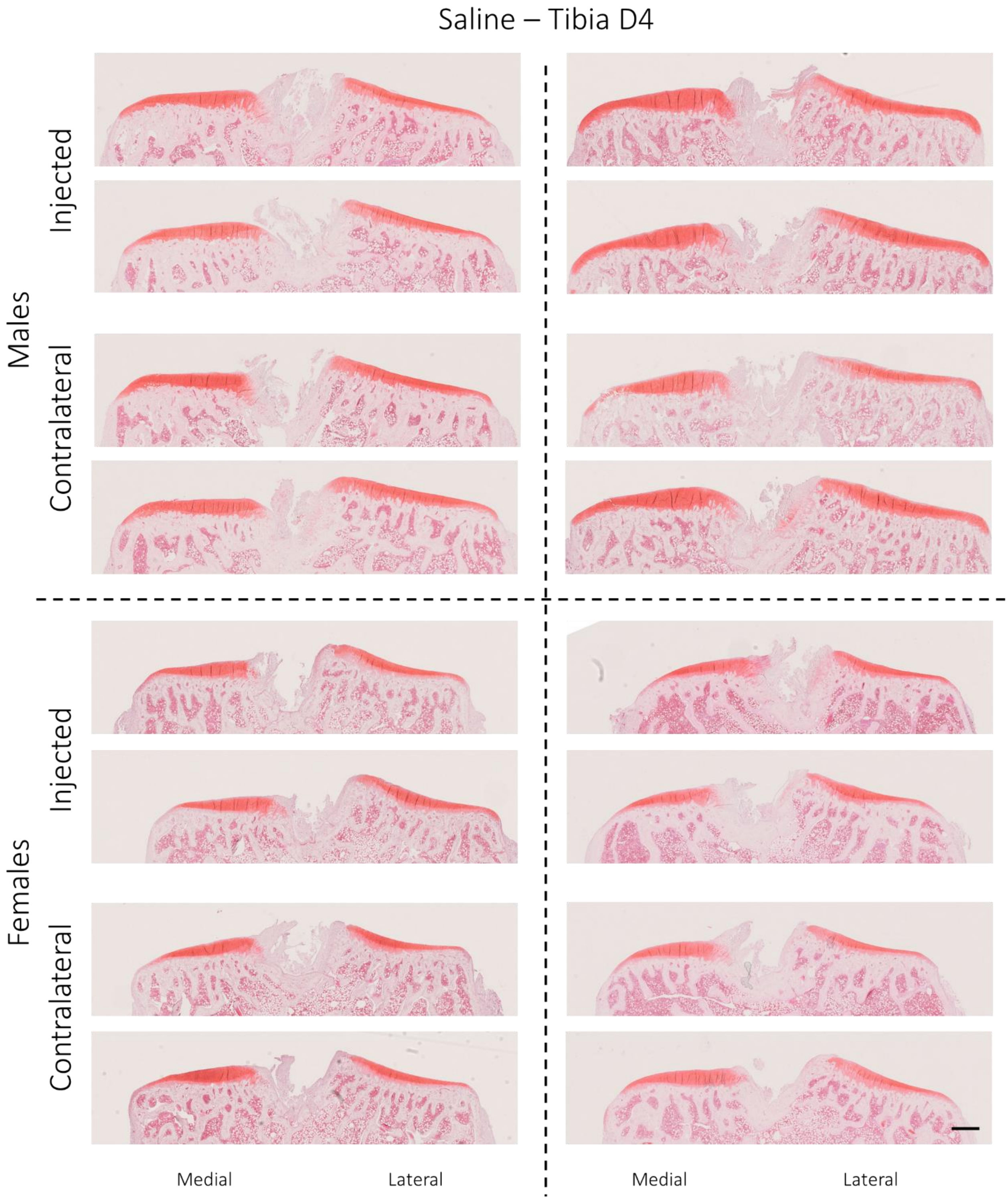

Figure S13: Complete safranin O histology of all the tibias in the saline group on day 4. There are two sections per joint. Scale bar: 500 µm

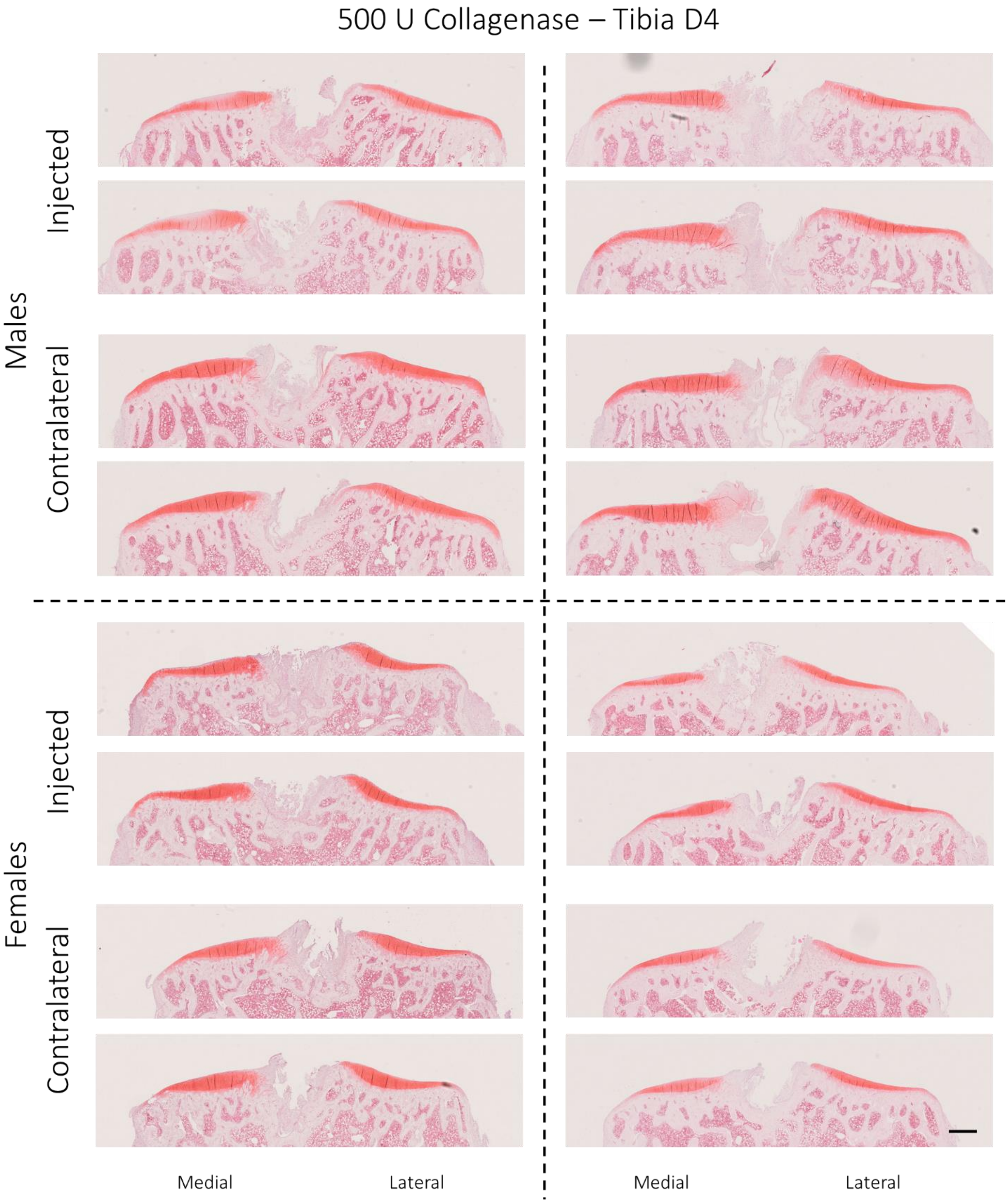

Figure S14: Complete safranin O histology of all the tibias in the 500 U collagenase group on day 4. There are two sections per joint. Scale bar: 500 µm

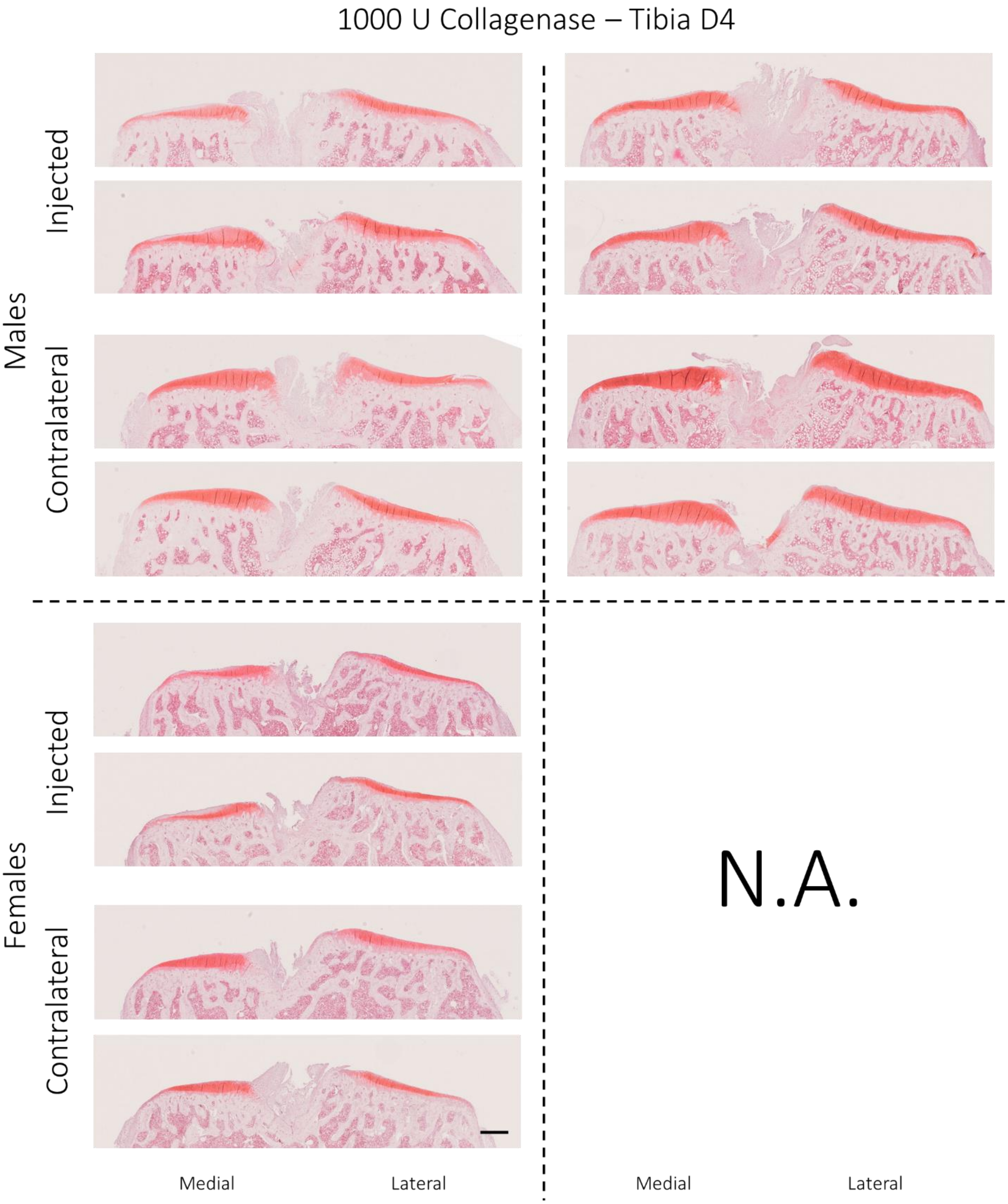

Figure S15: Complete safranin O histology of all the tibias in the 1000 U collagenase group on day 4. There are two sections per joint. Note that the second female needed to be removed from the study due to a anterolateral tibial dislocation of the injected knee joint. Scale bar: 500 μm

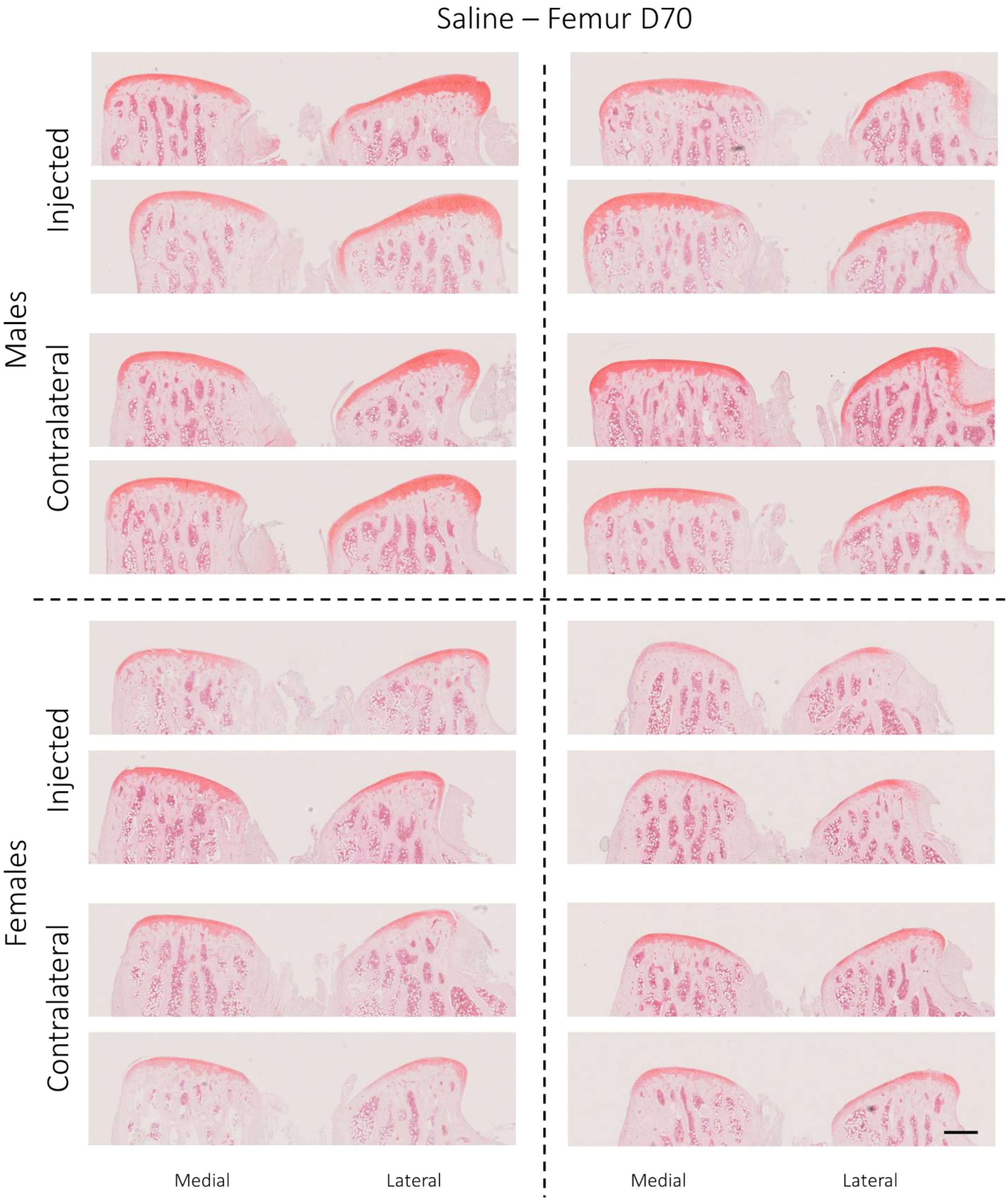

Figure S16: Complete safranin O histology of all the femurs in the saline group on day 70. There are two sections per joint. Scale bar: 500 µm

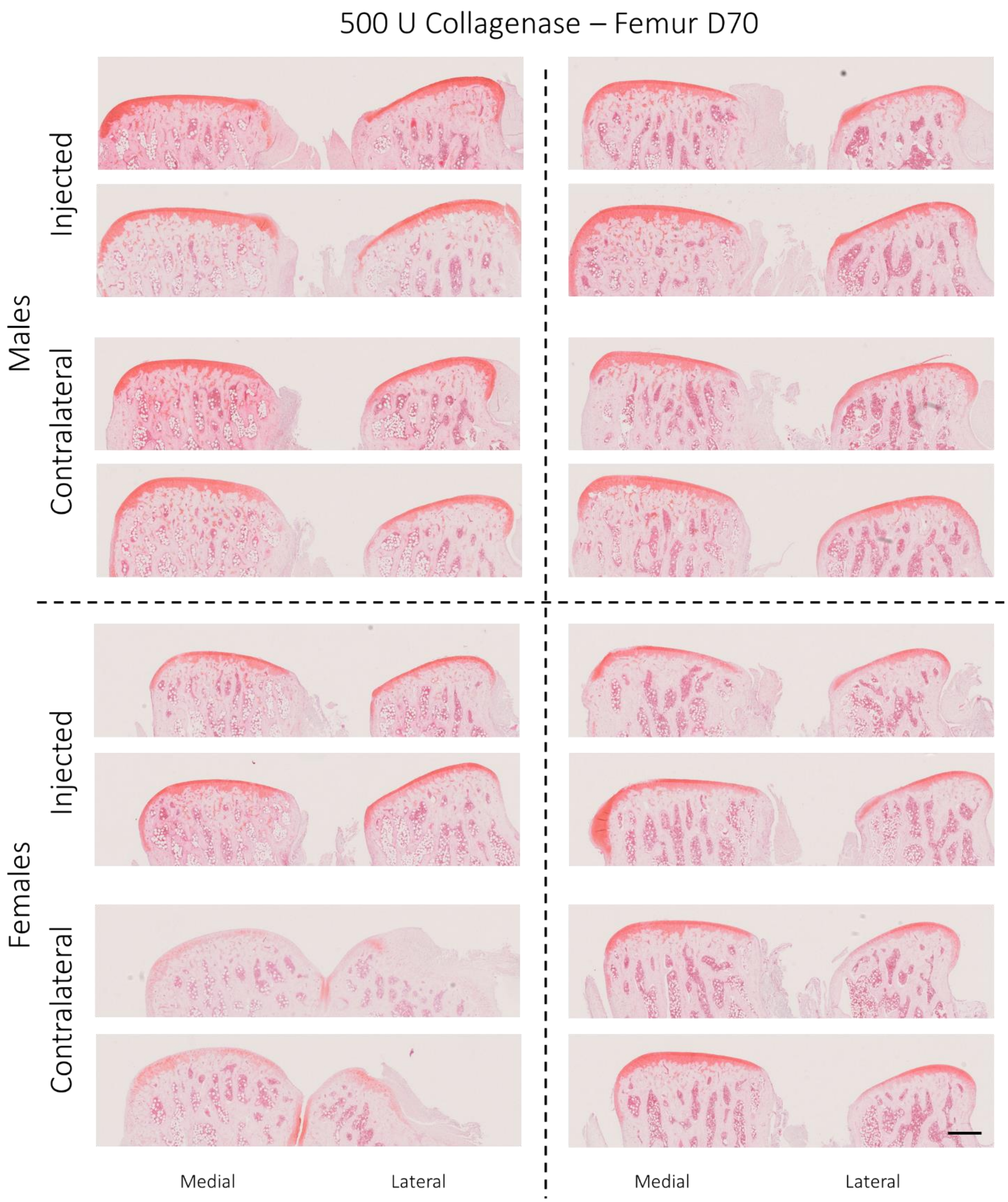

Figure S17: Complete safranin O histology of all the femurs in the 500 U collagenase group on day 70. There are two sections per joint. Scale bar: 500 µm

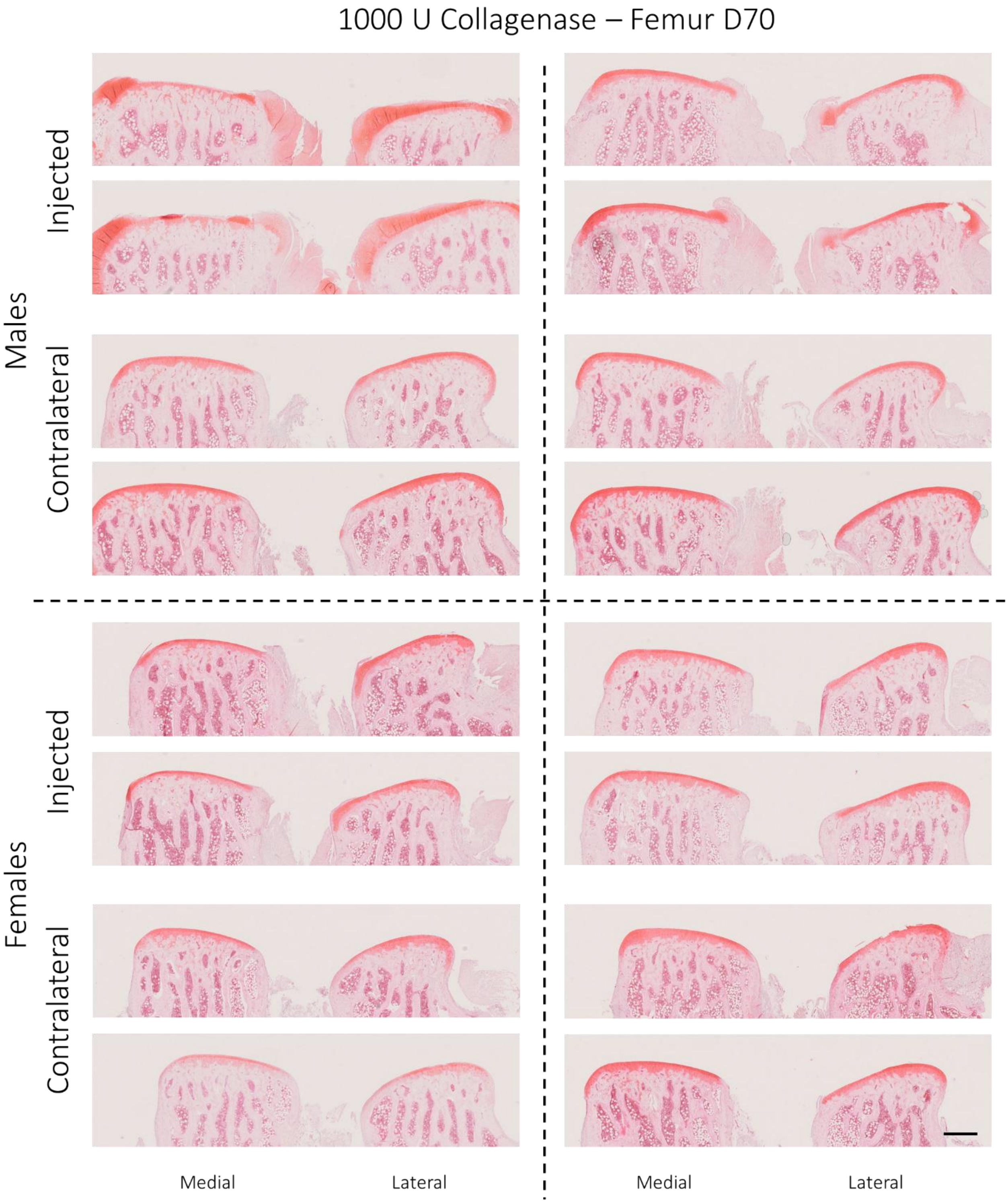

Figure S18: Complete safranin O histology of all the femurs in the 1000 U collagenase group on day 70. There are two sections per joint. Scale bar: 500 µm

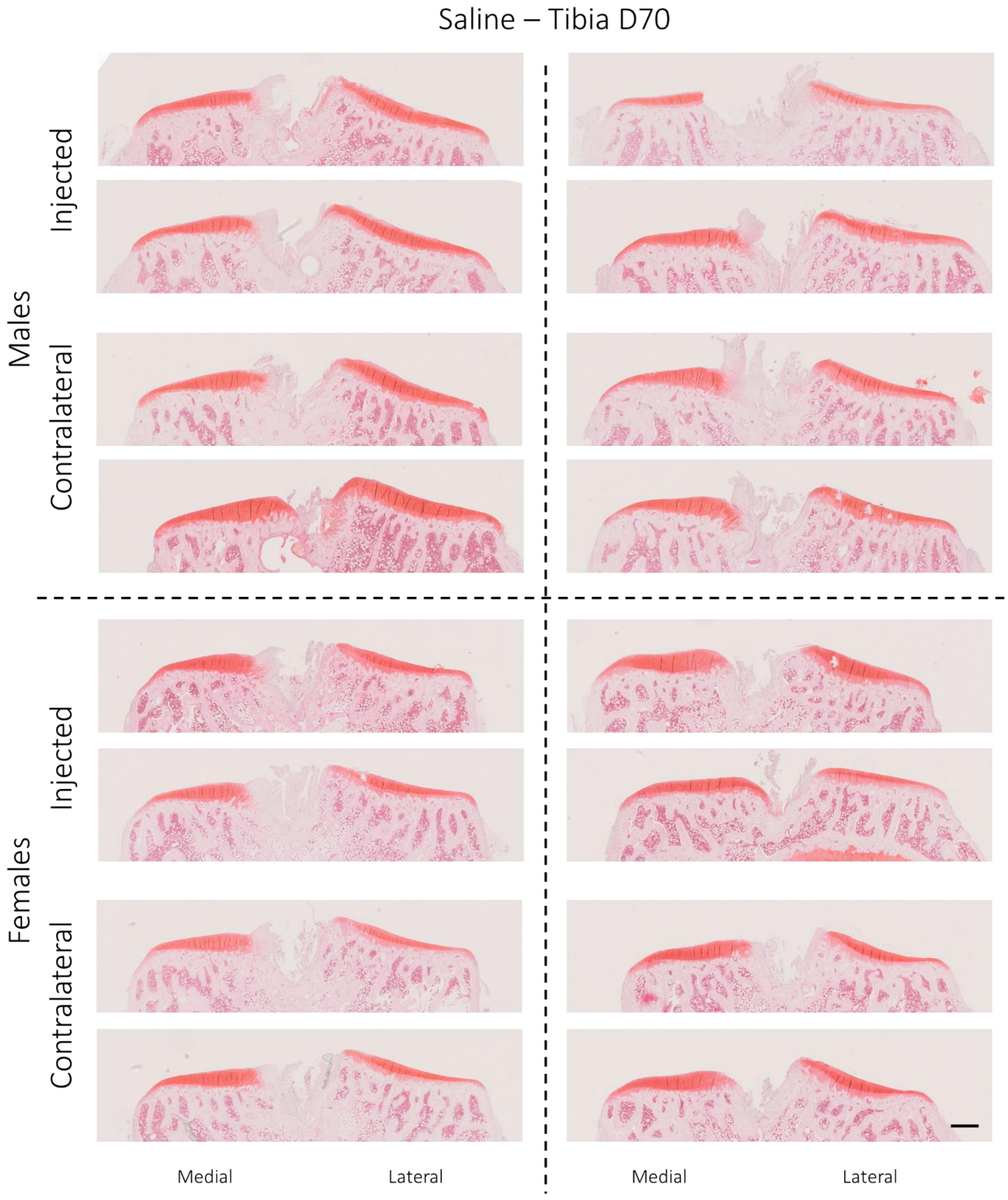

Figure S19: Complete safranin O histology of all the tibias in the saline group on day 70. There are two sections per joint. Scale bar: 500 µm

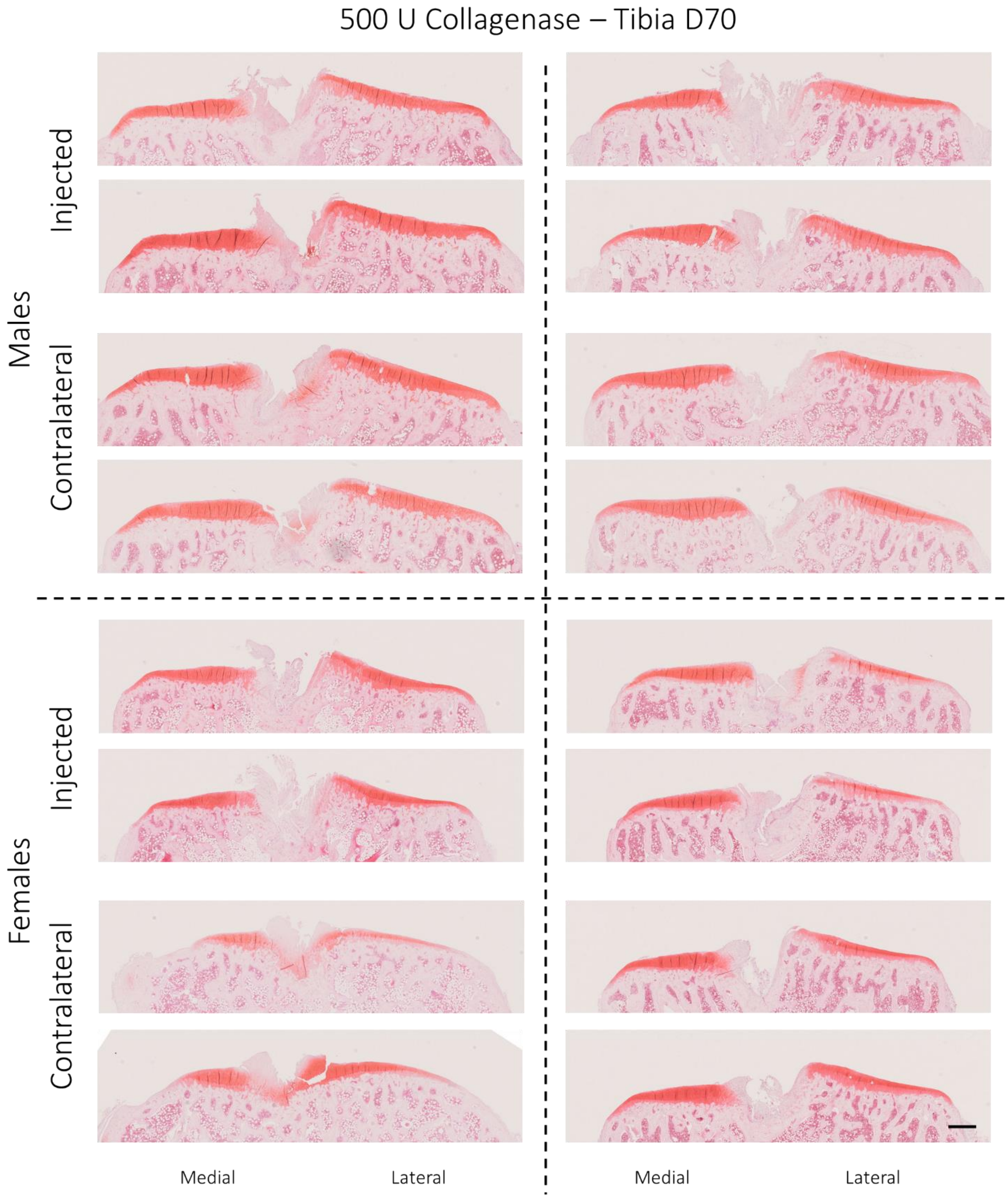

Figure S20: Complete safranin O histology of all the tibias in the 500 U collagenase group on day 70. There are two sections per joint. Scale bar: 500 µm

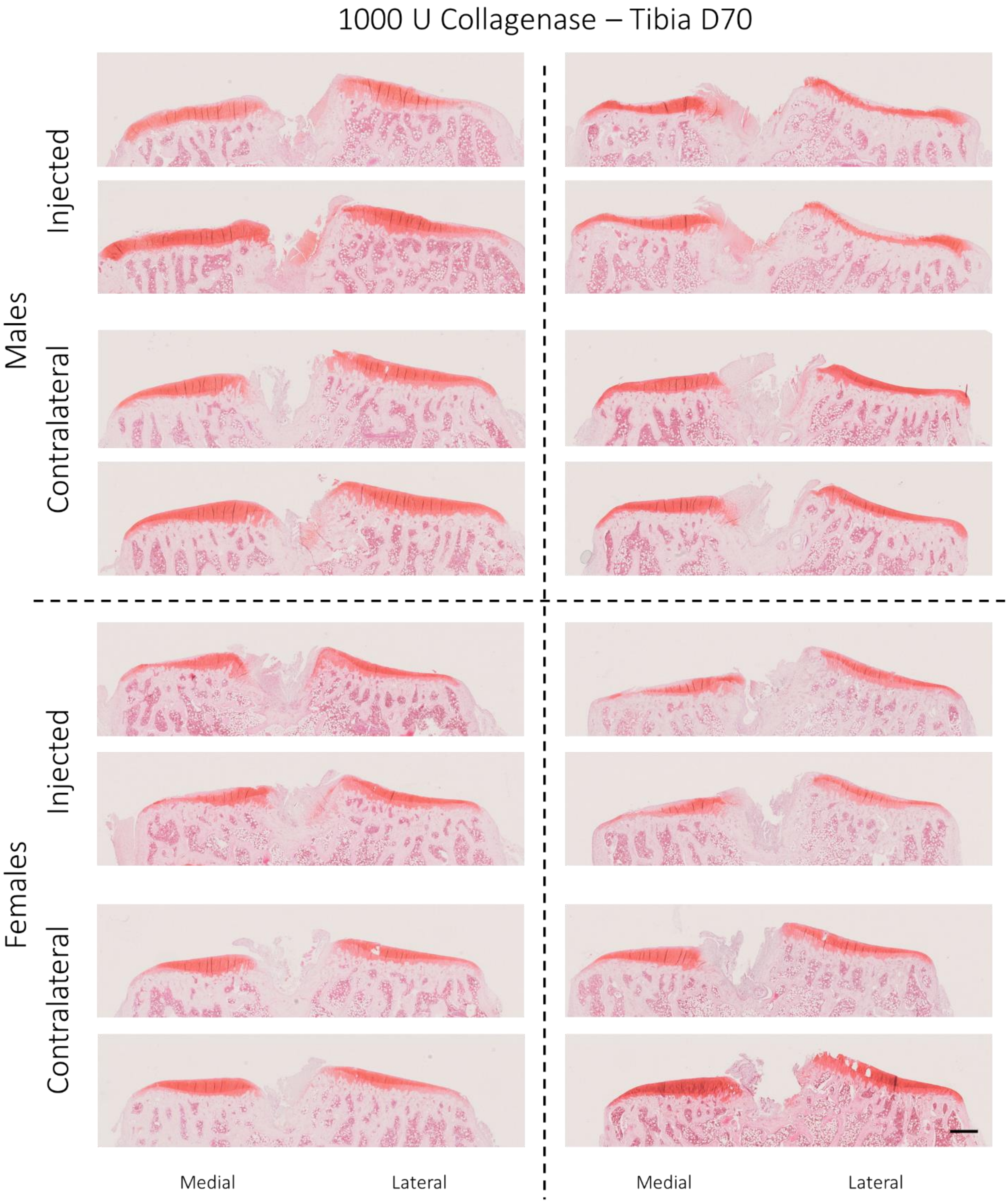

Figure S21: Complete safranin O histology of all the tibias in the 1000 U collagenase group on day 70. There are two sections per joint. Scale bar: 500 µm

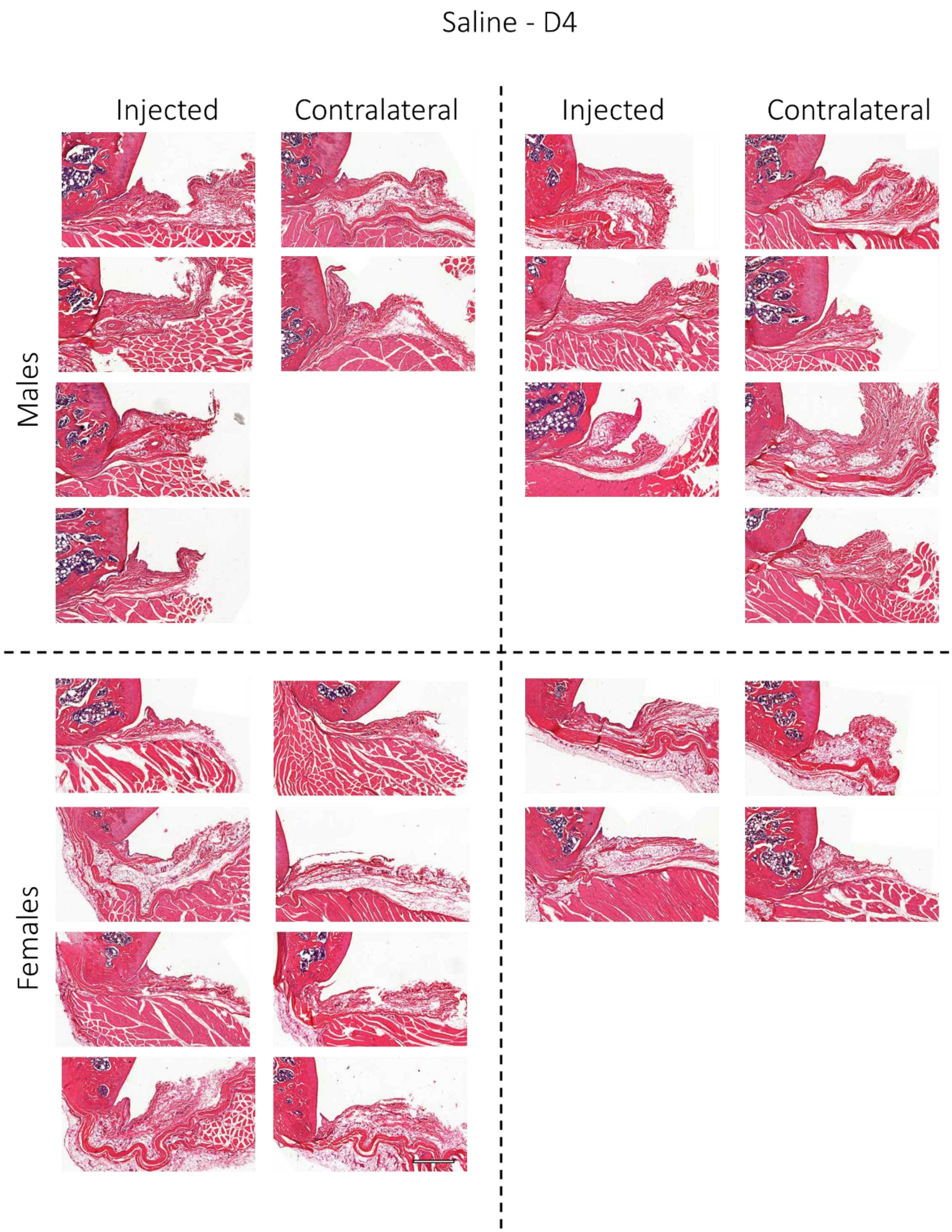

Figure S22: Complete H&E histology of all the synovia in the saline group on day 4. There are two sections per joint with two ROIs per section medially and laterally of the patella. Scale bar: 500 µm

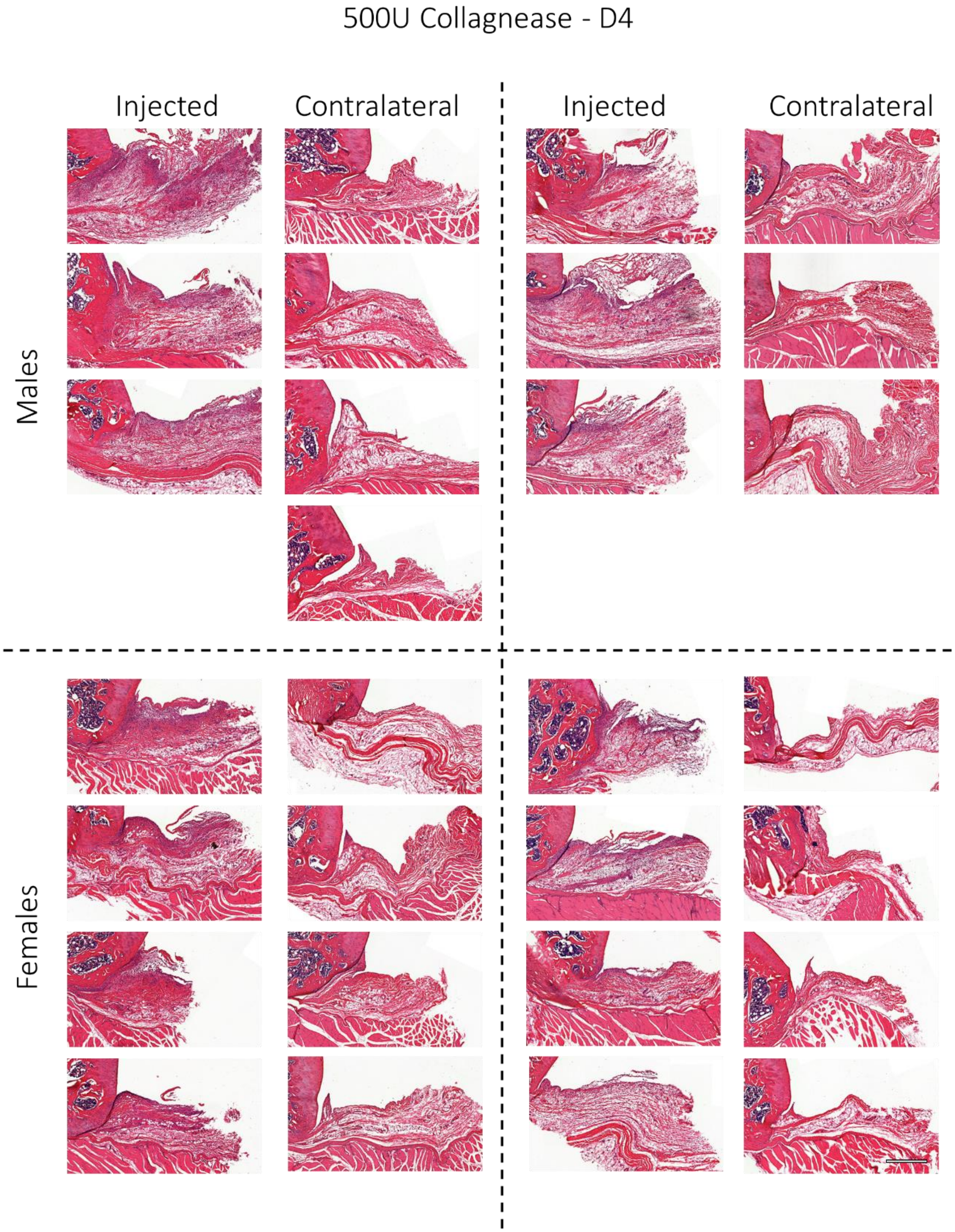

Figure S23: Complete H&E histology of all the synovia in the 500 U collagenase group on day 4. There are two sections per joint with two ROIs per section medially and laterally of the patella. Scale bar: 500 µm

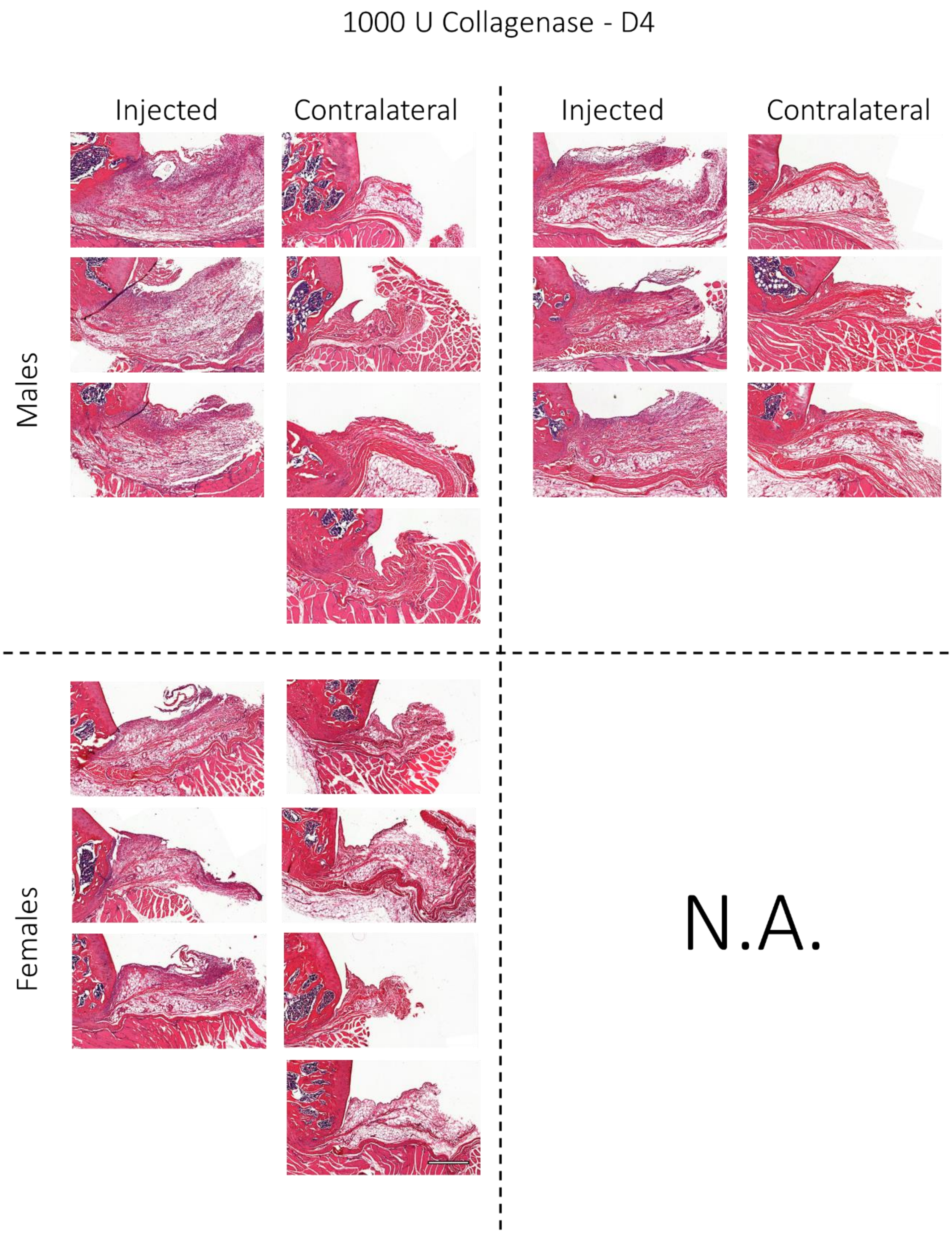

Figure S24: Complete H&E histology of all the synovia in the 1000 U collagenase group on day 4. There are two sections per joint with two ROIs per section medially and laterally of the patella. Note that the second female needed to be removed from the study due to a anterolateral tibial dislocation of the injected knee joint. Scale bar: 500 µm

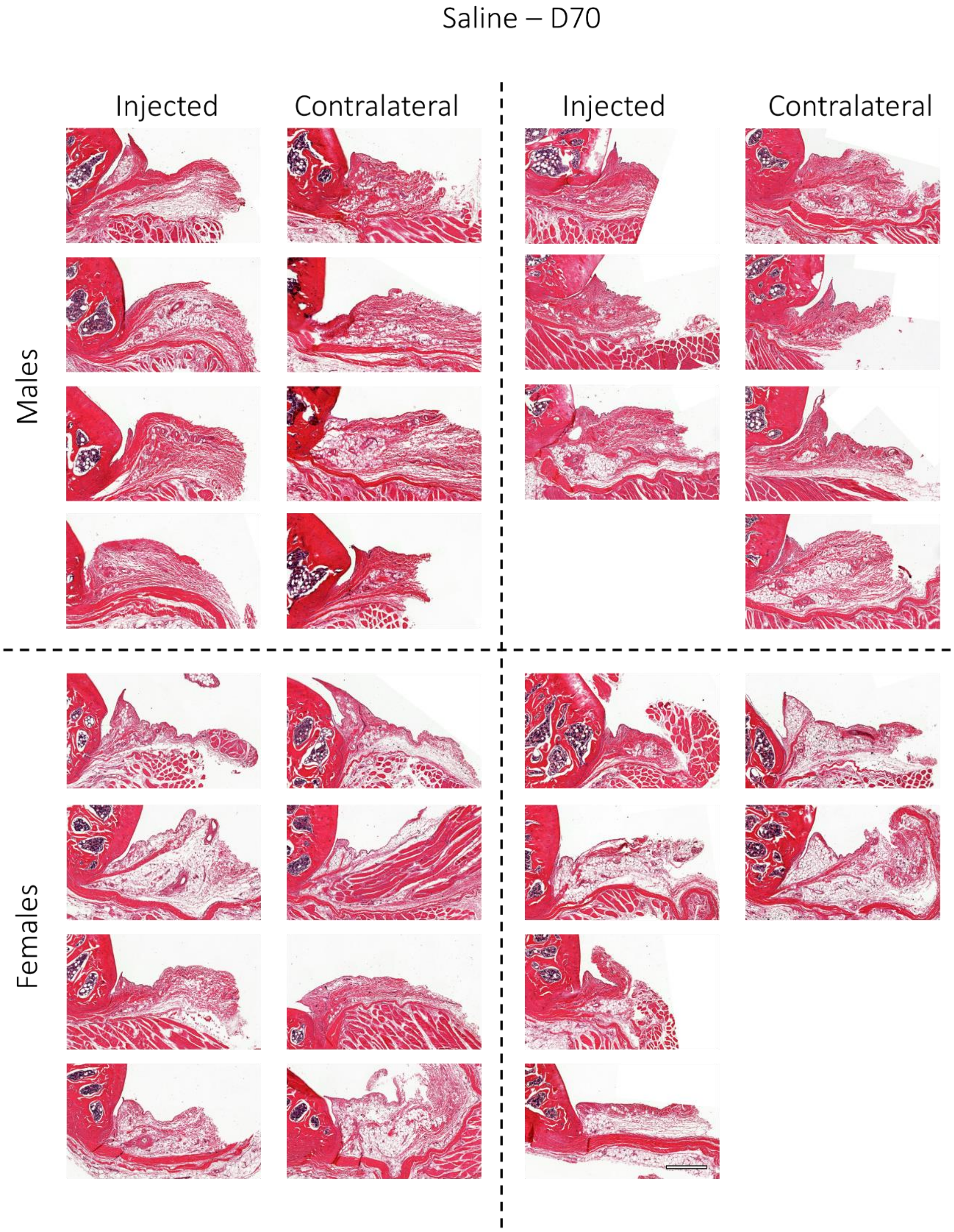

Figure S25: Complete H&E histology of all the synovia in the saline group on day 70. There are two sections per joint with two ROIs per section medially and laterally of the patella. Scale bar: 500 μm

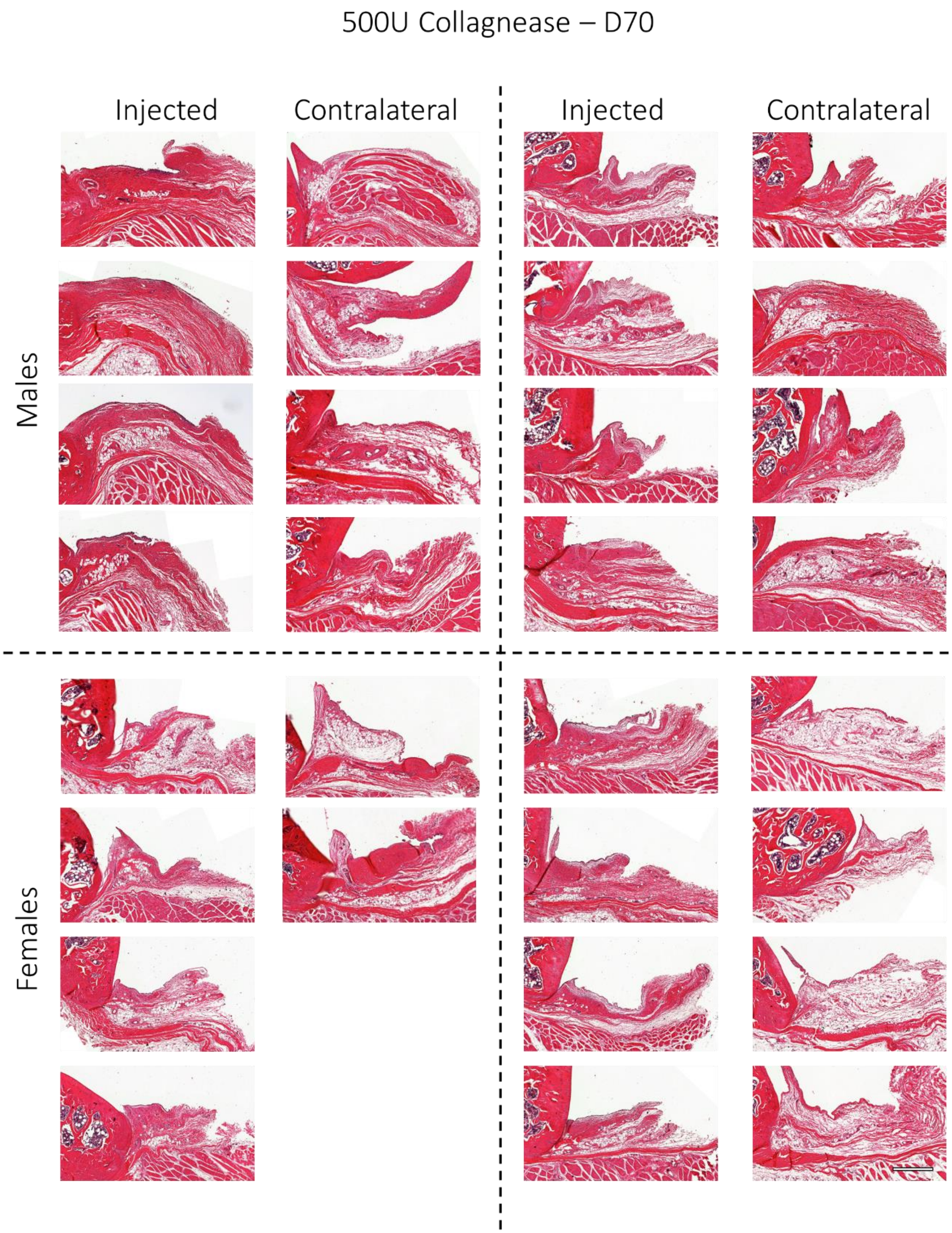

Figure S26: Complete H&E histology of all the synovia in the 500 U collagenase group on day 70. There are two sections per joint with two ROIs per section medially and laterally of the patella. Scale bar: 500 μm

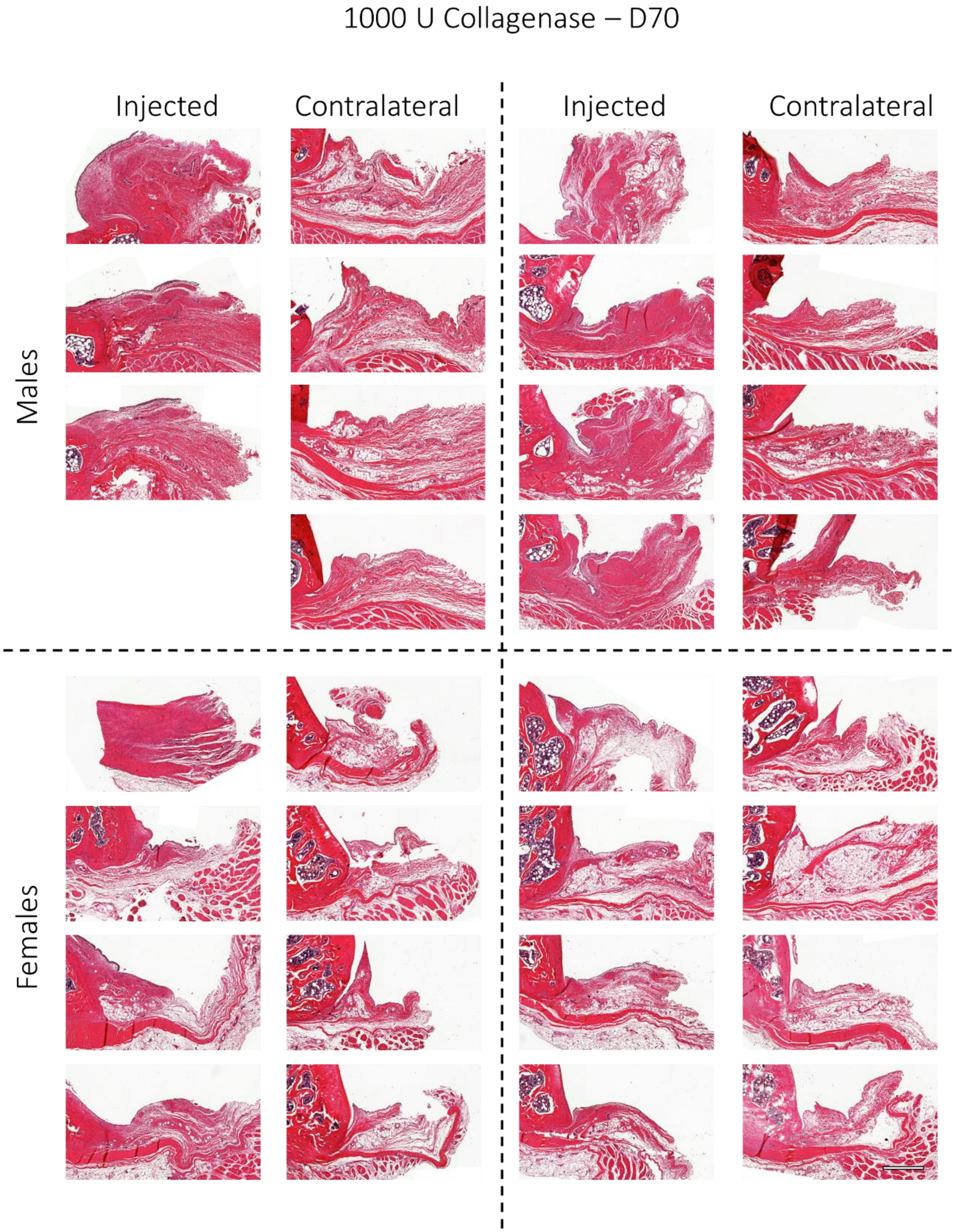

Figure S27: Complete H&E histology of all the synovia in the 1000 U collagenase group on day 70. There are two sections per joint with two ROIs per section medially and laterally of the patella. Scale bar: 500 μm

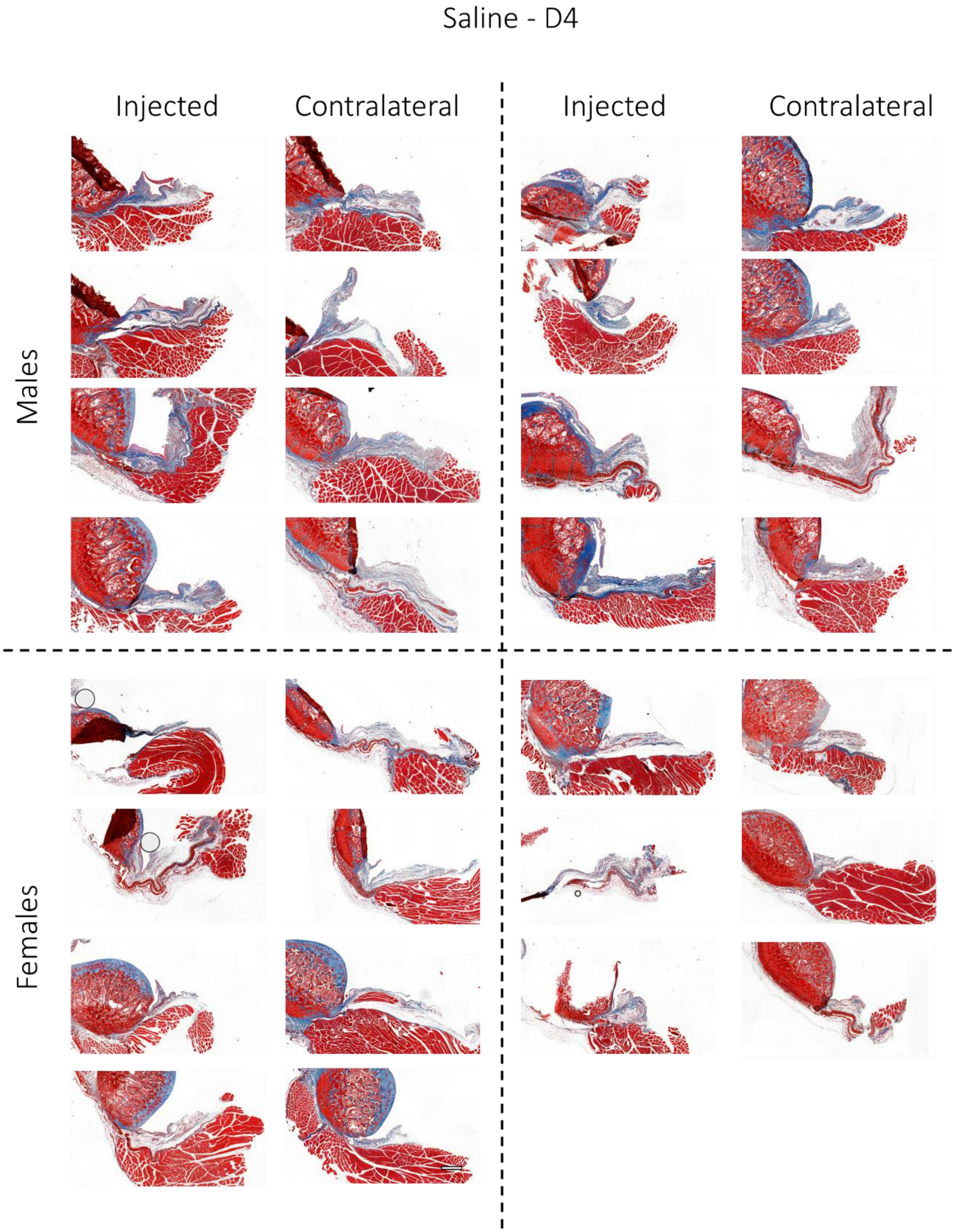

Figure S28: Complete Masson’s trichrome histology of all the synovia in the saline group on day 4. There are two sections per joint with two ROIs per section medially and laterally of the patella. Scale bar: 500 µm

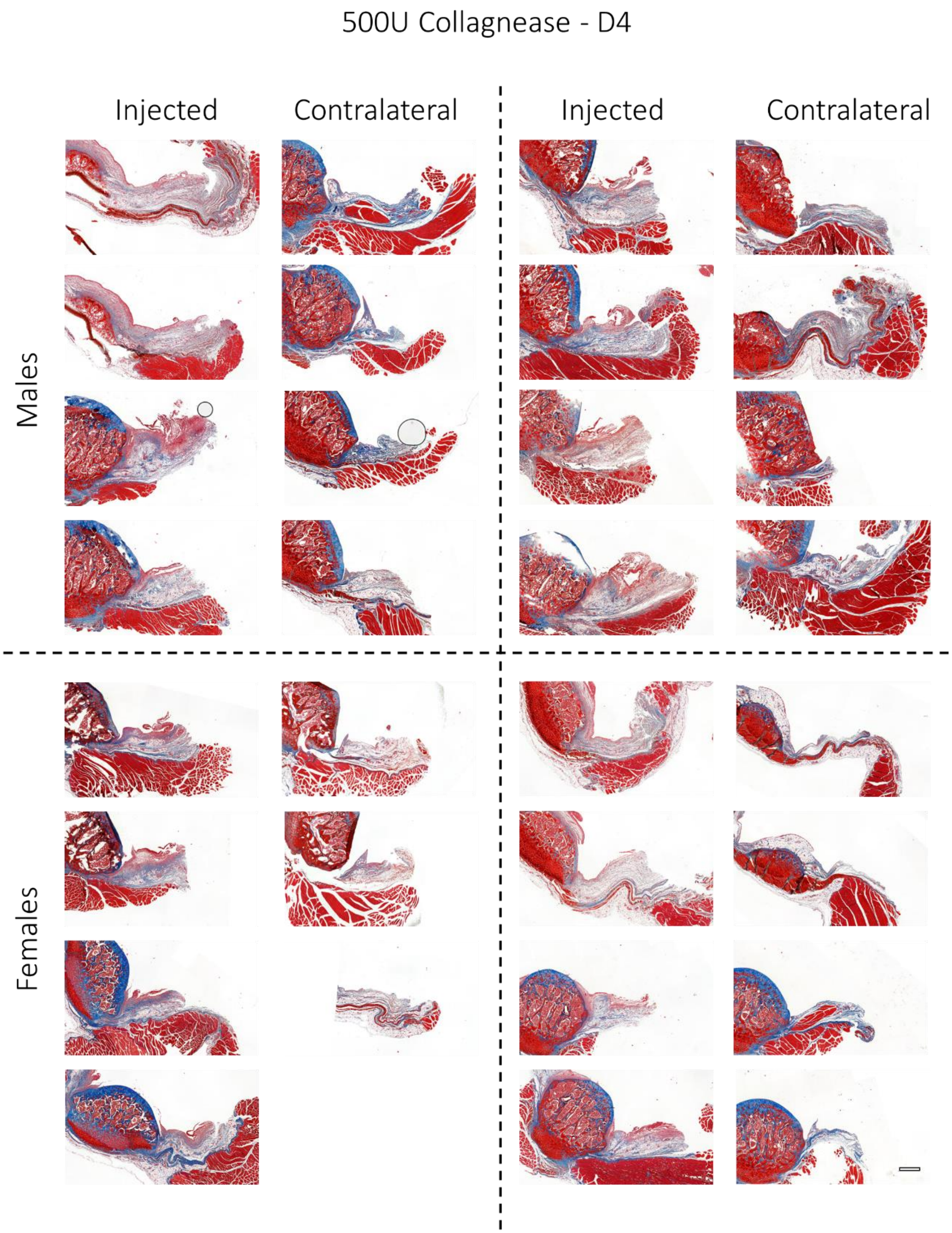

Figure S29: Complete Masson's trichrome histology of all the synovia in the 500 U collagenase group on day 4. There are two sections per joint with two ROIs per section medially and laterally of the patella. Scale bar: 500 µm

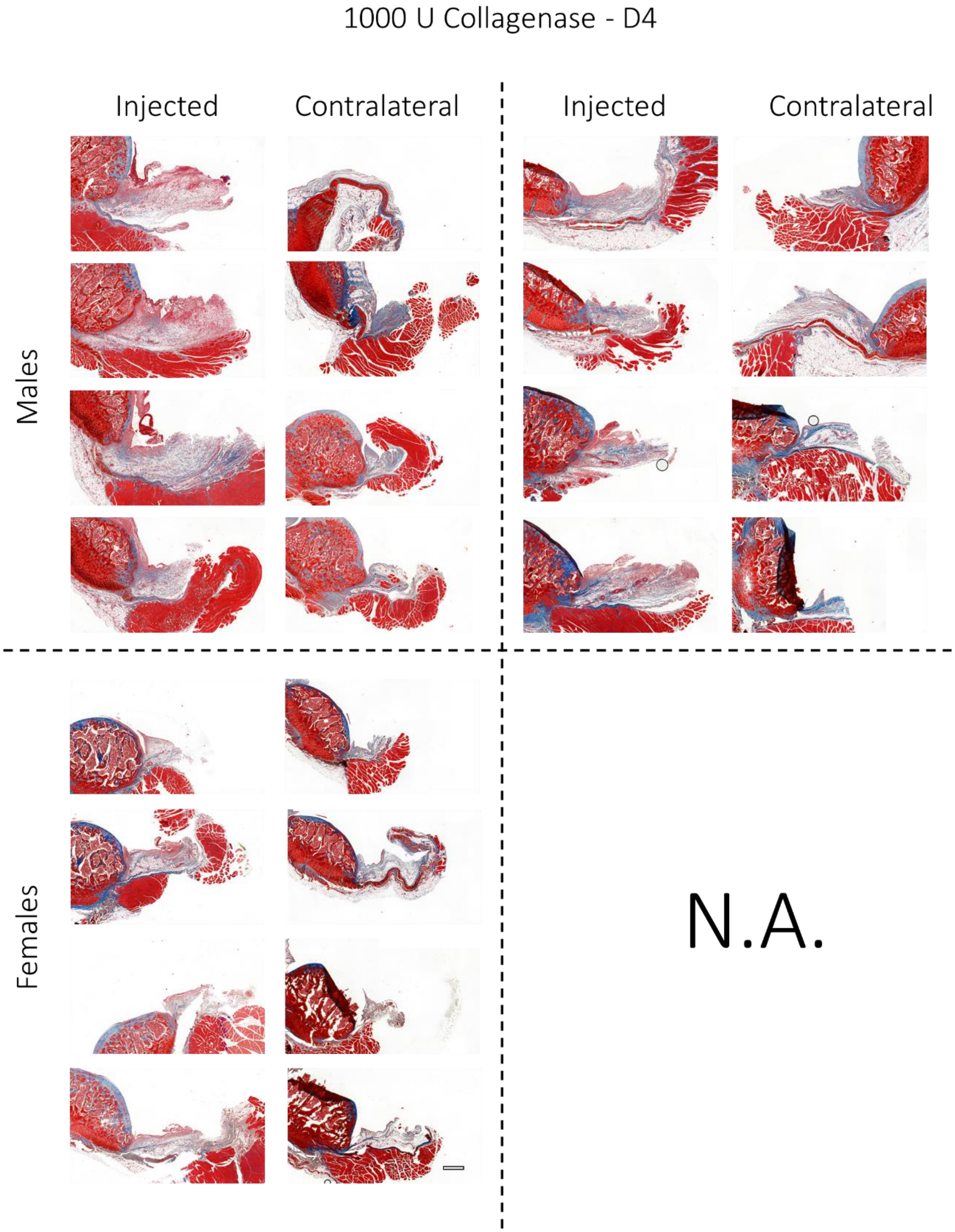

Figure S30: Complete Masson's trichrome histology of all the synovia in the 1000 U collagenase group on day 4. There are two sections per joint with two ROIs per section medially and laterally of the patella. Note that the second female needed to be removed from the study due to a anterolateral tibial dislocation of the injected knee joint. Scale bar: 500 µm

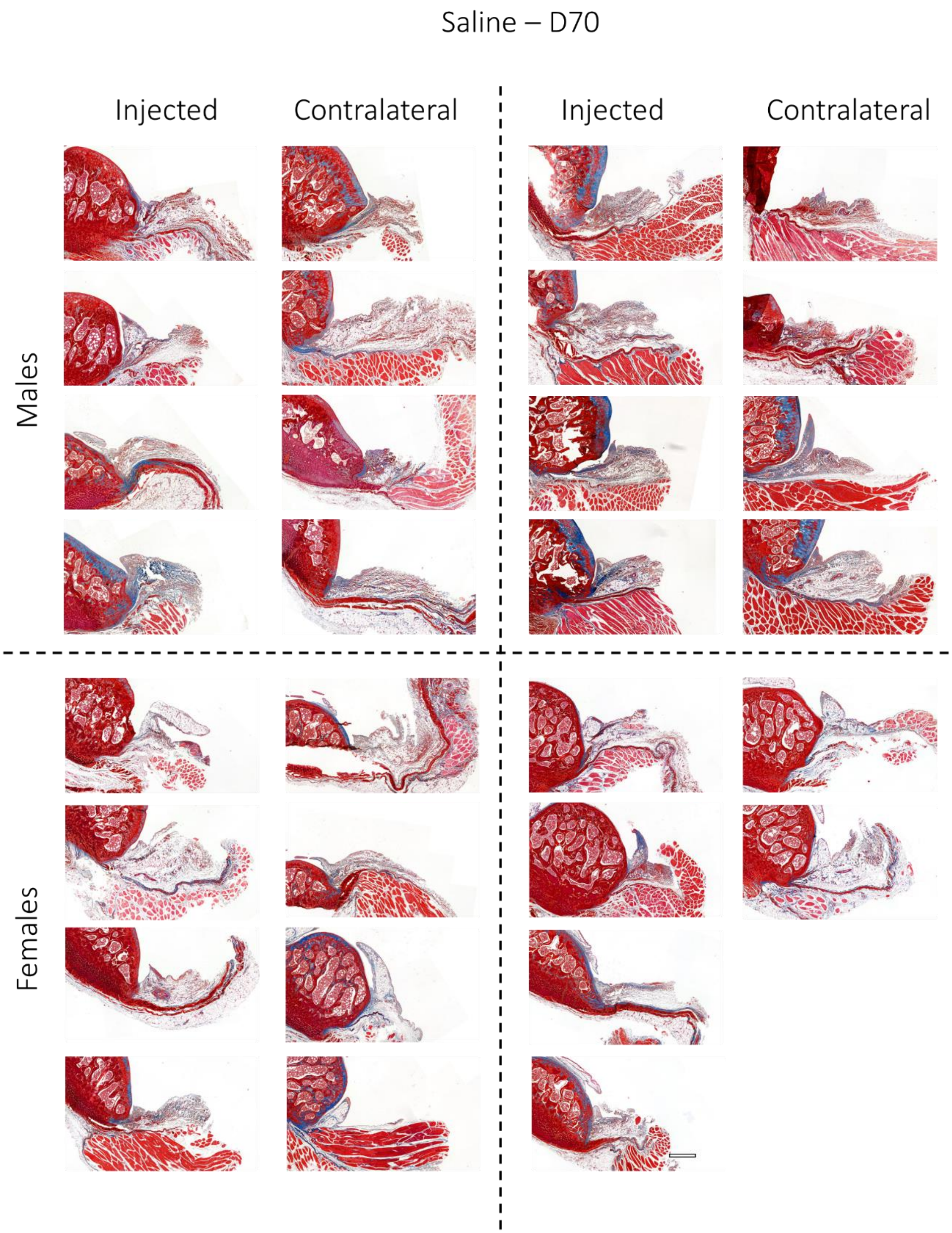

Figure S31: Complete Masson's trichrome histology of all the synovia in the saline group on day 70. There are two sections per joint with two ROIs per section medially and laterally of the patella. Scale bar: 500 µm

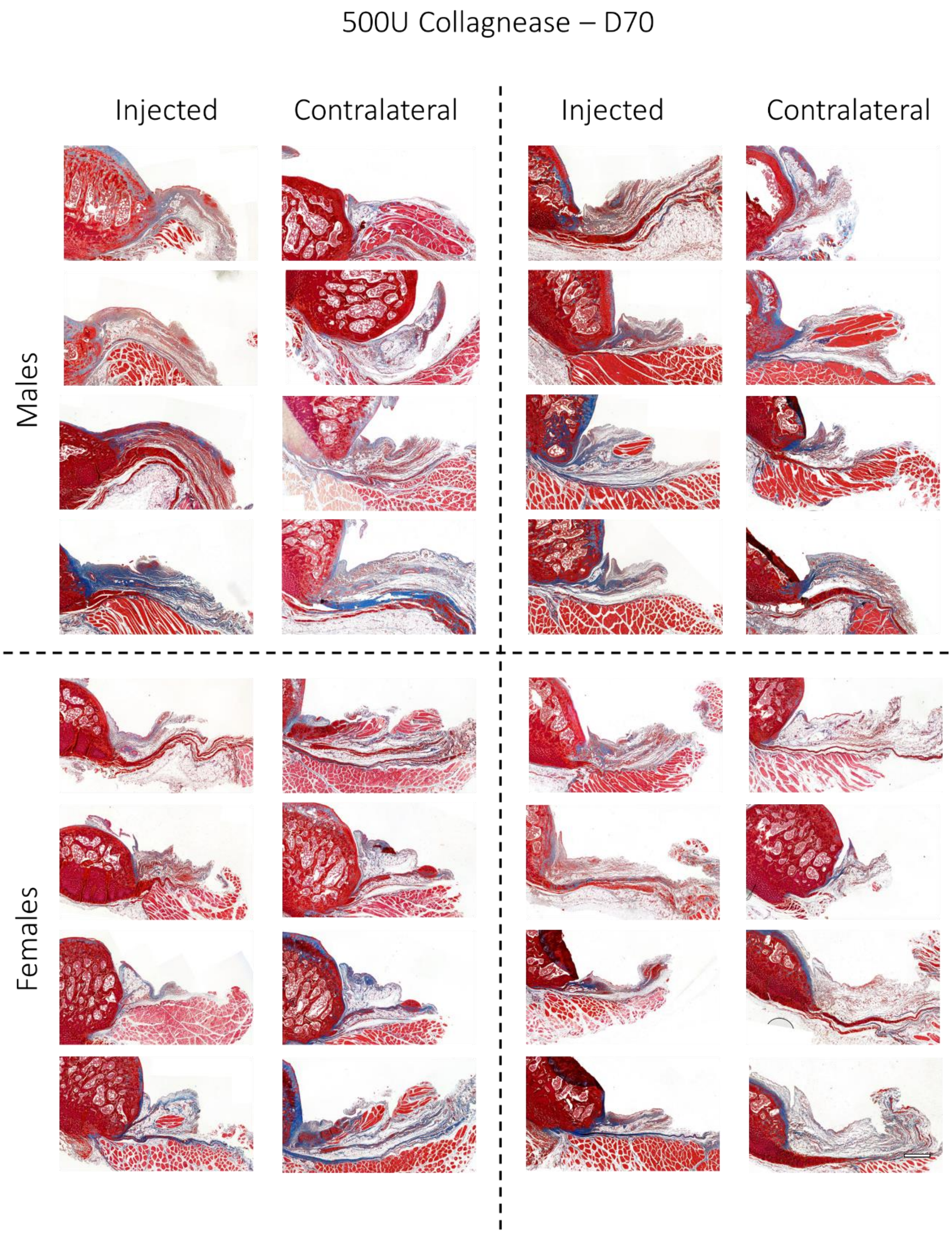

Figure S32: Complete Masson's trichrome histology of all the synovia in the 500 U collagenase group on day 70. There are two sections per joint with two ROIs per section medially and laterally of the patella. Scale bar: 500 µm

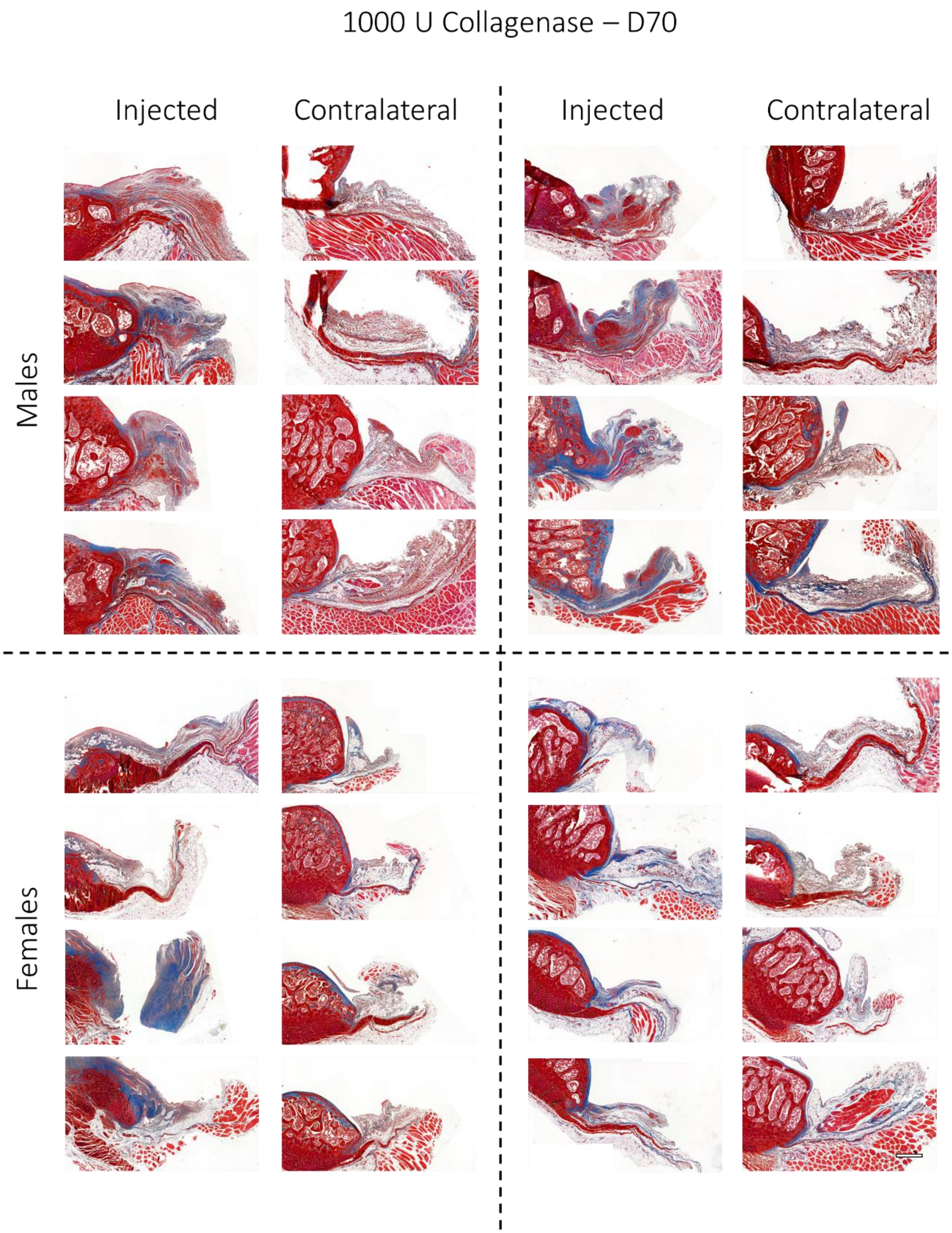

Figure S33: Complete Masson's trichrome histology of all the synovia in the 1000 U collagenase group on day 70. There are two sections per joint with two ROIs per section medially and laterally of the patella. Scale bar: 500 µm

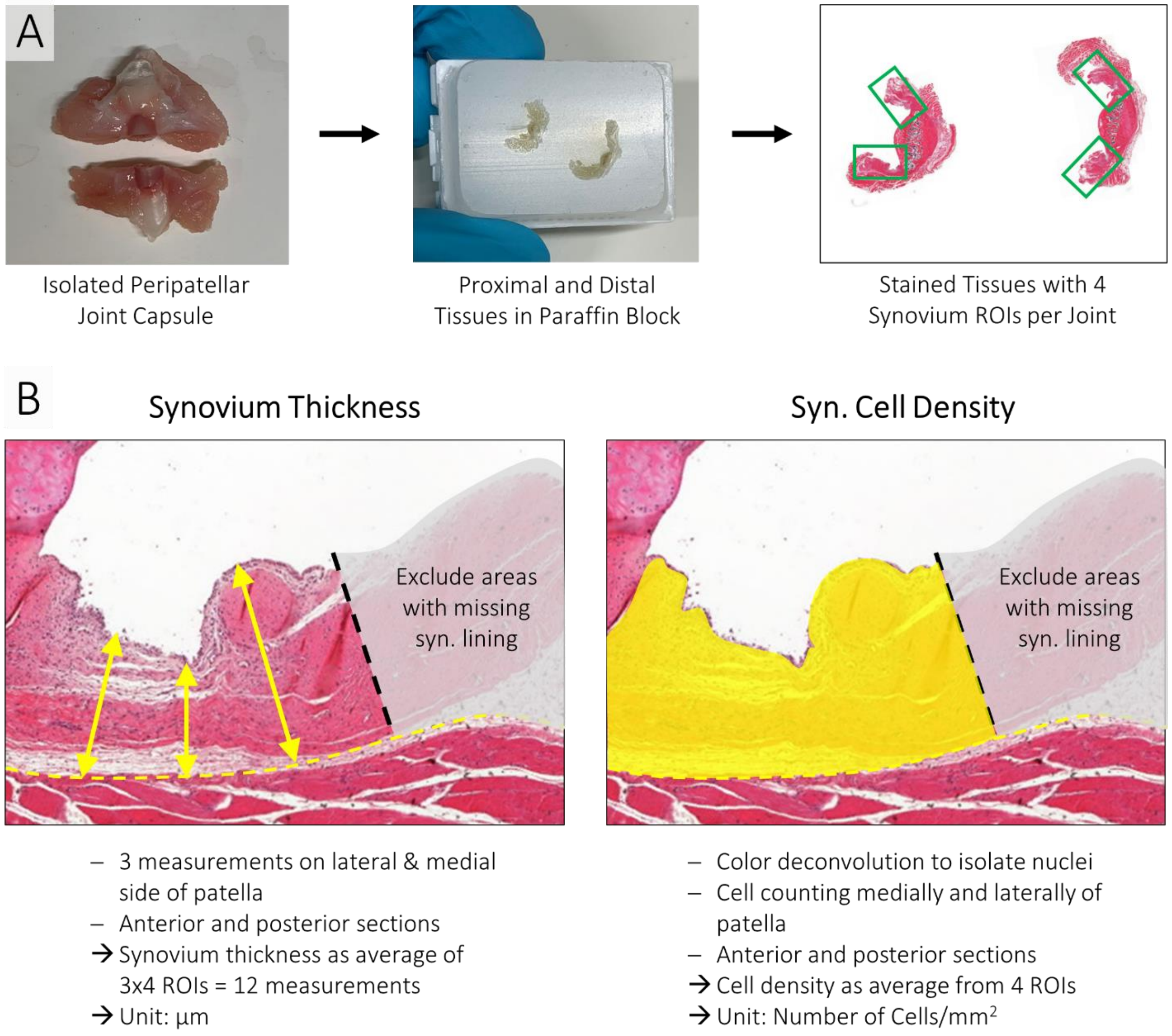

Figure S34 – Synovium Thickness and Cell Density Quantification: Schematic illustration of the methodology to process (A) and analyze (B) the peripatellar synovia with regard to the synovium thickness and cell density.

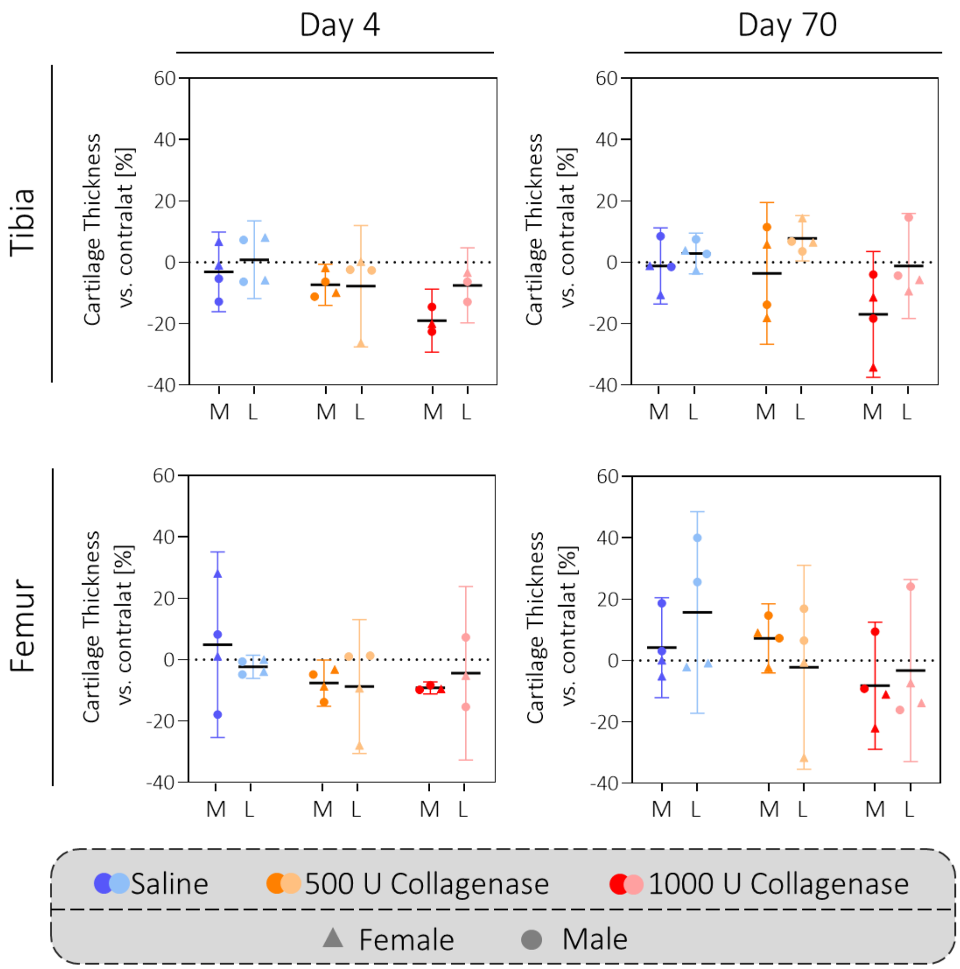

Figure S35: Collagenase injection leads to a decrease in cartilage thickness on both day 4 and day 70: Assessment of global cartilage thickness in computed tomography scans indicates a decreased cartilage thickness for the 1000 U collagenase group at both timepoints and all locations. The trend is particularly strong in the medial tibia. M: medial, L: lateral. N=4 (N=3 for day 4/1000 U collagenase).

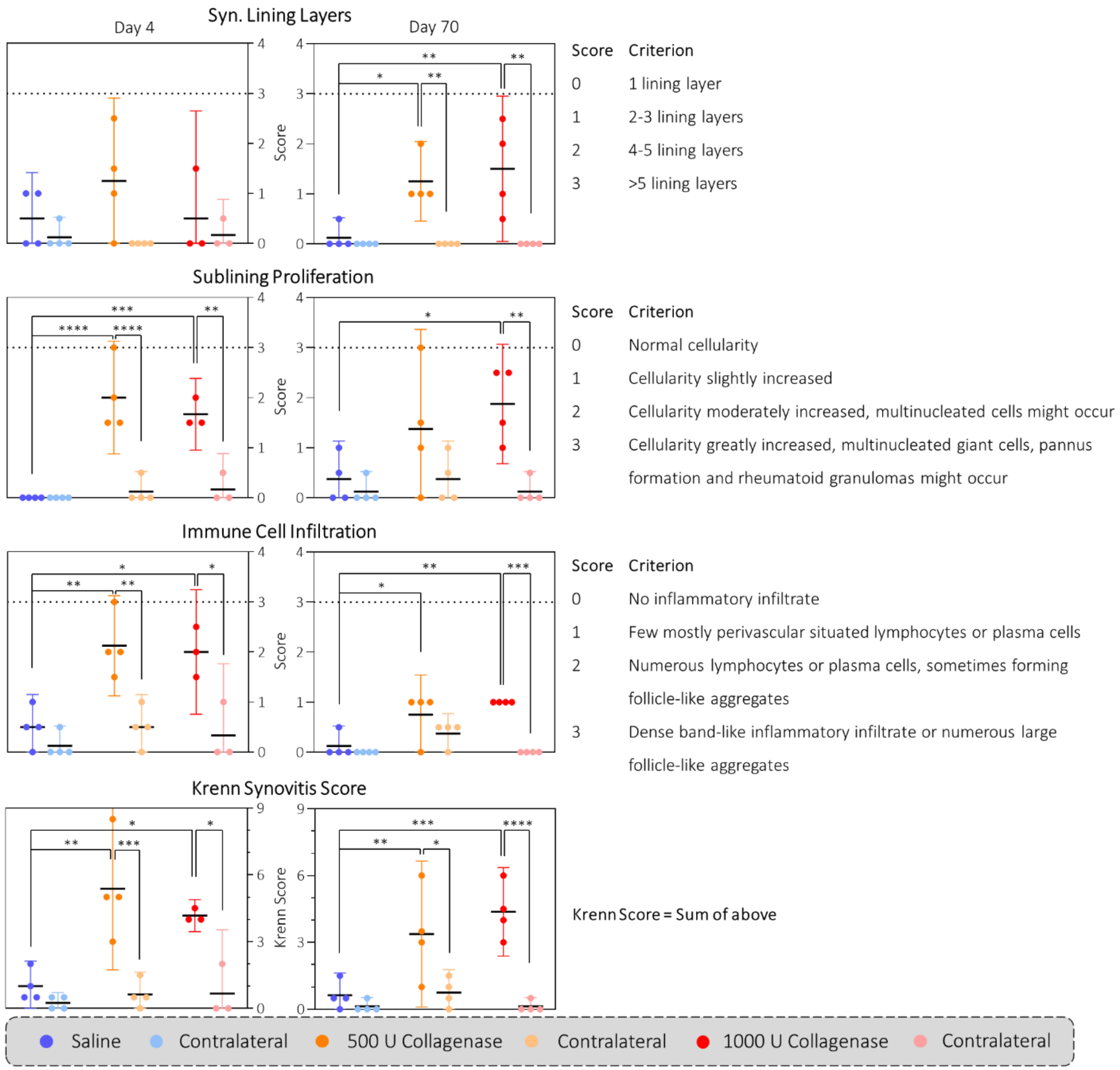

Figure S36 – Krenn Synovitis Score Subcategories: Splitting the Krenn synovitis score into its three respective subcategories shows that the collagenase-injected samples were generally scored very similarly regardless of the subcategory and the timepoint. The only exception is the immune cell category where there was a higher degree of infiltration on day 4 than on day 70. N=4

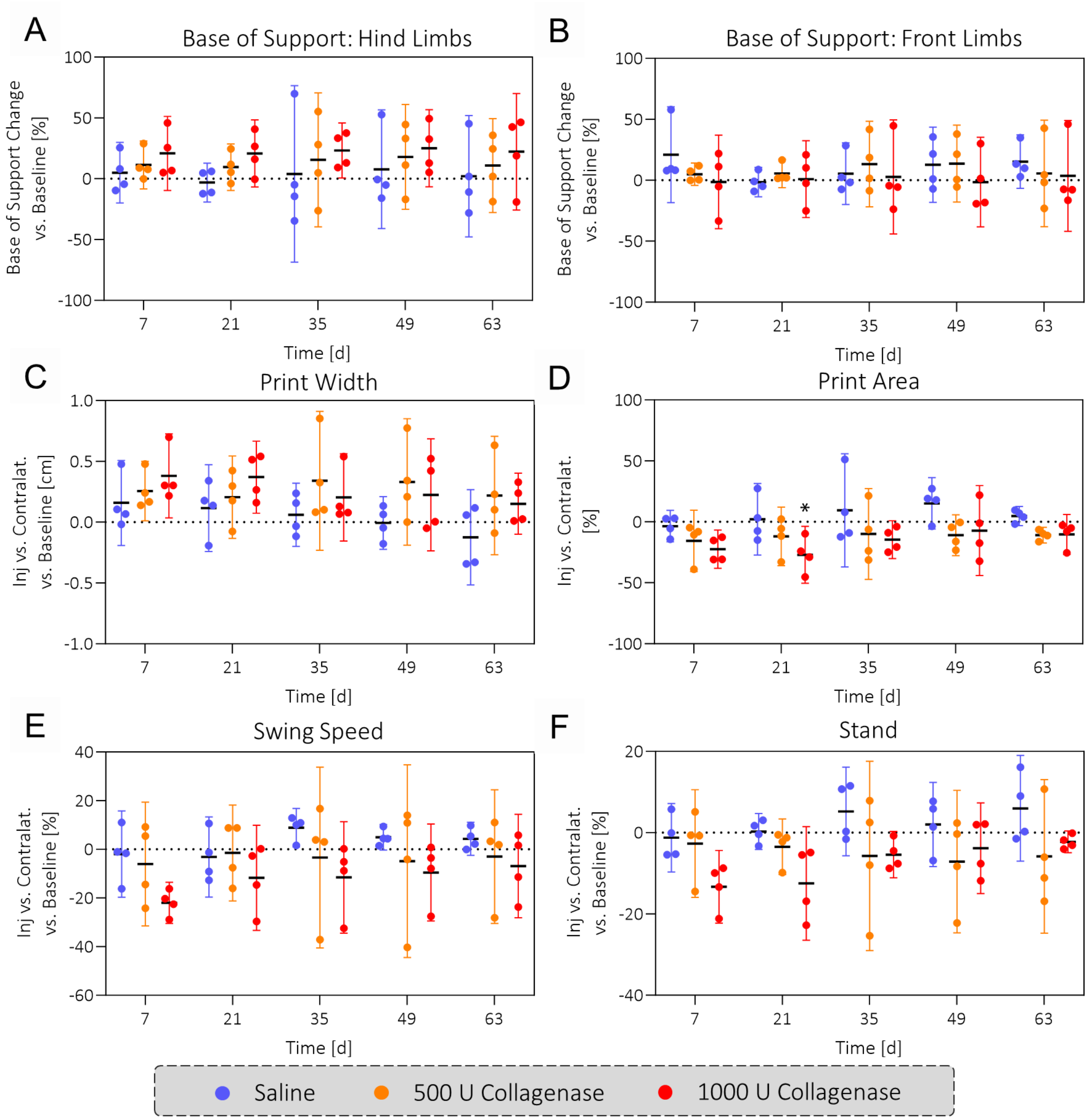

Figure S37 – Additional CatWalk Data: See Figure S4 for explanations of the different metrics. N=4

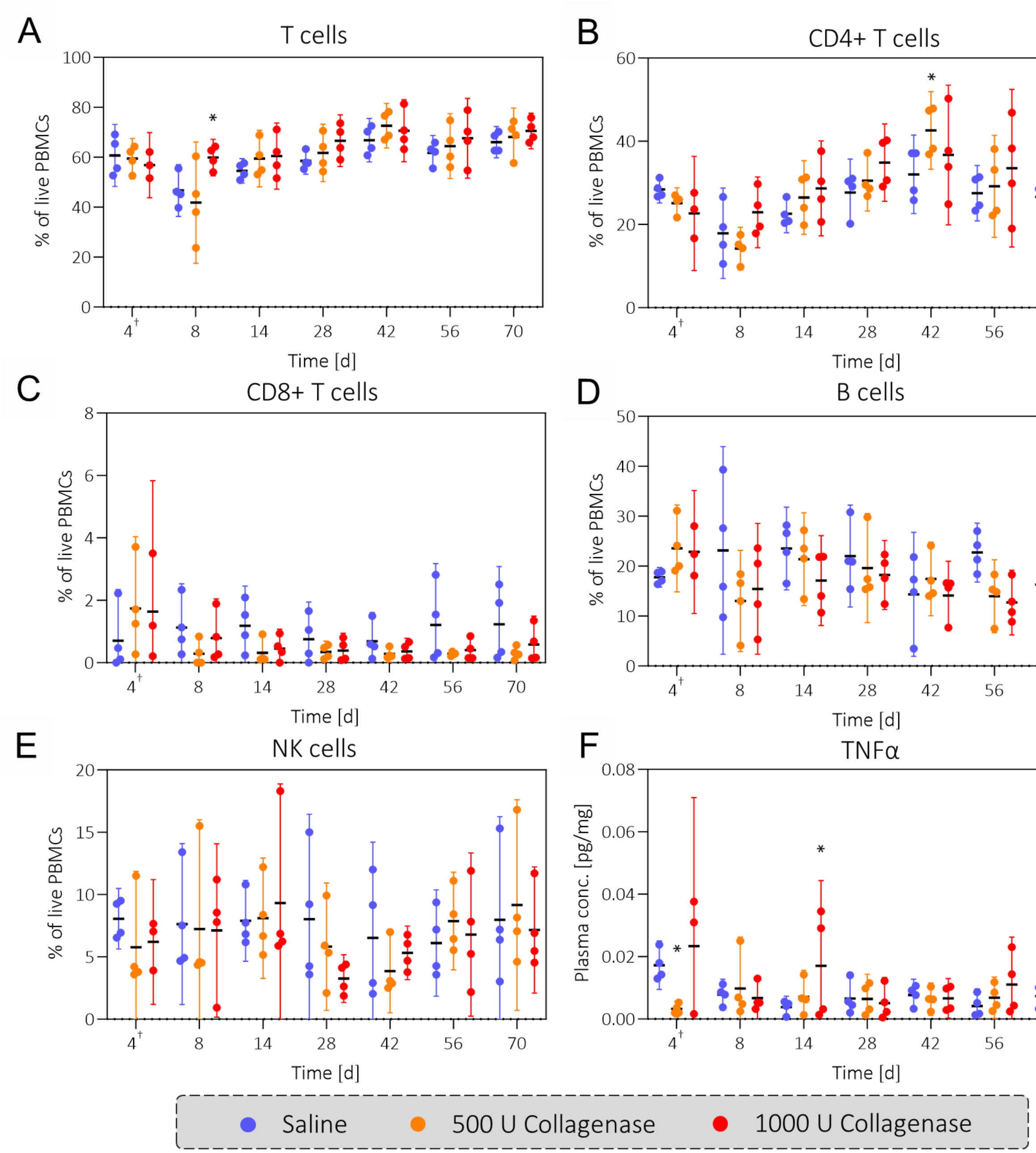

Figure S38 – Additional plasma cytokines and PBMC cell types: Note that the blood from day 4 was collected from the animals being euthanized that day (†). N=4

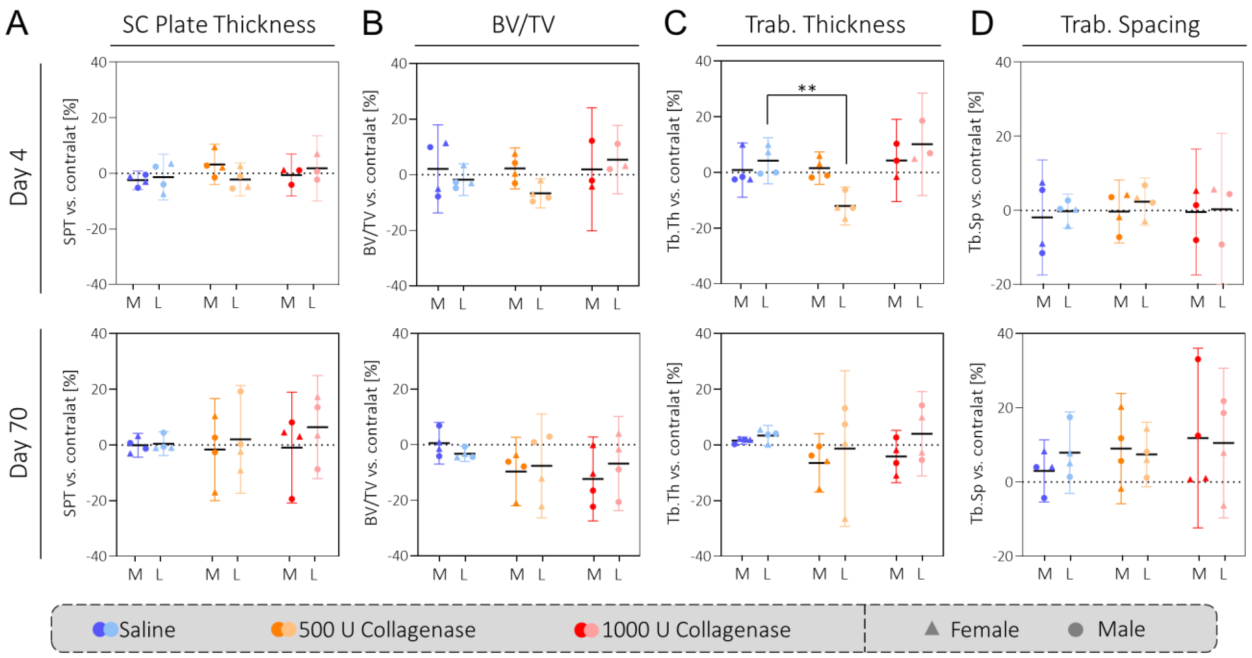

Figure S39 – Bone analysis results for femur: The bone results for the femurs indicate faint trends towards a decrease in BV/TV (B), trabecular thickness (C) and an increase in trabecular spacing (D) on the medial side on day 70. Regarding subchondral bone plate thickness (A), no trends were observed. M: medial, L: lateral. N=4 (N=3 for day 4/1000 U collagenase).

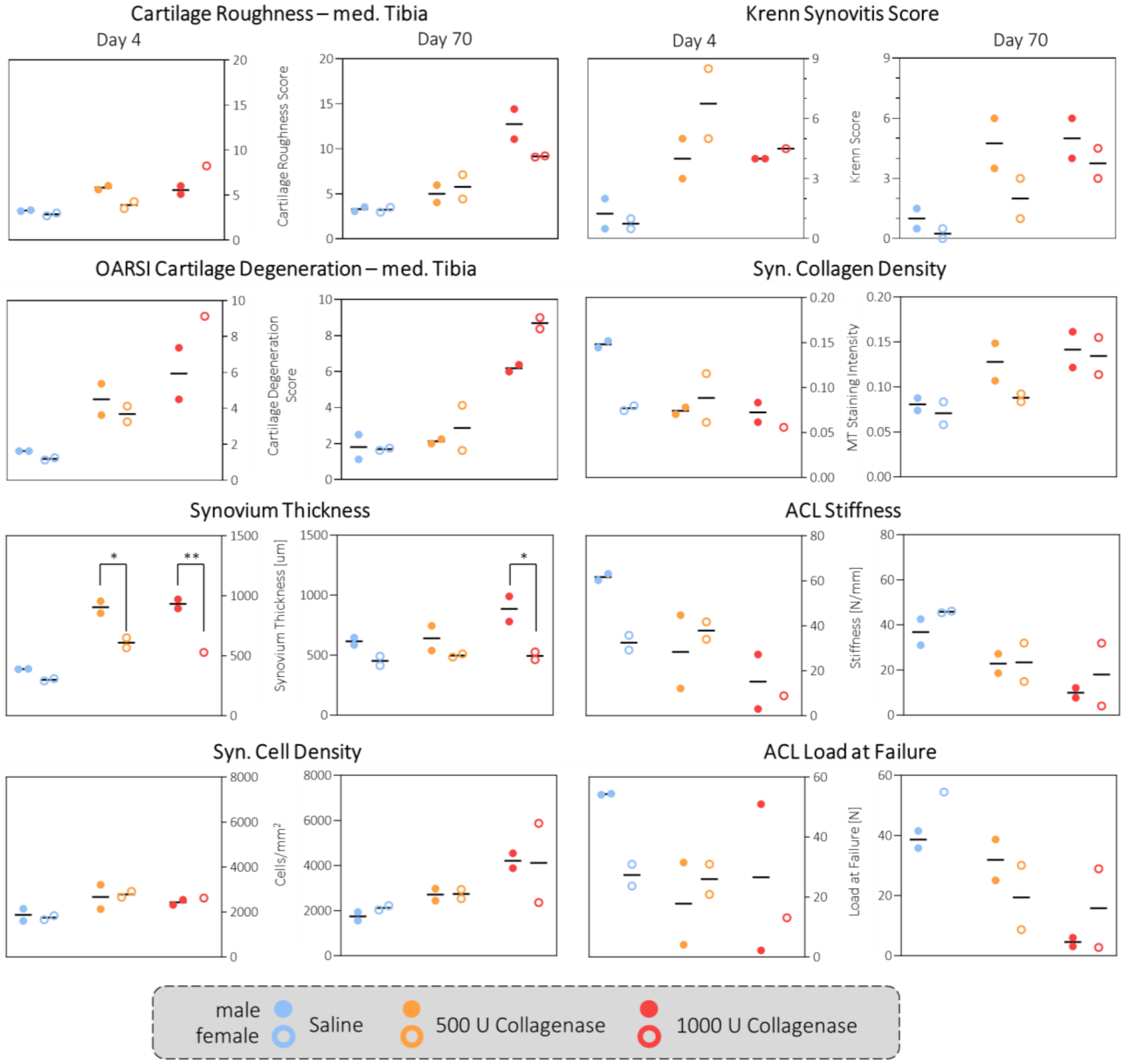

Figure S40 – Comparison of Males & Females pt.1: The data corresponds to the plots from figures 2-4 in the main text.

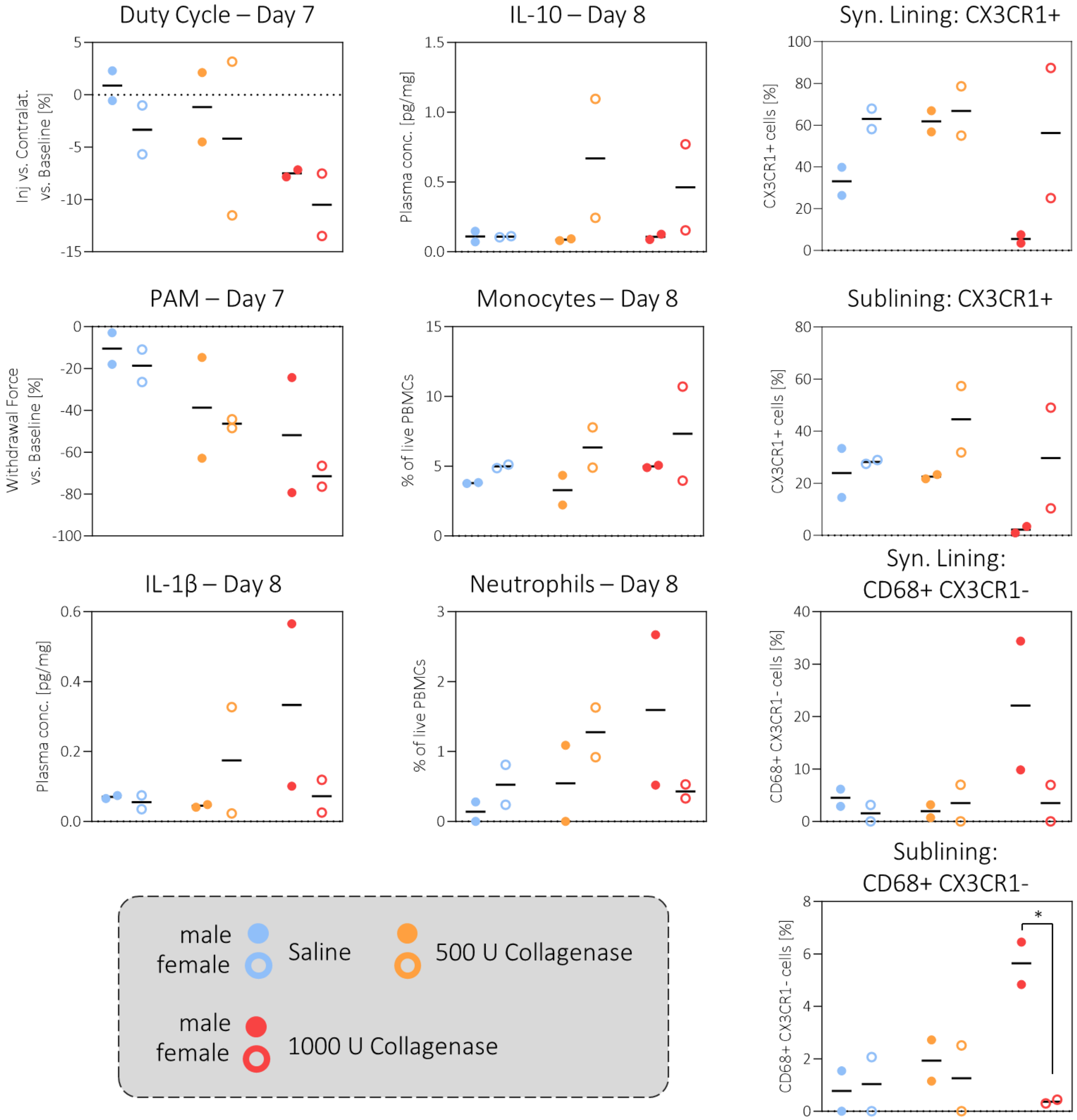

Figure S41 – Comparison of Males & Females pt.2: The data corresponds to the plots from figures 5 & 6 in the main text.

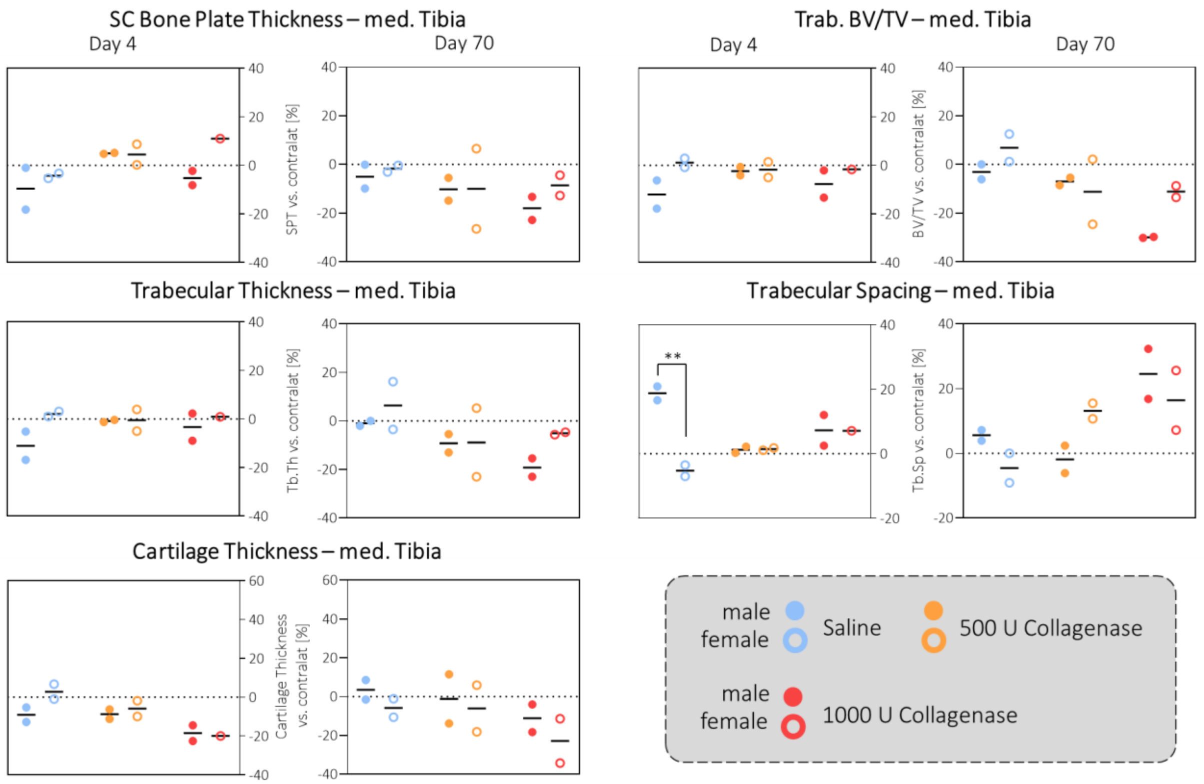

Figure S42 – Comparison of Males & Females pt.3: The data corresponds to the plots from figures 7 and S35.

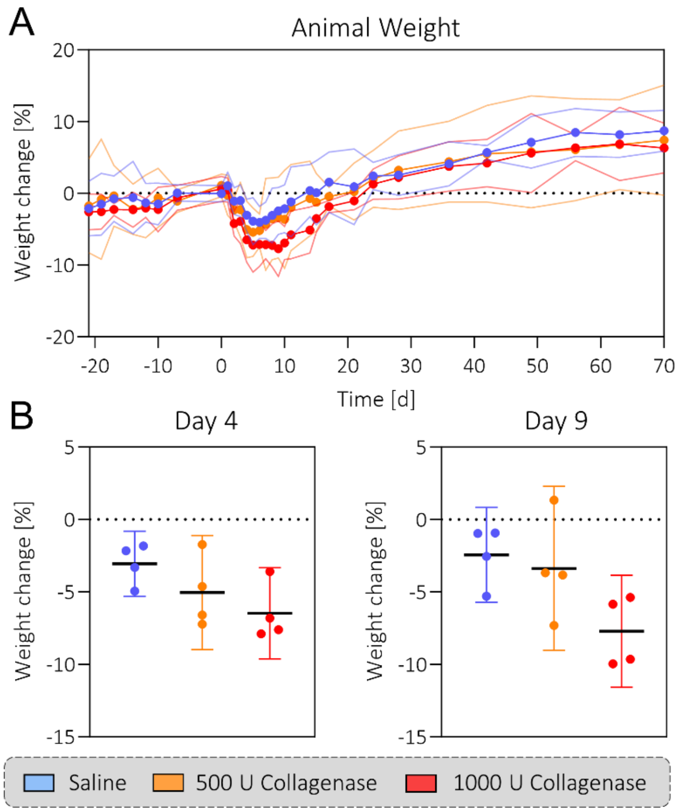

Figure S43 – 1000 U collagenase injection causes increased weight loss: A) Weight change normalized to the average weight of days -7 and 0. Lines indicate 95% CI. B) Compared to day 4, the 1000 U collagenase group further loses weight until day 9, whereas saline and 500 U collagenase groups gain weight. N=4

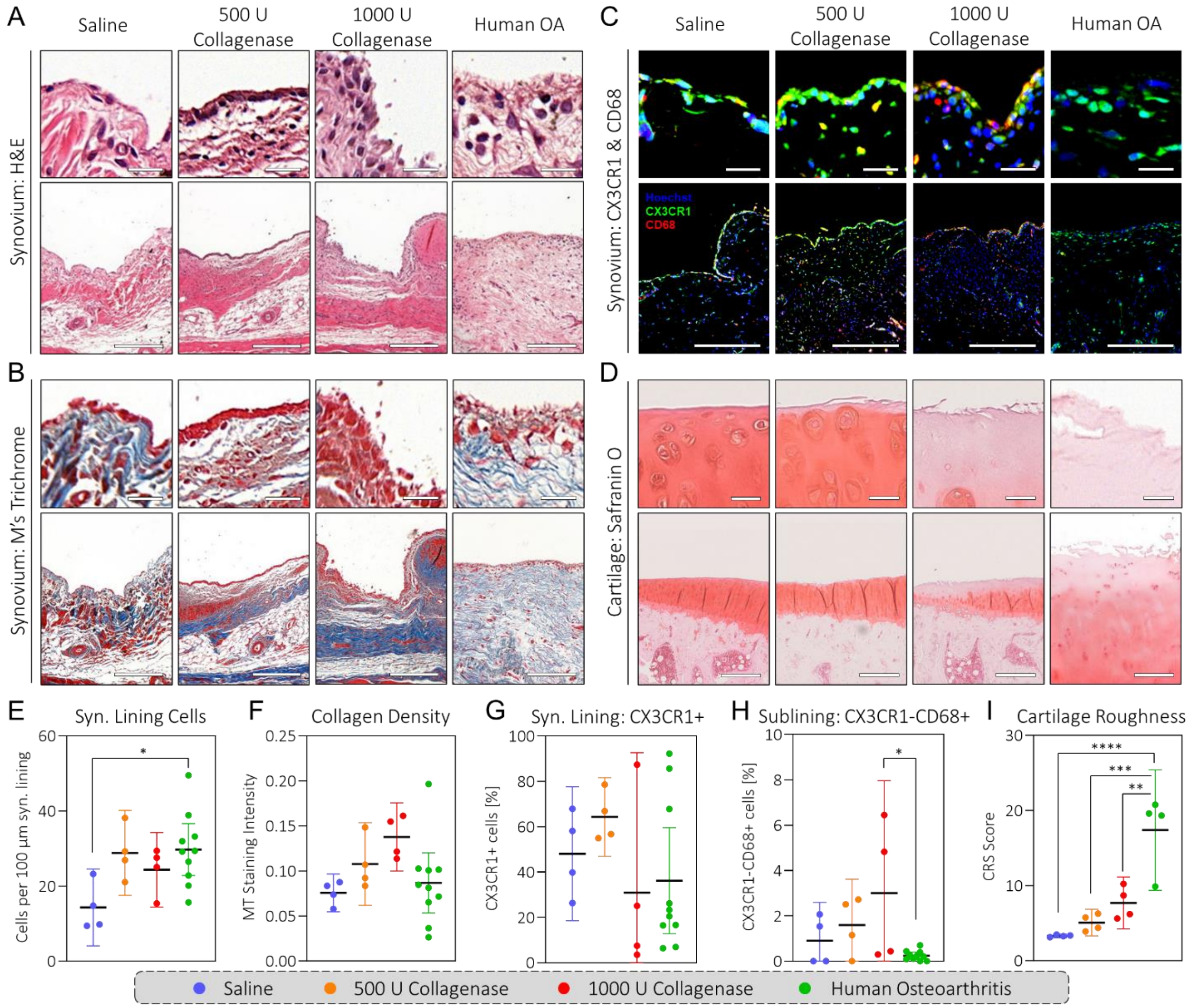

Figure S44 – Degenerative changes in rat synovium and cartilage resemble human OA tissues: Images of rat and human OA synovia stained with H&E (A), Masson's trichrome (B) and CD68/CX3CR1 (C) as well as cartilage stained with Safranin O (D). Scale bars: 25 µm (top), 250 µm (bottom). The number of synovium lining cells (E) and the CRS (I) are increased in all collagenase-injected rat groups and human OA. Collagen density is increased only in the rat collagenase-injected groups (F). Human OA synovia also show a decrease in CX3CR1+ cells in the synovial lining (G) but no increase in CD68+CX3CR1- cells (H) as the 1000 U collagenase group. N=4 (rat), N=10 (human synovium), N=4 (human cartilage).

References

1. Adães, S. *et al.* Intra-articular injection of collagenase in the knee of rats as an alternative model to study nociception associated with osteoarthritis. *Arthritis Res Ther* **16**, R10 (2014).
2. Adães, S. *et al.* Injury of primary afferent neurons may contribute to osteoarthritis induced pain: an experimental study using the collagenase model in rats. *Osteoarthritis and Cartilage* **23**, 914–924 (2015).
3. Jeong, J. *et al.* Anti-osteoarthritic effects of ChondroT in a rat model of collagenase-induced osteoarthritis. *BMC Complement Altern Med* **18**, 131 (2018).
4. Nirmal, P. *et al.* Influence of Six Medicinal Herbs on Collagenase-Induced Osteoarthritis in Rats. *Am. J. Chin. Med.* **41**, 1407–1425 (2013).
5. Nirmal, P. S., Jagtap, S. D., Narkhede, A. N., Nagarkar, B. E. & Harsulkar, A. M. New herbal composition (OA-F2) protects cartilage degeneration in a rat model of collagenase induced osteoarthritis. *BMC Complementary and Alternative Medicine* **17**, 6 (2017).
6. Shivnath, N. *et al.* Antiosteoarthritic effect of Punica granatum L. peel extract on collagenase induced osteoarthritis rat by modulation of COL-2, MMP-3, and COX-2 expression. *Environmental Toxicology* **36**, 5–15 (2021).
7. Won, J.-Y., Jeong, J.-W., Na, C.-S. & Kim, S.-J. Analgesic effects of ChondroT in collagenase-induced osteoarthritis rat model. *Journal of Korean Medicine Rehabilitation* **26**, 17–30 (2016).
